# Design, Synthesis, and Evaluation of the COX-2 Inhibitory Activities of New 1,3-Dihydro-*2H*-indolin-2-one Derivatives

**DOI:** 10.3390/molecules28124668

**Published:** 2023-06-09

**Authors:** Taohua Pan, Maofei He, Lulu Deng, Jiang Li, Yanhua Fan, Xiaojiang Hao, Shuzhen Mu

**Affiliations:** 1 College of Pharmacy, Guizhou University, Guiyang 550025, China; 2 State Key Laboratory of Functions and Applications of Medicinal Plants, Guizhou Medical University, Guiyang 550014, China; 3 The Key Laboratory of Chemistry for Natural Products of Guizhou Province, Chinese Academy of Sciences, Guiyang 550014, China; 4 Kunming Institute of Botany, Chinese Academy of Sciences (CAS), Kunming 650201, China

**Keywords:** oxindoles, α, β-unsaturated ketone, anti-inflammatory, COX-2 inhibitory activity

## Abstract

Thirty-three 1,3-dihydro-*2H*-indolin-2-one derivatives bearing α, β-unsaturated ketones were designed and synthesized via the Knoevenagel condensation reaction. The cytotoxicity, in vitro anti-inflammatory ability, and in vitro COX-2 inhibitory activity of all the compounds were evaluated. Compounds **4a**, **4e**, **4i**-**4j**, **and 9d** exhibited weak cytotoxicity and different degrees of inhibition against NO production in LPS-stimulated RAW 264.7 cells. The IC_50_ values of compounds **4a**, **4i**, and **4j** were 17.81 ± 1.86 μM, 20.41 ± 1.61 μM, and 16.31 ± 0.35 μM, respectively. Compounds **4e** and **9d** showed better anti-inflammatory activity with IC_50_ values of 13.51 ± 0.48 μM and 10.03 ± 0.27 μM, respectively, which were lower than those of the positive control ammonium pyrrolidinedithiocarbamate (PDTC). Compounds **4e**, **9h**, and **9i** showed good COX-2 inhibitory activities with IC_50_ values of 2.35 ± 0.04 µM, 2.422 ± 0.10 µM and 3.34 ± 0.05 µM, respectively. Moreover, the possible mechanism by which COX-2 recognized **4e**, **9h**, and **9i** was predicted by molecular docking. The results of this research suggested that compounds **4e**, **9h**, and **9i** might be new anti-inflammatory lead compounds for further optimization and evaluation.

## 1. Introduction

Inflammation is a protective response of the immune system to resist exogenous infection, repair injured tissue, and remove invading pathogens. However, disordered inflammation might result in numerous health problems, including heart disease, arthritis, depression, Alzheimer’s disease, and even cancer. Therefore, the development of effective anti-inflammatory drugs is very important. Nonsteroidal anti-inflammatory drugs (NSAIDs) are used as the primary remedy for fever, pain, and inflammation by inhibiting cyclooxygenase enzymes [1,2,3]. Cyclooxygenase-2 (COX-2), which is closely related to the occurrence and development of inflammation and cancer, is an important target for the development of nonsteroidal agents to treat inflammation [4,5,6]. The activity of COX-2 in normal tissue cells is extremely low, and when the cells are stimulated by inflammation, COX-2 expression levels in inflammatory cells can be increased to 10–80 times the normal level, causing an increase in the content of prostaglandin at the site of inflammation, thus leading to an inflammatory response and tissue damage. To date, COX-2 selective inhibitors have been widely used clinically to treat rheumatoid arthritis, osteoarthritis [7], toothache [8,9,10], postoperative pain [11], cancer, and other diseases [12]. Several drugs targeting COX-2 have made it to market. For example, the first selective inhibitor of COX-2, celecoxib, was approved for clinical use by the FDA in the United States as an NSAID in January 1991, and the second selective inhibitor, rofecoxib, was released in Europe in May of the same year. The successful marketing of these COX-2 inhibitors opened up a broad avenue for the subsequent research and development of COX-2 selective inhibitors. Although valdecoxib, celecoxib, and rofecoxib relieved inflammation without any gastric side effects [13,14], they resulted in a few cardiovascular issues, such as myocardial infarction and high blood pressure [15,16], which led to the withdrawal of both rofecoxib and valdecoxib from the market [17]. Consequently, searching for undescribed COX-2 inhibitors with little or no side effects is necessary.

Natural compounds and their synthesized analogues continue to be valuable sources for the discovery of scaffolds with high structural diversity and various bioactivities that can be directly developed or used as starting points for optimization to form new drugs [18]. Although natural products are normally valuable lead compounds, they are seldom used directly in clinical applications. Structural modifications are necessary, and changing the biological properties of natural products via structural modification is an important method that is commonly used in pharmaceutical chemistry [19,20]. Molecular hybridization, which involves the combination of two or more pharmacophores of bioactive scaffolds to generate a single molecular architecture with improved affinity and activity, is an emerging strategy in drug discovery [21]. This approach has recently gained increasing attention in the medical community and pharmaceutical industry, as it may provide opportunities to circumvent the growing, serious problem of drug resistance and increase the activity or pharmacological efficacy of known drugs or the bioactive constituents of hybrid molecules.

**Figure 1:**
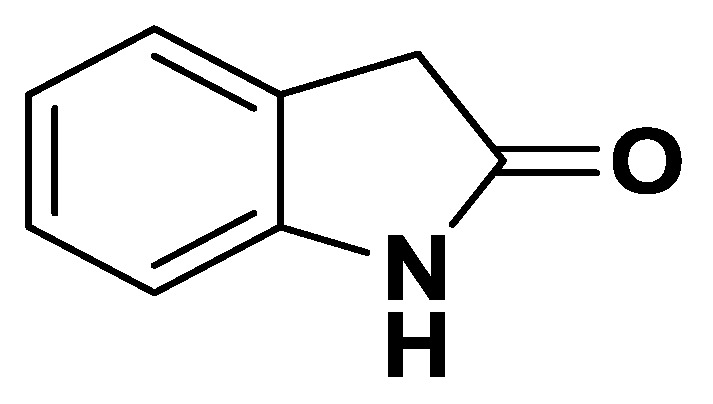
Structure of oxindole (1,3-dihydro-*2H*-indolin-2-one).

Oxindoles (such as 1,3-dihydro-*2H*-indole-2-one) (Figure 1), which are endogenous heteroaromatic organic compounds in animals and natural products of various plants [22,23], are the core structure of many biologically important compounds [24,25] and have been used to treat infections, cancer, arthritis, and other types of mild physical inflammation [26,27,28]. The role of oxindole as a chemical scaffold for fabricating and designing biological drug agents can be attributed to its ability to be modified by numerous chemical groups to generate novel biological functions. Oxindole is used for the preparation of the well-known drug Sunitinib by using Knoevenagel condensation reactions; thus, oxindole is a good synthesis module and has garnered interest among medicinal chemists.

The literature on oxindoles and their derivatives mainly focused on antitumor aspects, and the oxindoles had potential as the chemotherapeutic nucleus and had made significant recent progress in the synthetic development of oxindole as an antitumor agent. However, there was hardly related literature in which the oxindole derivatives were reported as COX-2 inhibitors or anti-inflammatory drugs. In order to find more biological properties of oxindoles and to discover lead compounds of anti-inflammatory drugs, the structures of oxindole derivatives were used as synthetic building blocks. A series of oxindoles incorporating α, β-unsaturated ketone derivatives (**4a**-**4x** and **9a**-**9i**) were designed and synthesized by splicing the active fragments to obtain new compounds. We performed molecular hybridization of oxindoles and 3-(trifluoromethyl)benzaldehyde or sulfonylphenyl by using the Michael addition reaction, and all the obtained compounds were evaluated for potential anti-inflammatory activity in LPS-stimulated murine macrophage RAW 264.7 cells in vitro. Furthermore, the COX-2 inhibitory activities of these compounds were evaluated in vitro using a Cyclooxygenase 2 Inhibitor Screening Kit.

## 2. Results and Discussion

**Scheme 1:**
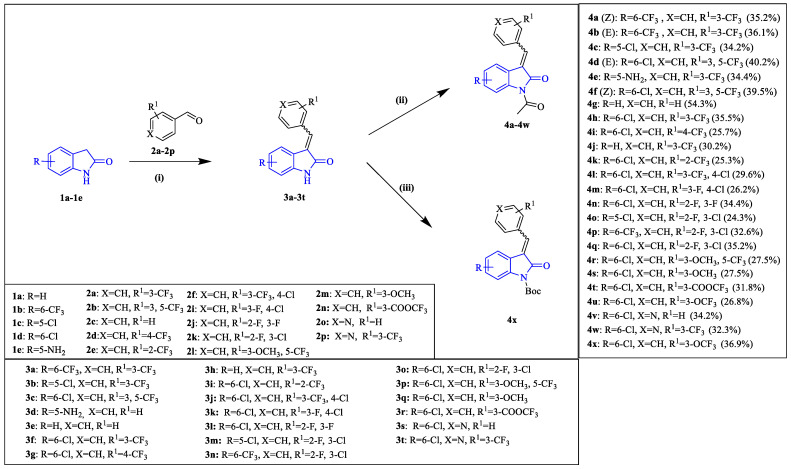
The synthesis of oxindole analogues containing α, β-unsaturated ketones. Reagents and conditions: (**i**) Piperidine, 80 °C, EtOH, 4 h; (**ii**) Ac_2_O, Na_2_CO_3_, THF, r.t., 24 h. (**iii**) DMAP, (Boc)_2_O, DCM, r.t. 8 h.

As the core structure of many biologically important natural compounds, oxindole is a promising heterocyclic ring system with various biological activities that has been well explored in humans [23]. To find more diverse active chemical entities, a molecular hybridization strategy was used for the synthesis of oxindole analogues containing α, β-unsaturated ketones. According to a previously reported procedure [29], twenty-four target compounds (**4a**-**4x**) were designed and synthesized by using oxindoles as the core skeleton via the Knoevenagel condensation reaction. The synthetic routes for the target compounds are shown in Scheme 1. First, commercially available oxindole derivatives (**1a**-**1e**) were reacted with the appropriate aromatic aldehyde (**2a**-**2n**) or pyridine-4-yl-carbaldehyde (**2o**-**p**) by using piperidine as a catalyst in EtOH to afford compounds **3a**-**3q**, and the reaction of compounds **3a**-**3q** with acetic anhydride occurred in the presence of Na_2_CO_3_ in THF. Then, the crude residues were purified by silica gel column chromatography to afford target compounds **4a**-**4w**. **3q** and di-*tert*-butyl decarbonate were added to DCM, and then the reaction was carried out at room temperature for 8 h with DMAP as the catalyst to afford the target compound **4x**.

The yields were low because the products were isomers, and we also observed some byproducts and unreacted starting materials. We then varied the temperature and used different solvents, such as methanol and ethanol. The desired products were still generated at room temperature when we used ethanol or methanol as solvents, but it required 24 h, and the yields did not improve. When the temperature was increased to 80 °C and ethanol was used as the solvent, the product was generated as a precipitate in 4 h. Therefore, we chose the conditions that were shown in Scheme 1. Compounds **4a** and **4b**, and compounds **4d** and **4f**, were differentiated by 2D-NOE NMR. The structures of the target compounds were characterized using high-resolution mass spectrometry (HRMS). The ^1^H NMR, ^13^C NMR, and HRMS data were consistent with the proposed structures. (The ^1^H- and ^13^C-NMR and HRMS spectra of all compounds **4a**-**4x** and **9a**-**9i** are shown in the Supplementary Materials).

**Scheme 2:**
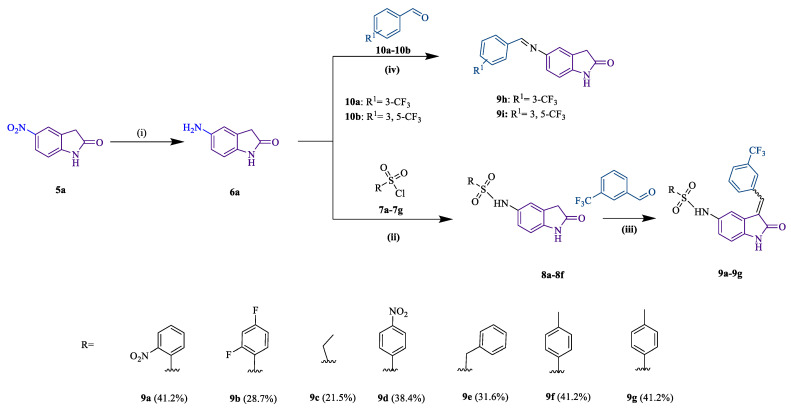
The synthesis of oxindole analogues **9a**-**9h**. Reagents and conditions: (**i**) Fe, NH_4_Cl, EtOH/H_2_O 4:1, reflux, 1.2 h; (**ii**) Pyridine, r.t., 12 h. (**iii**) Piperidine, 3-(trifluoromethyl)benzaldehyde, CH_3_OH, r.t., 24 h. (**iv**) C_2_H_5_OH, N_2_, reflux, 6 h.

In addition, through a literature investigation [30,31,32], we found that the introduction of appropriate groups (sulfonyl, sulfonyl phenyl, trifluoromethyl, or chlorine) at the appropriate position could increase the activity. Therefore, we introduced these groups by using different sulfonyl groups to protect the amine, and nine target compounds were designed and synthesized by using the Michael addition reaction. The general synthetic routes of the oxindole analogues containing α, β-unsaturated ketones in sulfonamide derivatives were illustrated in Scheme 2. Commercial material **5a** was treated with iron powder and ammonium chloride in EtOH/H_2_O to afford **6a**, which was reacted with the appropriate sulfonyl chloride to obtain compounds **8a**-**8f**. Subsequently, target compounds **9a**-**9g** were prepared by Knoevenagel condensation of compounds **8a**-**8f** with 3-(trifluoromethyl)-benzaldehyde in the presence of piperidine.

Compounds with imine or azomethine linkages are considered to have privileged pharmacophores due to their potential bioactivities, such as anti-inflammatory, anticancer, and antioxidant activities [33,34,35,36]. They constitute a vital class of organic compounds and intermediates used for the synthesis of biologically active compounds. Accounting for the widespread significance of imine pharmacophores, imine-linked substituted aromatic Schiff base derivatives **9h**-**9i** were synthesized as shown in Scheme 2.

**Figure 2:**
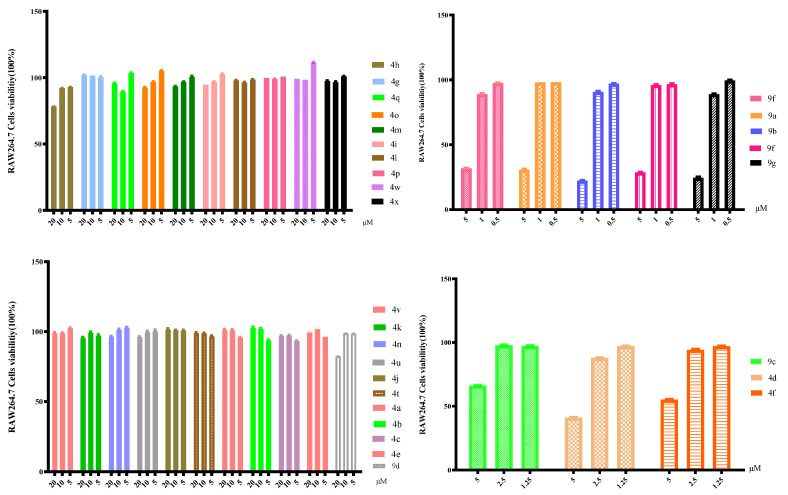
Cytotoxicity assay of all derivatives in vitro.

Since the excess production of nitric oxide (NO) in biological systems could lead to various diseases such as inflammation and atherosclerosis, the production of NO by immune cells has been used as a visual indicator for the presence and extent of inflammation [37,38,39]. The in vitro cytotoxicity activity of all the synthesized compounds (**4a**-**4x** and **9a**-**9g**) against mouse monocyte macrophage leukemia cells (RAW 264.7) was evaluated using the CCK-8 assay. The results of the cytotoxicity assay indicated that **9h**, **9i**, **4e**, and **4s** showed almost no cytotoxicity at concentrations of 100 μM. Most compounds exhibited no significant cytotoxicity within the concentration range of 0–40 µM (Figure 2).

**Figure 3:**
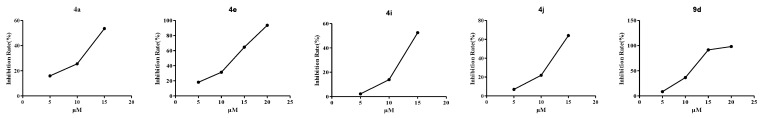
Inhibitory effects of compounds **4a**, **4e**, **4i**, **4j**, and **9d** on NO production in LPS-stimulated RAW 264.7 cells.

Lipopolysaccharide (LPS) is involved in the production of proinflammatory cytokines and induces an inflammatory response. To evaluate the in vitro anti-inflammatory activity, we examined the anti-inflammatory ability of all the generated compounds, particularly focusing on their NO production in LPS-stimulated RAW 264.7 cells (Table 1). Ammonium pyrrolidinedithiocarbamate (PDTC) was used as the positive control. Furthermore, cell viability was determined to explore whether the inhibition was due to the cytotoxicity of the tested compounds. Five compounds (**4a**, **4e**, **4i**-**4j**, and **9d**) exhibited weak cytotoxicity and obvious anti-inflammatory activity in a preliminary experiment. As shown in Figure 3, the results showed that the five compounds decreased NO production in LPS-activated RAW 264.7 cells in a concentration-dependent manner, and the inhibitory effect was significant only at high concentrations within the range of 0–40 µM.

As shown in Table 1, compounds **4a**, **4e**, **4i**, **4j**, and **9d** showed different degrees of inhibition against NO production in LPS-stimulated RAW 264.7 cells. The IC_50_ values of compounds **4a**, **4i**, and **4j** against RAW 264.7 cells were 17.81 ± 1.86 μM, 20.41 ± 1.61 μM, and 16.31 ± 0.35 μM, respectively. Furthermore, compounds **4e** and **9d** showed better anti-inflammatory activity with IC_50_ values of 13.51 ± 0.48 μM and 10.03 ± 0.27 μM, respectively, which were lower than those of the positive control PDTC. Compound **4e** exhibited no significant cytotoxicity at 100 µM. These results suggested that compound **4e** might be a new anti-inflammatory lead compound for further optimization and evaluation.

Cyclooxygenase-2 (COX-2) is closely related to the occurrence and development of inflammation and is a significant target for the development of nonsteroidal agents to treat inflammation. COX-2 is a bio-functional enzyme that catalyzes the biosynthesis of PGs during inflammation and has become a significant therapeutic target when searching for anti-inflammatory drugs. To explore whether the anti-inflammatory activities of these compounds were related to COX-2, the in vitro COX-2 inhibitory activity of all the synthesized compounds (**4a**-**4x** and **9a**-**9g**) was evaluated using a cyclooxygenase 2 inhibitor screening kit. Celecoxib was used as a positive control. First, a preliminary study was conducted to determine the most potent compounds based on cell viability. Six of the compounds showed significant COX-2 inhibitory activities. Then, the in vitro anti-inflammatory properties of these six compounds were extensively studied. As shown in Table 2, compounds **4e**, **9h**, and **9i** showed good COX-2 inhibitory activity with IC_50_ values ranging from 2.35 to 3.34 µM, while compound **4a** showed weak activity with an IC_50_ value of 19.9 ± 4.76 µM. The other oxindole derivatives had low inhibitory activity against COX-2, and their results are not listed in Table 2.

Although the activity of COX-2 in normal tissue cells is extremely low, some studies have confirmed that COX-2 also plays a role in the normal physiological functions of the human body and is not expressed only under pathological conditions such as inflammation, sepsis, and cell damage, as previously thought. Through cytotoxicity, in vitro anti-inflammatory ability, and COX-2 inhibitory activity tests, we found that compounds **9h** and **9i** had good inhibitory activity against COX-2 and no cytotoxicity, but they had no anti-inflammatory activity, which suggested that the COX-2 inhibitory activities of compounds **9h** and **9i** might not be related to anti-inflammatory activity.

According to the in vitro biological tests described above, compound **4e** was found to have a certain anti-inflammatory activity and COX-2 inhibitory activity, and it was implied that the compound **4e** with good activity had a relationship with the indole ring unit. The indole moiety belongs to an important pharmacophore core for the synthesis of anti-inflammatory activity, such as in the FDA-approved nonsteroidal anti-inflammatory drug indomethacin. Compared with existing drugs indomethacin with IC_50_ values of 0.026 μM (COX-2) [40] and celecoxib with IC_50_ values of 0.03 μM, compound **4e**, with IC_50_ values of 3.34 μM had a certain gap to become a clinical drug. However, compound **4e** was from the backbone of the natural product indolin-2-one, and it showed significant anti-inflammatory activity and COX-2 inhibitory activity, which have the potential to become a novel lead compound in anti-inflammatory drugs.

To investigate the interactions between the most potential compounds and the COX-2 active site, a molecular docking study with compounds **4e**, **9h**, and **9i** and a human COX-2 protein model was performed using crystal structure data for the COX-2 (PDB: ID 3LN1) active site obtained from the Protein Data Bank. The ligand docked into the 3LN1 structure by utilizing AutoDock Vina 1.1.2 and gave docking results. The more negative the Vina docking score was, the higher the binding affinity, and the compound with the lower energy score was taken as the subsequent analysis object. The docking results of compounds **9k**, **9i**, and **4e** with COX-2 are shown in Table 3. The pose with the most negative energy score was the subsequent object to analysis.

**Figure 4:**
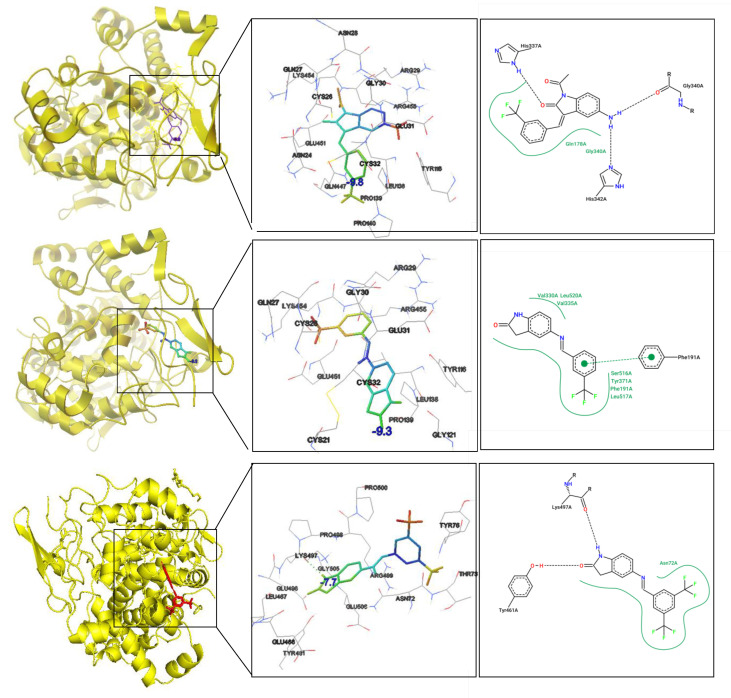
3D and 2D interaction diagrams between COX-2 (PDB: 3LN1) and compounds **4e**, **9h**, and **9i**. The right image is a 2D diagram of the interaction between compounds **4e**, **9h**, and **9i** with COX-2.

As shown in Figure 4, the results demonstrated that these compounds could bind well in a cavity composed of amino acid residues. Figure 4 shows a three-dimensional schematic diagram of the protein interaction. The yellow part represents the protein and amino acid residues within the protein that interact with the compound. Specifically, it was observed that the amino group at position 5 on the oxindole of **4e** formed two H bonds with the ketone carbonyl group of Gly−340 and the imidazole of His−342, and a hydrogen bond was formed between the ketone carbonyl group of the oxindole scaffold and His−337. In addition, the structure of the molecule could fit into pockets formed by amino acid residues. These interactions might contribute to the location of the compound within the hydrophobic COX-2 channel. Compound **9h** was effectively bound to the pockets formed by amino acid residues Val−330, Leu−529, Val−336, Ser−516, Tyr−371, Phe−191, and Leu−517, and the benzene ring attached to the trifluoromethyl moiety formed a π-π interaction with Phe−191. Moreover, compound **9i** bound to a cavity formed by amino acid residues Asn−72, Tyr−461, Lys−497, and Glu−560; the oxindole scaffold ketone carbonyl and Tyr−461 formed a hydrogen bond; and the hydrogen on the position 1 nitrogen formed a hydrogen bond with Lys−497. The docking poses of compounds **9h**, **9i**, and **4e** into the binding domain of COX-2 showed hydrogen bond interactions. These interactions were reflected in the docking score of pose 1 (−9.8, −9.3, and −8.7 kcal/mol) and supported the obtained in vitro COX-2 inhibitory activity.

In summary, the compounds **9h**, **9i**, and **4e** bound nicely into the pockets formed by amino acid residues and formed interactions; the binding interactions and energy binding scores were in agreement with the experimental and COX-2 inhibitory activities obtained for these compounds. The docking result for the compound **4e** was consistent with the biological activity test results above, suggesting that the anti-inflammatory activity of the compound may be related to COX-2. Therefore, compound **4e** had the potential to be a new anti-inflammatory lead compound for further optimization and evaluation.

## 3. Materials and Methods

### 3.1. General Chemistry

All reagents were commercially purchased, dried, and distilled following standard procedures. The solvents used for general chromatography and reactions were of analytical grade. Column chromatography separations were performed using silica gel (200–300 mesh). Thin-layer chromatography (TLC) was carried out on precoated silica gel GF_254_ plates (Qingdao Haiyang Chem. Ind. Ltd., P.R. Qingdao, China), and the spots were visualized with ultraviolet light (UV, Shanghai Jingke Ind. Co., Ltd., Shanghai, China) or by heating the plates dipped in 5% H_2_SO_4_ in ethanol or 5% phosphomolybdic acid hydrate in an ethanol solution. ^1^H NMR (600 MHz) and ^13^C NMR (150 MHz) spectra were recorded on a Bruker AVANCE NEO NMR spectrometer in CDCl_3_, CD_3_OD, CD_3_OCD_3_, and DMSO-*d_6_* (Anhui Ze Sheng Tech. Co., Ltd., Anhui, China). Chemical shifts are expressed as *δ* values (ppm) using tetramethylsilane as the internal standard, and the following abbreviations were used: s = singlet, d = doublet, t = triplet, q = quartet, m = multiplet, and br = broad peak. Coupling constants, *J*, are reported in Hertz. High-resolution mass spectra were obtained with a Bruker micro TOF-Q mass spectrometer. The melting point (m.p.) values of the solid final compounds were determined using a WRX-4 micromelting point instrument.

### 3.2. General Procedure for the Synthesis of Oxindole Analogues Incorporating *α*, *β*-unsaturated Ketones (***4a***-***4x***)

First, compounds **1a**-**1e** (3 mmol) and **2a**-**2p** (3.3 mmol) were placed in a round-bottom flask, and piperidine (0.6 mmol) was added. After dissolution, anhydrous ethanol (8 mL) was added to the above mixture. The solution was vigorously stirred at 80 °C for 4 h on a magnetic stirrer. Then, after a precipitate was generated, the solid was filtered and washed with ethanol to obtain intermediates **3a**-**3u**. Then, acetic anhydride (15 mmol) and NaCO_3_ (15 mmol) were added to a solution of **3a**-**3u** (3 mmol) in tetrahydrofuran. After stirring at room temperature for 24 h, the mixture was diluted with H_2_O and extracted with ethyl acetate (3 × 50 mL). Then, the combined organic layers were washed with a saturated sodium chloride solution (3 × 50 mL) and dried over anhydrous Na_2_SO_4_. The filtrate was concentrated under reduced pressure, and the crude residue was purified by silica gel column chromatography (petroleum ether and ethyl acetate) or with a Sephadex LH-20 column (CH_3_OH) to afford target compounds **4a**-**4w**. **3q** (3 mmol) and di-*tert*-butyl decarbonate (3 mmol) were added to DCM, and then the reaction was carried out at room temperature for 8 h with DMAP (0.3 mmol) as the catalyst to afford the target compound **4x.**

Compound **4a**: Yield: 35.2%, yellow solid, m.p. 131.2–132.1 °C; ^1^H NMR (600 MHz, CDCl_3_) *δ* 8.61 (s, 1H), 8.41 (s, 1H), 8.39 (d, *J* = 8.0 Hz, 1H), 7.76 (s, 1H), 7.74 (s, 1H), 7.71 (s, 1H), 7.65 (s, 1H), 7.55 (d, *J* = 0.8 Hz, 1H), 2.77 (s, 3H) ppm; ^13^C NMR (150 MHz, CDCl_3_) *δ* 170.92, 165.44, 139.10, 138.95, 134.91, 133.37, 131.70 (q, *J* = 32.5 Hz), 131.06 (q, *J* = 32.8 Hz), 130.95, 129.02, 128.80 (q, *J* = 3.9 Hz), 127.84 (q, *J* = 3.7 Hz), 127.32, 123.80 (q, *J* = 272.5 Hz), 121.88 (q, *J* = 4.1 Hz), 119.08, 119.08, 114.03 (q, *J* = 4.1 Hz), 26.88 ppm; ESI-HRMS m/z calculated for C_19_H_11_F_6_NO_2_Na [M + Na]+ 422.05927, found 422.05862.

Compound **4b**: Yield: 36.1%, yellow solid, m.p. 91.3–92.8 °C; ^1^H NMR (600 MHz, CDCl_3_) *δ* 8.65 (s, 1H), 8.01 (s, 1H), 7.90 (s, 1H), 7.84 (d, *J* = 7.6 Hz, 1H), 7.78 (d, *J* = 7.9 Hz, 1H), 7.69 (t, *J* = 7.8 Hz, 1H), 7.64 (d, *J* = 8.1 Hz, 1H), 7.36–7.30 (m, 1H), 2.80 (s, 3H) ppm; ^13^C NMR (150 MHz, CDCl_3_) *δ* 170.67, 167.44, 140.66, 139.06, 134.64, 132.30 (q, *J* = 32.6 Hz), 132.17, 131.68 (q, *J* = 32.9 Hz), 129.68, 127.03 (q, *J* = 3.7 Hz), 126.51, 125.91 (q, *J* = 3.7 Hz), 124.49 (d, *J* = 8.5 Hz), 124.30, 122.68 (d, *J* = 8.7 Hz), 122.14, 121.62 (q, *J* = 4.0 Hz), 114.09 (q, *J* = 3.9 Hz), 26.77 ppm; ESI-HRMS *m*/*z* calculated for C_19_H_11_F_6_NO_2_Na [M + Na]^+^ 422.05917, found 422.05862.

Compound **4c**: Yield: 34.2%, yellow amorphous solid, m.p. 139.1–142.8 °C; ^1^H NMR (600 MHz, CDCl_3_) *δ* 8.31 (d, *J* = 8.8 Hz, 1H), 7.92 (s, 1H), 7.90 (s, 1H), 7.83 (d, *J* = 7.8 Hz, 1H), 7.78 (d, *J* = 7.9 Hz, 1H), 7.69 (t, *J* = 7.8 Hz, 1H), 7.54 (d, *J* = 2.1 Hz, 1H), 7.34 (dd, *J* = 2.2, 8.8 Hz, 1H), 2.78 (s, 3H) ppm; ^13^C NMR (150 MHz, CDCl_3_) *δ* 170.58, 167.58, 138.99, 137.70, 134.66, 132.11, 131.66 (q, *J* = 32.3 Hz), 130.61, 130.24, 129.65, 126.91 (q, *J* = 3.7 Hz), 126.61, 125.97 (q, *J* = 3.8 Hz), 122.38 (d, *J* = 97.9 Hz), 122.06, 118.14, 118.13, 26.82 ppm; ESI-HRMS *m*/*z* calculated for C_18_H_11_ClF_3_NO_2_Na [M + Na]^+^ 388.03226, found 388.03226.

Compound **4d**: Yield: 40.2%, yellow amorphous solid, m.p. 119.4–121.7 °C; ^1^H NMR (600 MHz, CDCl_3_) *δ* 8.40 (d, *J* = 2.0 Hz, 1H), 8.07 (s, 2H), 7.99 (s, 1H), 7.84 (s, 1H), 7.33 (d, *J* = 8.3 Hz, 1H), 7.06 (dd, *J* = 2.0, 8.4 Hz, 1H), 2.77 (s, 3H) ppm; ^13^C NMR (150 MHz, CDCl_3_) *δ* 170.43, 167.37, 141.65, 137.35, 136.33, 133.88, 132.59 (q, *J* = 33.5, 34.0 Hz), 129.02, 129.00, 128.07, 125.46 (d, *J* = 45.0 Hz), 124.99, 123.80, 123.35 (p, *J* = 3.7 Hz), 122.55, 121.99, 118.44 (q, *J* = 197.3 Hz), 117.78, 26.74 ppm; ESI-HRMS *m*/*z* calculated for C_19_H_10_ClF_6_NO_2_Na [M + Na]^+^ 456.01996, found 456.01965.

Compound **4e**: Yield: 34.4%, red solid, m.p. 165.9–166.2 °C. ^1^H NMR (600 MHz, CDCl_3_) *δ* 8.15 (d, *J* = 8.7 Hz, 1H), 7.92 (dq, *J* = 1.0, 2.0 Hz, 1H), 7.81 (d, *J* = 9.5 Hz, 2H), 7.76–7.70 (m, 1H), 7.64 (t, *J* = 7.9 Hz, 1H), 6.87 (d, *J* = 2.4 Hz, 1H), 6.70 (dd, *J* = 2.5, 8.7 Hz, 1H), 3.57 (s, 2H), 2.75 (s, 3H) ppm; ^13^C NMR (150 MHz, DMSO) *δ* 165.56, 163.61, 138.53, 130.86, 130.60, 128.52, 127.53, 126.54 (q, *J* = 32.9 Hz), 124.66, 124.65, 123.30, 121.50 (q, *J* = 3.8 Hz), 121.14 (q, *J* = 3.7 Hz), 117.40, 113.21, 112.66, 103.70, 21.95 ppm; ESI-HRMS *m*/*z* calculated for C_18_H_13_F_3_N_2_O_2_Na [M + Na]^+^ 456.02078, found 456.01965.

Compound **4f**: Yield: 39.5%, yellow amorphous solid, m.p. 163.4–164.0 °C; ^1^H NMR (600 MHz, CDCl_3_) *δ* 8.55 (d, *J* = 1.7 Hz, 2H), 8.36 (d, *J* = 1.9 Hz, 1H), 7.96 (s, 1H), 7.59 (s, 1H), 7.55 (d, *J* = 8.2 Hz, 1H), 7.27 (dd, *J* = 1.9, 8.2 Hz, 1H), 2.74 (s, 2H) ppm; ^13^C NMR (150 MHz, CDCl_3_) *δ* 170.70, 165.54, 139.97, 136.61, 134.74, 134.26, 131.80 (q, *J* = 34.0, 34.5 Hz),131.24 (d, *J* = 3.7 Hz), 127.35, 125.34, 124.03, 123.75 (q, *J* = 4.0 Hz), 123.12(q, *J* = 273.31 Hz), 122.02, 120.11, 120.10, 117.48, 26.85, 1.03 ppm; ESI-HRMS *m*/*z* calculated for C_19_H_10_ClF_6_NO_2_Na [M + Na]^+^ 456.02078, found 456.01965.

Compound **4g**: Yield, 54.4%, yellow solid, m.p. 145–146 °C; ^1^H NMR (600 MHz, CDCl_3_) *δ* 8.34 (d, *J* = 8.4 Hz, 1H), 7.91(s, 1H), 7.72 (d, *J* = 7. 2 Hz, 1H), 7.66 (d, *J* = 7.2 Hz, 2H), 7.50 (ddd, *J* = 8.4 Hz, 7.8 Hz, 6.0 Hz, 3H), 7.37–7.32 (m, 1H), 7.05 (td, *J* = 7.8Hz, 1.2 Hz, 1H), 2.79 (s, 3H) ppm; ^13^C NMR (150 MHz, CDCl_3_) *δ* 170.91, 168.62, 140.28, 138.71, 134.42, 130.28, 130.07, 129.21, 128.81, 124.49, 122.16, 121.88, 116.75, 27.00, 26.93 ppm; ESI-HRMS *m*/*z* calculated for C_17_H_13_NO_2_Na [M + Na]^+^ 286.0838, found 286.0831.

Compound **4h**: Yield: 31.5%, yellow solid, m.p. 142.1–143.4 °C; ^1^H NMR (600 MHz, CDCl_3_) *δ* 8.41 (d, *J* = 1.8 Hz, 1H), 7.87 (d, *J* = 1.8 Hz, 2H), 7.81 (d, *J* = 12.0 Hz, 1H), 7.75 (d, *J* = 12.0 Hz, 1H), 7.66 (t, *J* = 6.0 Hz, 1H), 7.46 (d, *J* = 6.0 Hz, 1H), 7.05 (dd, *J* = 12.0 Hz, 1.8 Hz, 1H), 2.74 (s, 3H) ppm; ^13^C NMR (150 MHz, CDCl_3_) *δ* 170.84, 165.69, 139.53, 136.87, 134.59, 134.59, 133.67, 130.94 (d, *J* = 32.8 Hz), 128.91, 128.48 (q, *J* = 3.9 Hz), 127.31 (q, *J* = 3.7 Hz), 125.61, 125.16, 123.85 (q, *J* = 14.2 Hz, 272.5 Hz), 122.65, 119.78, 117.32, 26.86. ESI-HRMS *m*/*z* calculated for C_18_H_11_NO_2_F_3_ClNa [M + Na]^+^ 388.0323, found 388.0311.

Compound **4i**: Yield: 25.7%, yellow solid, m.p. 142.9–144.1 °C; ^1^H NMR (600 MHz, CDCl_3_) *δ* 8.43 (d, *J* = 1.8 Hz, 1H), 7.84 (s, 1H), 7.59–7.51 (m, 3H), 7.49 (s, 1H), 7.35 (d, *J* = 6.6 Hz, 1H), 7.06 (dd, *J* = 8.4 Hz, 1.8 Hz, 1H), 2.78(s, 3H); ^13^C NMR (150 MHz, CDCl_3_) *δ* 170.91, 168.22, 140.28, 138.71, 134.48, 135.28, 130.07,129.21 (q, *J* = 59.7 Hz), 128.81(q, *J* = 59.7 Hz), 126.07, 124.49, 122.16, 121.88, 116.75, 77.27, 77.06, 76.83, 26.92 ppm. ESI-HRMS *m*/*z* calculated for C_18_H_11_NO_2_F_3_ClNa [M + Na]^+^ 388.0323, found 388.0311.

Compound **4j**: Yield: 30.2%, yellow solid, m.p.121.5–123.3 °C; ^1^H NMR (600 MHz, CDCl_3_) *δ* 8.36 (d, *J* = 8.4 Hz, 1H), 7.92 (s, 1H), 7.87 (s, 1H), 7.84 (d, *J* = 7.8 Hz, 1H), 7.74 (d, *J* = 7.8 Hz, 1H), 7.65 (dd, *J* = 18.0 Hz, 8.4 Hz, 1H), 7.56 (d, *J* = 7.8 Hz, 1H), 7.38(t, *J* = 7.8 Hz, 1H), 7.07 (t, *J* = 7. 8 Hz, 1H), 2.80 (s, 3H); ^13^C NMR (150 MHz, CDCl_3_) *δ* 170.78, 168.21, 140.66, 136.05, 135.27 (d, *J* = 119.0 Hz), 132.19, 130.93 (d, *J* = 32.7 Hz), 129.45, 127.61, 126.43 (q, *J* = 3.7 Hz), 125.89 (q, *J* = 3.8 Hz), 124.68, 122.08, 121.28, 117.00, 26.92 ppm; ESI-HRMS *m*/*z* calculated for C_18_H_12_NO_2_F_3_Na [M + Na]^+^ 354.0712, found 354.0705.

Compound **4k**: Yield: 25.3%, yellow solid, m.p. 131.0–133.6 °C; ^1^H NMR (600 MHz, CDCl_3_) *δ* 8.66 (d, *J* = 6.0 Hz, 1H), 7.96 (s, 1H), 7.56–7.60 (m, 3H), 7.40 (d, *J* = 8. 4 Hz, 1H), 7.04 (dd, *J* = 8.4 Hz, 1.8 Hz, 2H), 2.98 (s, 3H) ppm; ^13^C NMR (150 MHz, CDCl_3_) *δ* 170.65, 167.13, 156.79, 155.10, 140.73, 133.41, 132.44 (d, *J* = 16.4 Hz), 132.38, 130.26, 128.16, 127.67, 124.91 (d, *J* = 4.5 Hz), 124.28, 124.50 (d, *J* = 14.6 Hz), 122.64, 121.70 (q, *J* = 4.0 Hz), 119.60, 114.03 (q, *J* = 4.1 Hz), 26.79; ESI-HRMS *m*/*z* calculated for C_18_H_11_NO_2_FCl_3_Na [M + Na]^+^ 388.0323, found 388.0311.

Compound **4l**: Yield: 29.6%, yellow solid, m.p. 142.3–144.1 °C; ^1^H NMR (600 MHz, CDCl_3_) *δ* 8.41 (d, *J* = 1.8 Hz, 1H), 8.36 (d, *J* = 1.8 Hz, 1H), 8.33 (dd, *J* = 8.4 Hz, 1.8 Hz, 1H), 7.62 (d, *J* = 8.4 Hz, 1H), 7.55–7.51 (m, 2H), 7.26 (dd, *J* = 8.4 Hz, 1.8 Hz, 1H), 2.75 (s, 3H) ppm; ^13^C NMR (150 MHz, CDCl_3_) *δ* 170.76, 165.76, 139.61, 135.78 (d, *J* = 88.6 Hz), 135.48, 132.22, 131.86, 131.56, 130.89 (q, *J* = 5.4 Hz), 128.58 (q, *J* = 31.7 Hz), 125.95, 125.24, 124.93, 122.48, 121.68, 119.84, 117.38, 26.87 ppm; ESI-HRMS *m*/*z* calculated for C_18_H_11_NO_2_F_3_Cl_2_Na [M + H]^+^ 400.0113, found 400.0121.

Compound **4m**: Yield: 26.2%, yellow solid, m.p. 151.6–153.3 °C; ^1^H NMR (600 MHz, CDCl_3_) *δ* 8.35 (d, *J* = 1.8 Hz, 1H), 8.21 (dd, *J* = 10.8 Hz, 1.8 Hz, 1H), 7.81–7.71 (m, 1H), 7.49 (dd, *J* = 15.6 Hz, 7.2 Hz, 3H), 7.24 (dd, *J* = 8.4 Hz, 1.8 Hz, 1H), 2.75 (s, 3H) ppm; ^13^C NMR (150 MHz, CDCl_3_) *δ* 170.80, 165.77, 139.44, 136.24, 136.22, 135.82, 133.24 (d, *J* = 7.6 Hz), 130.48, 128.81 (d, *J* = 7.6 Hz), 128.78, 125.15, 122.68, 119.69, 119.36, 119.21, 117.31, 26.88, 26.79 ppm; ESI-HRMS *m*/*z* calculated for C_18_H_12_NO_2_FCl_2_ [M + H]^+^ 350.0547, found 350.0422.

Compound **4n***:* Yield: 34.4%, yellow soild, m.p. 136.3–137.2 °C; ^1^H NMR (600 MHz, CDCl_3_) *δ* 8.41 (d, *J* = 1.8 Hz, 1H), 7.83 (s, 1H), 7.40 (d, *J* = 7.8 Hz, 2H), 7.35–7.29 (m, 1H), 7.26–7.18 (m, 1H), 7. 08 (dd, *J* = 8.4 Hz, 1.8 Hz, 1H), 2.79 (s, 3H); ^13^C NMR (150 MHz, CDCl_3_) *δ* 170.59, 167.51, 141.34, 136.73, 129.50 (t, *J* = 3.1 Hz), 127.91, 124.91, 124.71 (d, *J* = 3.7 Hz), 124.70 (q, *J* = 4.1 Hz), 124.47, 123.26, 120.31, 119.72, 119.16, 119.05, 117.47, 26.79 ppm; ESI-HRMS *m*/*z* calculated for C_17_H_10_NO_2_F_2_ClNa [M + Na]^+^ 356.0260, found 356.0250.

Compound **4o***:* Yield: 24.3%, yellow solid, m.p. 141.3–142.4 °C; ^1^H NMR (600 MHz, CDCl_3_) *δ* 8.30 (d, *J* = 9.0 Hz, 1H), 7.87 (s, 1H), 7.59–7.55 (m, 2H), 7.43 (d, *J* = 1.8 Hz, 1H), 7.36–7.31 (m, 1H), 7.26 (t, *J* = 7.8 Hz, 1H), 2.78 (s, 3H); ^13^C NMR (150 MHz, CDCl_3_) *δ* 170.58, 167.48, 141.32, 136.71, 132.48, 129.87, 128.15, 127.80, 125.23, 124.92, 124.78, 123.21, 120.28, 119.71, 117.46, 26.78 ppm; ESI-HRMS *m*/*z* calculated for C_18_H_13_NO_2_FCl_2_ [M + H]^+^ 350.0547, found 350.0422.

Compound **4p***:* Yield: 32.6%, yellow soild, m.p. 120.1–122.4 °C; ^1^H NMR (600 MHz, CDCl_3_) *δ* 2.81 (s, 3H), 7.25 (t, *J =* 7.8 Hz, 1H) 7.36 (dd, *J =* 0.6, 8.4 Hz, 1H), 7.56 (d, *J =* 7.8 Hz, 2H), 7.58 (d, *J =* 8.4 Hz, 1H), 7.95 (s, 1H), 8.66 (s, 1H) ppm; ^13^C NMR (150 MHz, CDCl_3_) *δ* 170.65, 167.13, 155.94 (d, *J* = 254.8 Hz), 140.73, 133.41, 132.88, 132.38 (d, *J* = 16.7 Hz), 130.26, 128.16, 127.67, 124.92 (d, *J* = 4.5 Hz), 124.28, 123.57, 122.64, 121.70 (q, *J* = 4.0 Hz), 119.60, 114.05, 26.79 ppm; ESI-HRMS *m*/*z* calculated for C_16_H_13_O_2_N_2_Cl [M + H]^+^ 406.0582, found: 406.0576.

Compound **4q***:* Yield: 35.2%, yellow solid, m.p. 135.3–136.2 °C; ^1^H NMR (600 MHz, CDCl_3_) *δ* 8.40 (d, *J* = 1.8 Hz, 1H), 7.81(s, 1H), 7.58–7.53 (m, 2H), 7.37 (d, *J* = 8.4 Hz, 1H), 7.27–7.19 (m, 1H), 7.06 (dd, *J* = 8.4 Hz, 1.8 Hz, 1H), 2.78 (s, 3H) ppm; ^13^C NMR (150 MHz, CDCl3) *δ* 170.69, 167.48, 139.09, 137.61,132.80, 131.39 (q, *J* = 150 Hz), 131.16 (d, *J* = 2.8 Hz), 130.69, 130.13 (d, *J* = 5.6 Hz), 128.04, 127.68, 124.88, 124.85 (q, *J* = 104 Hz), 122.70, 122.40, 119.55, 118.07, 26.79 ppm; ESI-HRMS *m*/*z* calculated for C_18_H_10_NO_2_FCl_2_Na [M + Na]^+^ 350.0547, found 350.0422.

Compound **4r***:* Yield: 27.5%, yellow solid, m.p. 140.5–142.4 °C; ^1^H NMR (600 MHz, CDCl_3_) *δ* 8.40 (d, *J* = 1.8 Hz, 1H), 7.83 (s, 1H), 7.52–7.49 (m, 1H), 7.23 (s, 2H), 7.06 (dd, *J* = 8.4 Hz, 1.8 Hz, 1H), 3.91 (s, 3H), 2.77(s, 3H) ppm; ^13^C NMR (150 MHz, CDCl_3_) *δ* 170.74, 167.82, 160.29, 159.66, 141.19, 136.58, 135.37 (d, *J* = 133.0 Hz), 132.73 (q, *J* = 32.8 Hz), 126.83, 124.84, 122.99, 119.70 (t, *J* = 4.0 Hz), 117.91 (q, *J* = 4.0 Hz), 117.52, 117.27, 113.49, 112.23 (q, *J* = 3.7 Hz), 55.80, 26.79; ESI-HRMS *m*/*z* calculated for C_19_H_15_NO_3_F_3_Cl [M + H]^+^ 396.0609, found 396.0616.

Compound **4s***:* Yield: 32.6%, yellow solid; m.p. 141.3–143.2 °C; ^1^H NMR (600 MHz, CDCl_3_) *δ* 8.39 (d, *J* = 1.8 Hz, 1H), 7.88 (s, 1H), 7.67 (d, *J* = 8.4 Hz, 1H), 7.42 (t, *J* = 7.8 Hz, 1H), 7.21 (d, *J* = 7.2 Hz, 1H), 7.13(s, 1H), 7.06–6.99(m, 2H), 3.86(s, 3H), 2.77(s, 3H) ppm; ^13^C NMR (150 MHz, CDCl_3_) *δ* 170.73, 168.22, 159.84, 140.92, 139.07, 135.88, 135.38, 135.09, 130.05, 125.32, 124.64, 123.10, 121.49, 117.28, 116.10, 114.23, 55.42, 26.82 ppm; ESI-HRMS *m*/*z* calculated for C_18_H_14_NO_3_ClNa [M + Na]^+^ 350.0554, found 350.0546.

Compound **4t***:* Yield: 31.8%, yellow solid, m.p. 138.4–139.5 °C; ^1^H NMR (600 MHz, CDCl_3_) *δ* 8.39 (d, *J* = 1.8 Hz, 1H), 8.11 (d, *J* = 8.4 Hz, 2H), 7.89 (s, 1H), 7.66 (d, *J* = 8.4 Hz, 2H), 7.53 (d, *J* = 8.4 Hz, 1H), 7.03 (dd, *J* = 8.4 Hz, 1.8 Hz, 1H), 2.77 (s, 3H), 1.65 (s, 9H); ^13^C NMR (150 MHz, CDCl_3_) *δ* 170.63, 167.94, 164.91, 141.15,138.12, 137.60, 136.37, 133.32, 129.93, 128.87, 126.40, 124.78, 123.04, 119.95, 117.41, 81.72, 28.19, 26.80 ppm; ESI-HRMS *m*/*z* calculated for C_22_H_20_NO_4_ClNa [M + Na]^+^ 420.0946, found 420.0963.

Compound **4u***:* Yield: 26.8%, yellow solid, m.p. 135.4–136.6 °C; ^1^H NMR (600 MHz, CDCl_3_) *δ* 8.41 (d, *J* = 1.8 Hz, 1H), 7.84 (s, 1H), 7.59–7.51 (m, 3H), 7.49 (s, 1H), 7.35 (d, *J* = 6.6 Hz, 1H), 7.06 (dd, *J* = 8.4 Hz, 1.8 Hz, 1H), 2.78 (s, 3H); ^13^C NMR (150 MHz, CDCl_3_) *δ* 170.61, 167.89, 141.22, 136.58, 136.53, 136.12, 130.60, 127.48, 126.50, 124.81, 122.94, 122.53, 121.28, 119.74, 117. 49, 26.87, 26.80; ESI-HRMS *m*/*z* calculated for C_18_H_12_NO_3_F_3_Cl [M + H]^+^ 382.0452, found 382.0441.

Compound **4v***:* Yield: 34.2%, yellow soild, m.p. 137.1–139.5 °C; ^1^H NMR (600 MHz, CDCl_3_) *δ* 2.77 (s, 3H), 7.04 (dd, *J* = 1.8, 8.4 Hz, 1H), 7.40 (d, *J* = 8.4 Hz, 1H), 7.46 (d, *J* = 5.4 Hz, 2H), 7.76 (s, 1H), 8.39 (s, 1H), 8.78 (d, *J* = 6.0 Hz, 2H) ppm; ^13^C NMR (150 MHz, CDCl_3_) *δ* 26.7, 117.5, 119.3, 122.6, 123.3, 124.9, 128.1, 134.6, 137.1, 141.5, 142.2, 150.6, 167.5, 170.5 ppm. ESI-HRMS *m*/*z* calculated for C_16_H_13_O_2_N_2_Cl [M + H]^+^ 299.0582, found: 299.0576.

Compound **4w**: Yield: 32.3%, yellow solid, m.p. 143.1–144.5 °C; ^1^H NMR (600 MHz, CDCl_3_) *δ* 8.79 (d, *J* = 6.0 Hz, 2H), 8.39 (s, 1H), 7.46 (d, *J* = 5.4 Hz, 1H), 7.40 (d, *J* = 8. 4 Hz, 1H), 7.04 (dd, *J* = 8.4 Hz, 1.8 Hz, 2H), 2.77 (s, 3H); ^13^C NMR (150 MHz, CDCl_3_) *δ* 170.80, 165.77, 158.52 (q, *J* = 248.8 Hz), 156.87, 139.45, 136.24, 135.82, 133.38, 130.48, 128.81 (d, *J* = 3.4 Hz), 125.15, 122.68 (q, *J* = 18.0 Hz, 130.3 Hz), 119.69, 119.36, 117.31, 26.88, 26.80 ppm; ESI-HRMS *m*/*z* calculated for C_17_H_12_N_2_O_2_F_3_Cl [M + H]^+^ 367.0456, found 367.0447.

Compound **4x**: Yield: 36.9%, yellow solid; m.p. 151.4–152.2 °C; ^1^H NMR (600 MHz, CDCl_3_) *δ* 8.03 (d, *J* = 1.8 Hz, 1H), 7.86 (d, *J* = 4.2 Hz, 2H), 7.78 (d, *J* = 7.8 Hz, 1H), 7.73 (d, *J* = 7.8 Hz, 1H), 7.64 (t, *J* = 7.8 Hz, 1H), 7.43 (d, *J* = 8.4 Hz, 1H), 7.01 (dd, *J* = 8.4 Hz, 1.8 Hz, 1H), 1.70 (s, 9H); ^13^C NMR (150 MHz, CDCl_3_) *δ* 165.79, 150.21, 141.23, 136.46, 136.09, 135.23, 132.16, 129.51, 126.44 (q, *J* = 3.9 Hz), 125.78 (q, *J* = 3.7 Hz), 125.26, 125.13, 124.05, 122.95, 119.35, 116.18, 85.02, 28.10; ESI-HRMS *m*/*z* calculated for C_21_H_17_NO_3_F_3_ClNa [M + Na]^+^ 446.0741, found 446.0733.

### 3.3. General Procedure for the Synthesis of ***9a***-***9g***

First, 5-nitroindolin-2-one (**5a**, 2.81 nmol) and ammonium chloride (1.67 mmol) were placed in a 25-mL round-bottom flask, and then a mixed solution of ethanol and H_2_O (*v*/*v* 4:1) was added. After dissolution, iron powder (14 mmol) was added to the mixture and refluxed at 100 °C for 1.2 h. After the reaction was complete, the solution was filtered while hot and concentrated under reduced pressure to afford the product 5-aminoindolin-2-one (**6a**). Then, compounds **7a**-**7g** were added to a solution of compound **6a** in pyridine at room temperature for 12 h. After the reaction was complete, aqueous hydrochloric acid (pH 1–2) was added to the aqueous solution until no pyridine remained. Then, the aqueous solution was extracted with ethyl acetate (3 × 50 mL), and the combined organic layer was dried over anhydrous sodium sulfate and filtered. The solvent was evaporated in vacuo, and the residue was purified by silica gel column chromatography to obtain products **8a**-**8f**. A mixture of **8a**-**8f** and piperidine in methyl alcohol was stirred at room temperature under nitrogen protection, and an appropriate amount of 3-(trifluoromethyl)benzaldehyde was added. After the reaction was complete, the solvent was evaporated in vacuo, and the residue was purified by silica gel column chromatography and a Sephadex LH-20 column (CH_3_OH) to obtain target products **9a**-**9g**.

Compound **9a**: Yield: 31.4%, orange powder, m.p. 205.7–206.1 °C; ^1^H NMR (600 MHz, Acetone-*d_6_*) *δ* 9.71 (s, 1H), 8.78 (s, 1H), 7.93 (td, *J* = 2.3, 5.2 Hz, 3H), 7.89–7.82 (m, 2H), 7.78 (dd, *J* = 1.5, 8.0 Hz, 1H), 7.75–7.70 (m, 3H), 7.41 (d, *J* = 2.1 Hz, 1H), 7.18 (dd, *J* = 2.1, 8.3 Hz, 1H), 6.92 (d, *J* = 8.3 Hz, 1H) ppm; ^13^C NMR (150 MHz, CD_3_OD) *δ* 169.70, 148.31, 141.14, 135.45, 133.90, 131.96, 131.71, 131.62, 131.15, 130.97, 130.29, 129.53, 128.98, 126.03 (d, *J* = 3.8 Hz), 125.69 (d, *J* = 3.7 Hz), 125.68, 124.87, 124.36, 123.07, 121.43, 119.22, 110.42 ppm. ESI-HRMS, m/z calculated for C_22_H_14_F_3_N_3_O_5_SNa [M + Na]^+^ 512.05011, found 512.04985.

Compound **9b**: Yield: 28.7%, yellow powder; m.p. 205.6–206.1 °C; ^1^H NMR (600 MHz, CD_3_OD) *δ* 7.91 (d, *J* = 7.7 Hz, 1H), 7.84 (d, *J* = 8.9 Hz, 2H), 7.79–7.73 (m, 2H), 7.66 (td, *J* = 6.1, 8.5 Hz, 1H), 7.31 (d, *J* = 2.1 Hz, 1H), 7.10 (ddd, *J* = 2.5, 8.8, 10.9 Hz, 1H), 7.02 (dd, *J* = 2.1, 8.3 Hz, 1H), 6.98 (td, *J* = 2.5, 8.4 Hz, 1H), 6.80 (d, *J* = 8.3 Hz, 1H) ppm; ^13^C NMR (150 MHz, CD_3_OD) *δ* 161.22, 158.60, 157.11, 153.09, 135.71, 131.22, 131.03, 128.45 (d, *J* = 9.6 Hz), 128.08, 127.36 (d, *J* = 28.4 Hz), 127.04, 125.98, 125.52, 122.85 (d, *J* = 4.1 Hz), 122.58 (d, *J* = 4.1 Hz), 122.35, 118.81, 115.64, 110.04 (dd, *J* = 3.3, 19.6 Hz), 109.96, 109.10, 104.41 (t, *J* = 23.1 Hz) ppm; ESI-HRMS, *m*/*z* calculated for C_22_H_13_F_5_N_2_O_3_SNa [M + Na]^+^ 503.04565, found 503.04593.

Compound **9c**: Yield: 21.5%, yellow powder, m.p. 128.1–128.7 °C; ^1^H NMR (600 MHz, CD_3_OD) *δ* 8.01 (d, *J* = 7.6 Hz, 1H), 7.92 (s, 1H), 7.80 (d, *J* = 3.2 Hz, 2H), 7.75 (t, *J* = 7.7 Hz, 1H), 7.48 (d, *J* = 2.1 Hz, 1H), 7.22–7.09 (m, 1H), 7.00–6.80 (m, 1H), 3.00 (q, *J* = 7.4 Hz, 2H), 1.29 (t, *J* = 7.4 Hz, 3H) ppm; ^13^C NMR (150 MHz, CD_3_OD) *δ* 169.80, 140.47, 140.17, 135.59, 135.35, 134.67, 132.18, 132.06, 129.62, 129.21, 126.00 (q, *J* = 3.8 Hz), 125.70 (q, *J* = 4.6 Hz, 125.62, 124.42, 121.64, 117.06, 110.55, 44.74, 7.00 ppm; ESI-HRMS, *m*/*z* calculated for C_18_H_15_F_3_N_2_O_3_SNa [M + Na]^+^ 419.06436, found 419.06477.

Compound **9d**: Yield: 38.4%, orange solid; m.p. 226.5–226.9 °C; ^1^H NMR (600 MHz, CD_3_OCD_3_) *δ* 9.68 (s, 1H), 9.06 (s, 1H), 8.36 (d, *J* = 2.0 Hz, 1H), 8.35 (d, *J* = 2.0 Hz, 1H), 7.95 (d, *J* = 2.0 Hz, 1H), 7.94 (d, *J* = 2.0 Hz, 1H), 7.93 (s, 2H), 7.83 (d, *J* = 7.8 Hz, 1H), 7.76–7.71 (m, 2H), 7.39 (d, *J* = 2.1 Hz, 1H), 7.10 (dd, *J* = 2.2, 8.3 Hz, 1H), 6.90 (d, *J* = 8.3 Hz, 1H) ppm; ^13^C NMR (150 MHz, CD_3_OCD_3_) *δ* 168.30, 168.19, 150.22, 145.13, 141.40, 135.76, 134.76, 132.36, 130.84, 130.63, 130.36, 129.79, 128.99, 128.71, 128.60, 126.00 (d, *J* = 3.8 Hz), 125.70 (d, *J* = 4.6 Hz), 125.99, 124.22, 121.67, 118.52, 110.67 ppm; ESI-HRMS, *m*/*z* calculated for C_22_H_14_F_3_N_3_O_5_SNa [M + Na]^+^ 512.05066, found 512.04985.

Compound **9e**: Yield: 31.6%, yellow amorphous powder; m.p. 151.1–151.5 °C; ^1^H NMR (600 MHz, CDCl_3_) *δ* 8.22 (s, 1H), 7.90 (d, *J* = 7.7 Hz, 1H), 7.85 (d, *J* = 3.5 Hz, 2H), 7.74 (d, *J* = 7.9 Hz, 1H), 7.66 (t, *J* = 7.7 Hz, 1H), 7.42 (d, *J* = 2.2 Hz, 1H), 7.39 (dt, *J* = 2.8, 7.0 Hz, 1H), 7.34–7.30 (m, 1H), 7.27 (d, *J* = 2.0 Hz, 1H), 7.26 (d, *J* = 2.3 Hz, 2H), 7.07 (dd, *J* = 2.1, 8.3 Hz, 1H), 6.87 (d, *J* = 8.3 Hz, 1H), 4.26 (s, 2H) ppm; ^13^C NMR (150 MHz, CDCl_3_) *δ* 169.33, 139.38, 136.55, 135.13, 132.13, 131.13, 130.97, 130.75, 130.75, 129.64, 128.94, 128.79, 128.79, 128.52, 128.36, 126.58 (d, *J* = 2.7 Hz), 126.17 (d, *J* = 4.3 Hz), 124.28, 122.08, 117.25, 110.83, 57.69, 50.89 ppm; ESI-HRMS *m*/*z* calculated for C_23_H_17_F_3_N_2_O_3_SNa [M + Na]^+^ 481.08014, found 481.08042.

Compound **9f**: Yield: 41.3%, yellow powder; m.p. 191.1–191.5 °C; ^1^H NMR (600 MHz, CD_3_OCD_3_) *δ* 9.68 (s, 1H), 8.71 (s, 1H), 7.94 (s, 1H), 7.90 (d, *J* = 7.7 Hz, 1H), 7.86 (d, *J* = 8.0 Hz, 1H), 7.74 (t, *J* = 7.8 Hz, 1H), 7.71 (s, 1H), 7.56 (d, *J* = 2.0 Hz, 1H), 7.55 (d, *J* = 1.8 Hz, 1H), 7.37 (d, *J* = 2.1 Hz, 1H), 7.31 (s, 1H), 7.30 (s, 1H), 7.10 (dd, *J* = 2.1, 8.3 Hz, 1H), 6.88 (d, *J* = 8.3 Hz, 1H), 2.36 (s, 3H) ppm; ^13^C NMR (150 MHz, CD_3_OCD_3_) *δ* 168.33, 143.41, 140.81, 136.81, 135.82, 134.39, 132.24, 131.50, 130.75 (q, *J* = 32.2 Hz), 129.82, 129.43, 129.11, 127.17, 126.16 (q, *J* = 4.0 Hz), 126.05 (q, *J* = 3.9 Hz), 125.54, 125.09, 124.19 (q, *J* = 271.7 Hz), 123.29, 121.39, 117.97, 110.46, 20.51 ppm; ESI-HRMS, *m*/*z* calculated for C_23_H_17_F_3_N_2_O_3_SNa [M + Na]^+^ 481.08042, found 481.08042.

Compound **9g**: Yield: 41.2%, yellow powder; m.p. 258.4–259.3 °C; ^1^H NMR (600 MHz, DMSO-*d_6_*) *δ* 10.62 (d, *J* = 24.1 Hz, 1H), 10.02–9.79 (m, 1H), 8.89 (s, 1H), 8.55 (d, *J* = 7.9 Hz, 1H), 7.90–7.76 (m, 2H), 7.70 (dt, *J* = 7.9, 15.6 Hz, 1H), 7.66–7.58 (m, 2H), 7.55–7.42 (m, 1H), 7.31 (dd, *J* = 7.9, 34.4 Hz, 2H), 6.95–6.77 (m, 1H), 6.72 (dd, *J* = 8.3, 40.3 Hz, 1H), 2.32 (d, *J* = 13.5 Hz, 3H) ppm; ^13^C NMR (150 MHz, DMSO-*d*_6_) *δ* 167.42, 143.48, 139.02, 137.14, 136.13, 135.78, 135.04, 130.86 (q, *J* = 276.33 Hz), 130.07, 130.07, 129.96, 129.65, 128.72, 128.66 (d, *J* = 3.7 Hz), 127.29, 127.29, 127.15, 126.99, 125.37, 124.69, 115.88, 110.33, 21.42 ppm; ESI-HRMS *m*/*z* calculated for C_23_H_17_F_3_N_2_O_3_SNa [M + Na]^+^ 481.08020, found 481.08042.

### 3.4. General Procedure for the Synthesis of ***9h***-***9i***

5-Aminoindolin-2-one (**6a**) (1.0 mmol) and piperidine were added to a solution of the suitably substituted aromatic aldehyde (1.0 mmol) in methyl alcohol under nitrogen protection. The reaction mixture was refluxed for 24 h while tracking the reaction progress by TLC. Then, the reaction mixture was concentrated, and the resultant crude solid product was purified by silica gel column chromatography and a Sephadex LH-20 column (CH_3_OH) to afford target compounds **9i**-**9h.**

Compound **9h**: Yield: 40.3%, grayish-white powder, m.p. 166.3–166.8 °C; ^1^H NMR (600 MHz, CD_3_OD) *δ* 8.69 (s, 1H), 8.25 (s, 1H), 8.16 (d, *J* = 7.8 Hz, 1H), 7.80 (d, *J* = 7.5 Hz, 1H), 7.71 (t, *J* = 7.8 Hz, 1H), 7.33 (d, *J* = 2.2 Hz, 1H), 7.26 (dd, *J* = 8.2, 2.2 Hz, 1H), 6.95 (d, *J* = 8.2 Hz, 1H), 6.79–6.61 (m, 1H). 3.34 (p, *J* = 1.7 Hz, 2H) ppm; ^13^C NMR (150 MHz, CD_3_OD) *δ* 178.52, 157.13, 145.60, 142.40, 137.31, 131.85, 129.39, 128.30, 127.09 (d, J = 3.9 Hz), 126.85, 124.51, 123.81, 121.26, 117.40, 117.38, 113.73 (d, *J* = 235.8 Hz), 109.74 ppm; ESI-HRMS *m*/*z* calculated for C_16_H_11_F_3_N_2_ONa [M + Na]^+^ 327.07193, found 327.07157.

Compound **9i**: Yield: 41.3%, yellow amorphous powder; m.p. 191.1–191.5 °C; ^1^H NMR (600 MHz, DMSO-*d_6_*) *δ* 10.49 (s, 1H), 8.76 (s, 1H), 8.42 (s, 2H), 8.09 (s, 1H), 7.23 (d, *J* = 3.23 Hz, 2H), 6.84 (s, 1H) ppm; ^13^C NMR (150 MHz, DMSO-*d*_6_) *δ* 176.87, 154.87, 144.05, 143.71, 139.10, 131.20 (q, *J* = 33.5 Hz), 128.59, 128.59, 127.33, 124.48, 124.01, 123.46 (q, *J* = 272.8 Hz), 122.47, 117.95, 109.88, 36.28 ppm. ESI-HRMS *m*/*z* calculated for C_17_H_10_F_6_N_2_ONa [M + Na]^+^ 395.05930, found 395.05895.

### 3.5. Cytotoxicity Assay

The RAW 264.7 murine macrophage cells were obtained from the Cell Bank of the Chinese Academy of Sciences (Shanghai, China). RAW 264.7 cells were cultured in DMEM (Dulbecco’s Modified Eagle Medium) medium supplemented with 10% fetal bovine serum and 1% penicillin/streptomycin at 37 °C and 5% CO_2_ in a saturated humidified incubator. The Cell Counting Kit-8 (CCK-8) method was used to evaluate the cytotoxicity of all the compounds on RAW 264.7 cells in vitro. The cells (5 × 10^4^ cells/well) were seeded in 96-well culture plates and treated with test compounds (6.25, 12.5, 25, 50, and 100 μg/mL). After 24 h of cultivation, the cells were incubated with a CCK-8 solution (10 µL). After incubation at 37 °C and 5% CO_2_ for 2–4 h, the optical density (OD) was recorded at 450 nm by a microplate reader. The half-maximal inhibitory concentration (IC_50_) was calculated by SPSS 22.0 software. PDTC was used as a positive control.

### 3.6. Determination of Anti-Inflammatory Activity

RAW 264.7 cells were cultured in DMEM medium supplemented with 10% fetal bovine serum and 1% penicillin/streptomycin at 37 °C and 5% CO_2_ in a saturated humidified incubator. Then cells were seeded in 24-well plates (4 × 10^5^ cells/well) after 24 h of incubation and treated with different concentrations of compounds (80, 40, 20, and 5 μM) for 2 h. The cells were stimulated with LPS (5 μg/mL) for 24 h, the supernatant was transferred to a new 24-well microtiter plate, and the NO concentration in the cell supernatant was calculated using an NO detection kit (Beyotime Company, Shanghai, China).

### 3.7. In Vitro COX-2 Inhibitory Assay

The in vitro COX-2 inhibitory activity of compounds **4a**-**4w** and **9a**-**9g** was evaluated using a Cyclooxygenase-2 Inhibitor Screening Kit (Beyotime Company, Shanghai, China) [41]. Briefly, human recombinant COX-2 enzyme, COX-2 cofactor, and COX-2 assay buffer, were preincubated with test compounds for 10 min at 37 °C, and then the COX-2 probe was added. The reaction was started by the addition of COX-2 substrate and allowed to proceed for 5 min. The intensity of fluorescence at an emission wavelength of 590 nm upon 560 nm excitation was measured using a microplate reader (Thermo Varioskan LUX, Waltham, MA, USA). All tests were repeated three times independently.

### 3.8. Molecular Docking

A docking study was performed as described previously [42,43]. For receptor preparation, the crystal structure of COX-2 (PDB code: 3LN1) was downloaded from the Protein Data Bank (https://www.rcsb.org (accessed on 16 April 2023)). The ligand docked into the 3LN1 structure by utilizing AutoDock Vina 1.1.2. The binding affinity, the value of the dissociation constant in units of molarity, was used to evaluate the interaction between the ligands and the protein. For the initial screening of the compounds, ten poses were generated for docking, and then an estimated range of binding affinities was calculated and used for secondary screening. The more negative the Vina docking score is, the higher the binding affinity, and the compound with the lower energy score is taken as the subsequent analysis object. Hydrogen atoms were added with their standard geometry, and the crystallographic water molecules in 3LN1 were removed. At neutral pH, all the dissociable residues in the system were set to their protonated states. Celecoxib, an inhibitor of COX-2, was used as a reference compound to define the active site of 3LN1. For further docking, the edited COX-2 and ligand files were transcribed into PDBQT format. All of the default parameters were used.

## 4. Conclusions

In our study, thirty-three oxindole analogues bearing α, β-unsaturated ketones were designed and synthesized via the Knoevenagel condensation reaction. Fifteen compounds were new. The anti-inflammatory properties and COX-2 inhibitory activities of all the compounds were evaluated for the first time in vitro. Compounds **4a**, **4e**, **4i**, **4j**, and **9d** showed different degrees of inhibition against NO production in LPS-stimulated RAW 264. Seven cells and three new compounds (**4e**, **9h**, and **9i**) exhibited significant COX-2 inhibitory activities. The results of the three compounds suggested that they had the potential to be COX-2 inhibitors, and compound **4e** had the potential to be an anti-inflammatory lead compound for further optimization and evaluation. The possible mechanisms by which COX-2 recognized **4e**, **9h**, and **9i** were predicted by molecular docking. These findings are promising for the discovery of new drugs that inhibit COX-2 and inflammation, and this study provides more diverse chemical entities for the research and development of innovative anti-inflammatory drugs.

## Figures and Tables

**Table 1:** Inhibition of NO production by seven compounds in LPS-activated RAW 264.7 cells (IC_50_ values) ($\bar{x}$ ± SD, *n* = 3).

Compd.	IC_50_ (μM) ^b^
**4a**	17.81 ± 1.86
**4e**	13.51 ± 0.48
**4i**	20.41 ± 1.61
**4j**	16.31 ± 0.35
**9d**	10.03 ± 0.27
**9h**	-
**9i**	-
PDTC ^a^	13.71 ± 0.88

^a^ Ammonium pyrrolidinedithiocarbamate (PDTC) was used as the positive control. ^b^ The other derivatives had low inhibition against NO production activity and are not listed in Table 2.

**Table 2:** IC_50_ values of six derivatives of COX-2 inhibitory activity in vitro ($\bar{x}$ ± SD, *n* = 3).

Compd.	IC_50_ (μM)
**4a**	19.90 ± 4.76
**4e**	3.34 ± 0.05
**9h**	2.35 ± 0.04
**9i**	2.42 ± 0.10
**4k**	>20
**9f**	>20
Celecoxib *	0.03 ± 0.97

* Celecoxib was used as the positive control.

**Table 3:** The docking results of compounds **9k**, **9i**, and **4k** with COX-2.

The Resulting Docking Scores (Binding Energy)			
Pose	4e	9i	9h
1	−9.8	−9.3	−8.7
2	−8.9	−9.1	−7.6
3	−8.8	−9.1	−7.5
4	−8.2	−8.9	−7.0
5	−8.1	−8.5	−6.9
6	−8.0	−8.2	−6.4
7	−7.8	−8.1	−6.4
8	−6.9	−8.0	−6.3
9	−6.3	−7.8	−5.2
10	−5.8	−6.4	−4.7

## Data Availability

The data presented in this study are available in the Supplementary Materials.

## Supplementary Materials

The following supporting information can be downloaded at: https://www.mdpi.com/article/10.3390/molecules28124668/s1, Figures S1-1–S33-3: ^1^H-NMR, ^13^C-NMR, ESI- HRMS spectra for compounds **4a**-**4x** and **9a**-**9i.**

## References

[1] Gao Y., Duan Y.Z. (2010). Increased COX-2 in the trigeminal nucleus caudalis is involved in orofacial pain induced by experimental tooth movement. Anat. Rec..

[2] Kumar A., Kour G., Chibber P., Saroch D., Kumar C., Ahmed Z. (2022). Novel alantolactone derivative AL-04 exhibits potential anti-inflammatory activity via modulation of iNOS, COX-2 and NF-kappa B. Cytokine.

[3] Abdelall E.K.A., Lamie P.F., Ali W.A.M. (2016). Cyclooxygenase-2 and 15-lipoxygenase inhibition, synthesis, anti-inflammatory activity and ulcer liability of new celecoxib analogues: Determination of region-specific pyrazole ring formation by NOESY. Bioorg. Med. Chem. Lett..

[4] Mishra C.B., Kumari S., Prakash A., Yadav R., Tiwari A.K., Pandey P., Tiwari M. (2018). Discovery of novel methylsulfonyl phenyl derivatives as potent human Cyclooxygenase-2 inhibitors with effective anticonvulsant action: Design, synthesis, in-silico, in vitro and in vivo evaluation. Eur. J. Med. Chem..

[5] Zarghi A., Najafnia L., Daraee B., Dadrass O.G., Hedayati M. (2007). Synthesis of 2, 3-diaryl-1,3-thiazolidine-4-one derivatives as selective cyclooxygenase (COX-2) inhibitors. Bioorg. Med. Chem. Lett..

[6] Zebardast T., Zarghi A., Daraie B., Hedayati M., Dadrass O.G. (2009). Design and synthesis of 3-alkyl-2-aryl-1,3-thiazinan-4-one derivatives as selective cyclooxygenase (COX-2) inhibitors. Bioorg. Med. Chem. Lett..

[7] Laine L., White W.B., Rostom A., Hochberg M. (2008). COX-2 selective inhibitors in the treatment of osteoarthritis. Semin. Arthritis Rheum..

[8] Dionne R.A., Berthold C.W. (2001). Therapeutic uses of non-steroidal anti-inflammatory drugs in dentistry. Crit. Rev. Oral Biol. Med..

[9] Huber M.A., Terezhalmy G.T. (2006). The use of COX-2 inhibitors for acute dental pain: A second look. J. Am. Dent. Assoc..

[10] Nekoofar M.H., Sadeghipanah M., Dehpour A.R. (2003). Evaluation of meloxicam (A cox-2 inhibitor) for management of postoperative endodontic pain: A double-blind placebo-controlled study. J. Endod..

[11] Gupta A., Bah M. (2016). NSAIDs in the treatment of postoperative pain. Curr. Pain Headache Rep..

[12] Al-Hourani B.J., Sharma S.K., Mane J.Y., Tuszynski J., Baracos V., Kniess T., Suresh M., Pietzsch J., Wuest F. (2011). Synthesis and evaluation of 1,5-diaryl-substituted tetrazoles as novel selective cyclooxygenase-2 (COX-2) inhibitors. Bioorg. Med. Chem. Lett..

[13] Abdellatif K.R., Fadaly W.A., Ali W.A., Kamel G.M. (2016). Synthesis, cyclooxygenase inhibition, anti-inflammatory evaluation and ulcerogenic liability of new 1,5-diarylpyrazole derivatives. J. Enzym. Inhib. Med. Chem..

[14] Dhanjal J.K., Sreenidhi A.K., Bafna K., Katiyar S.P., Goyal S., Grover A., Sundar D. (2015). Computational structure-based de novo design of hypothetical inhibitors against the anti-inflammatory target COX-2. PLoS ONE.

[15] Grosser T., Ricciotti E., FitzGerald G.A. (2017). The Cardiovascular pharmacology of nonsteroidal anti-inflammatory drugs. Trends Pharmacol. Sci..

[16] Elshemy H.A.H., Abdelall E.K.A., Azouz A.A., Moawad A., Ali W.A.M., Safwat N.M. (2017). Synthesis, anti-inflammatory, cyclooxygenases inhibitions assays and histopathological study of poly-substituted 1,3,5-triazines: Confirmation of regiospecific pyrazole cyclization by HMBC. Eur. J. Med. Chem..

[17] Patrono C., Baigent C. (2017). Coxibs, Traditional NSAIDs, and cardiovascular safety post-precision: What we thought we knew then and what we think we know now. Clin. Pharmacol. Ther..

[18] Motika S.E., Hergenrother P.J. (2020). Re-engineering natural products to engage new biological targets. Nat. Prod. Rep..

[19] Guthikonda R.N., Cama L.D., Quesada M., Woods M.F., Salzmann T.N., Christensen B.G. (1987). Modification of natural products to improve their biological properties. Pure Appl. Chem..

[20] Zongru G. (2017). The modification of natural products for medical use. Acta Pharm. Sin. B.

[21] Viegas J.C., Danuello A., da Silva Bolzani V., Barreiro E.J., Fraga C.A. (2007). Molecular hybridization: A useful tool in the design of new drug prototypes. Curr. Med. Chem..

[22] Giselle C., Costa F. (2006). Oxindoles and copper complexes with oxindole-derivatives as potential pharmacological agents. J. Braz. Chem. Soc..

[23] Khetmalis Y.M., Shivani M., Murugesan S., Chandra Sekhar K.V.G. (2021). Oxindole and its derivatives: A review on recent progress in biological activities. Biomed. Pharmacother..

[24] Kaur M., Singh M., Chadha N., Silakari O. (2016). Oxindole: A chemical prism carrying plethora of therapeutic benefits. Eur. J. Med. Chem..

[25] Dey P., Kundu A., Han S.H., Kim K.S., Park J.H., Yoon S., Kim I.S., Kim H.S. (2020). Biological evaluation of exindole derivative as a novel anticancer agent against human kidney carcinoma cells. Biomolecules.

[26] Khan M., Yousaf M., Wadood A., Junaid M., Ashraf M., Alam U., Ali M., Arshad M., Hussain Z., Khan K.M. (2014). Discovery of novel oxindole derivatives as potent alpha-glucosidase inhibitors. Bioorg. Med. Chem..

[27] Dreifuss A.A., Bastos-Pereira A.L., Avila T.V., Soley B.D.S., Rivero A.J., Aguilar J.L., Acco A. (2010). Antitumoral and antioxidant effects of a hydroalcoholic extract of cat’s claw (Uncaria tomentosa) (Willd. Ex Roem. & Schult) in an in vivo carcinosarcoma model. J. Ethnopharmacol..

[28] Chaudhari P., Bari S., Surana S., Shirkhedkar A., Wakode S., Shelar S., Racharla S., Ugale V., Ghodke M. (2022). Logical synthetic strategies and structure-activity relationship of indolin-2-one hybrids as small molecule anticancer agents: An overview. J. Mol. Struct..

[29] Xu Y., Zhang X.J., Li W.B., Wang X.R., Wang S., Qiao X.P., Chen S.W. (2020). Design, synthesis and biological evaluation of indole-2-one derivatives as potent BRD4 inhibitors. Eur. J. Med. Chem..

[30] Tseng C.C., Baillie G., Donvito G., Mustafa M.A., Juola S.E., Zanato C., Massarenti C., Dall’Angelo S., Harrison W.T.A., Lichtman A.H. (2019). The trifluoromethyl group as a bioisosteric replacement of the aliphatic nitro group in CB (1) receptor positive allosteric modulators. J. Med. Chem..

[31] Egami H., Sodeoka M. (2014). Trifluoromethylation of alkenes with concomitant introduction of additional functional groups. Angew. Chem. Int. Ed. Engl..

[32] Feng S., Li C., Chen D., Zheng X., Yun H., Gao L., Shen H.C. (2017). Discovery of methylsulfonyl indazoles as potent and orally active respiratory syncytial Virus (RSV) fusion inhibitors. Eur. J. Med. Chem..

[33] Khurshid A., Saeed A., Erben M.F., Hökelek T., Jabeen E. (2023). DFT guided substitution effect on azomethine reactive center in newly synthesized Schiff base aromatic scaffolds; syntheses, characterization, single crystal XRD, Hirshfeld surface and crystal void analyses. J. Mol. Struct..

[34] Sondhi S.M., Singh N., Kumar A., Lozach O., Meijer L. (2006). Synthesis, anti-inflammatory, analgesic and kinase (CDK-1, CDK-5 and GSK-3) inhibition activity evaluation of benzimidazole/benzoxazole derivatives and some Schiff’s bases. Bioorg. Med. Chem..

[35] Vijesh A.M., Isloor A.M., Shetty P., Sundershan S., Fun H.K. (2013). New pyrazole derivatives containing 1,2,4-triazoles and benzoxazoles as potent antimicrobial and analgesic agents. Eur. J. Med. Chem..

[36] Wei D., Ning L.I., Gui L.U., Yao K. (2006). Synthesis, catalytic and biological activity of novel dinuclear copper complex with Schiff base. Sci. China Ser. B.

[37] John W.C. (2001). Nitric oxide in immunity and inflammation. Int. Immunopharmacol..

[38] Bogdan C. (2001). Nitric oxide and the immune response. Nat. Immunol..

[39] Sungeun A., Muhammad H.S., Noh H.Y., Kim Y.J., Jin C.G., Yang C.D. (2015). Anti-inflammatory activity of ginsenosides in LPS-stimulated RAW 264.7 cells. Sci. Bull..

[40] Futagami A., Ishizaki M., Fukuda Y., Seiji K., Nobuaki Y. (2002). Wound healing involves induction of Cyclooxygenase-2 expression in rat skin. Lab. Investig..

[41] Yan Y.M., Zhang H.X., Liu H., Wang Y., Wu J.B., Li Y.P., Cheng Y.X. (2019). (+/−)-Lucidumone, a COX-2 inhibitory ccaged fungal meroterpenoid from *Ganoderma lucidum*. Org. Lett..

[42] Orlando B.J., Malkowski M.G. (2016). Substrate-selective inhibition of Cyclooxygeanse-2 by fenamic acid derivatives is dependent on peroxide tone. J. Biol. Chem..

[43] Qin F.Y., Zhang H.X., Di Q.Q., Wang Y., Yan Y.M., Chen W.L., Cheng Y.X. (2020). Ganoderma cochlear metabolites as probes to identify a COX-2 active site and as in vitro and in vivo anti-inflammatory agents. Org. Lett..

