# Supplementary material for: Design, Synthesis, and Evaluation of the COX-2 Inhibitory Activities of New 1,3-Dihydro-2H-indolin-2-one Derivatives
Source: Molecules. 2023 Jun 9;28(12):4668. doi: 10.3390/molecules28124668 (PMC10305287; doi:10.3390/molecules28124668)
Supplement: Supplementary file 1 [file molecules-28-04668-s001.zip › molecules-2380734-supplementary.pdf]

Supplementary information

Design, Synthesis and Evaluation of the COX-2 Inhibitory Activities of New 1, 3-dihydro-*2H*-indolin-2-one Derivatives

Table of contents Page No.

(*Z*)-1-acetyl-6-(trifluoromethyl)-3-(3-(trifluoromethyl)benzylidene)indolin-2-one (4a) 3

(*E*)-1-acetyl-6-(trifluoromethyl)-3-(3-(trifluoromethyl)benzylidene)indolin-2-one (4b) 5

1-Acetyl-5-chloro-3-(3-(trifluoromethyl)benzylidene)indolin-2-one(4c) 7

1-Acetyl-3-(3, 5-bis(trifluoromethyl)benzylidene)-6-chloroindolin-2-one (4d) 9

(*E*)1-acetyl-5-amino-3-(3-(trifluoromethyl)benzylidene)indolin-2-one (4e) 11

(Z)-1-acetyl-3-(3,5-bis(trifluoromethyl)benzylidene)-6-chloroindolin-2-one(4f) 13

1-Acetyl-3-benzylideneindolin-2-one (4g) 15

1-Acetyl-6-chloro-3-(3-(trifluoromethyl)benzylidene)indolin-2-one(4h) 17

1-Acetyl-6-chloro-3-(4-(trifluoromethyl)benzylidene)indolin-2-one(4i) 19

1-Acetyl-3-(3-(trifluoromethyl)benzylidene)indolin-2-one(4j) 21

1-Acetyl-6-chloro-3-(2-(trifluoromethyl)benzylidene)indolin-2-one(4k) 23

1-Acetyl-6-chloro-3-(4-chloro-3-(trifluoromethyl)benzylidene)indolin-2-one(4l) 25

1-Acetyl-6-chloro-3-(4-chloro-3-fluorobenzylidene)indolin-2-one(4m) 27

1-Acetyl-6-chloro-3-(2, 3-difluorobenzylidene)indolin-2-one(4n) 29

1-Acetyl-5-chloro-3-(3-chloro-2-fluorobenzylidene)indolin-2-one(4o)........................31
1-Acetyl-3-(3-chloro-2-fluorobenzylidene)-6-(trifluoromethyl)indolin-2-one (4p).....33
1-Acetyl-6-chloro-3-(3-chloro-2-fluorobenzylidene)indolin-2-one(4q)........................35
1-Acetyl-6-chloro-3-(3-methoxy-5-(trifluoromethyl)benzylidene)indolin-2-one(4r)..37
1-Acetyl-6-chloro-3-(3-methoxybenzylidene)indolin-2-one(4s) 39
Tert-buty-6-chloro-2-oxo-3-(3-(trifluoromethyl)benzylidene)indoline-1-carboxylate(4t) 41
1-Acetyl-6-chloro-3-(3-(trifluoromethoxy)benzylidene)indolin-2-one(4u) 43
1-Acetyl-6-chloro-3-(pyridin-4-ylmethylene)indolin-2-one(4v) 45
1-Acetyl-6-chloro-3-((2-(trifluoromethyl)pyridin-4-yl)methylene)indolin-2-one(4w)47
Tert-butyl -4-((1-acetyl-6-chloro-2-oxoindolin-3-ylidene)methyl)benzoate(4x)...........49
nitro-N-(2-oxo-3-(3-(trifluoromethyl)benzylidene)indolin-5-yl)benzenesulfonamide(9a) 53
2,4-Difluoro-*N*-(2-oxo-3-(3-(trifluoromethyl)benzylidene)indolin-5-yl)benzenesulfon amide (9b)...55
N-(2-oxo-3-(3-(trifluoromethyl)benzylidene)indolin-5-yl)ethanesulfonamide (9c)....57
4-Nitro-N-(2-oxo-3-(3-(trifluoromethyl)benzylidene)indolin-5-yl)benzenesulfonamid e (9d)...51
N-(2-oxo-3-(3-(trifluoromethyl)benzylidene)indolin-5-yl)-1-phenylmethanesulfonam ide (9e) 59
(Z)-4-methyl-N-(2-oxo-3-(3-(trifluoromethyl)benzylidene)indolin-5-yl)benzenesulfon amide (9f) 61
(*E*)-4-methyl-N-(2-oxo-3-(3-(trifluoromethyl)benzylidene)indolin-5-yl)benz-enesulfo namide (9g). 63
5-((3,5-bis(trifluoromethyl)benzylidene)amino)indolin-2-one (9i)................................65
5-((3,5-bis(trifluoromethyl)benzylidene)amino)indolin-2-one (9i)................................67

Relevant spectroscopic data (¹H, ¹³C and HRMS Spectra)

(*Z*)-1-acetyl-6-(trifluoromethyl)-3-(3-(trifluoromethyl)benzylidene)indolin-2-one (4a)

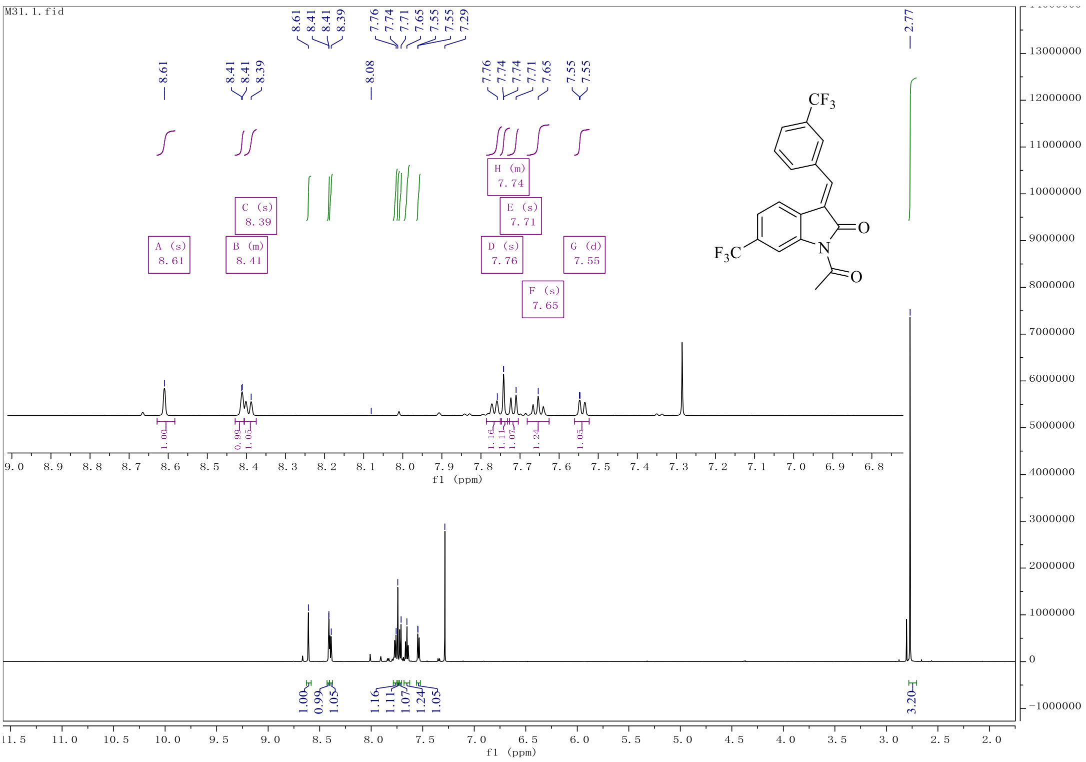

Figure S1-1. ¹H NMR spectrum (600 Hz, $CDCl_3$) of compound **4a**

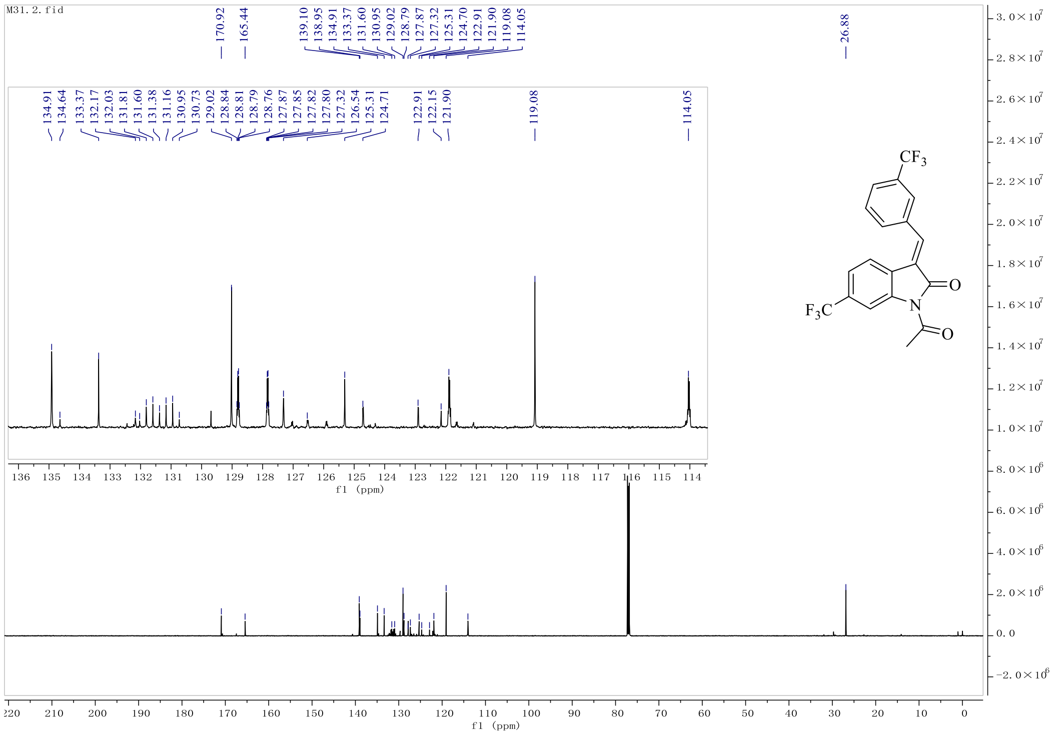

Figure S1-2. ¹³C NMR spectrum (150 Hz, $CDCl_3$) of compound **4a**

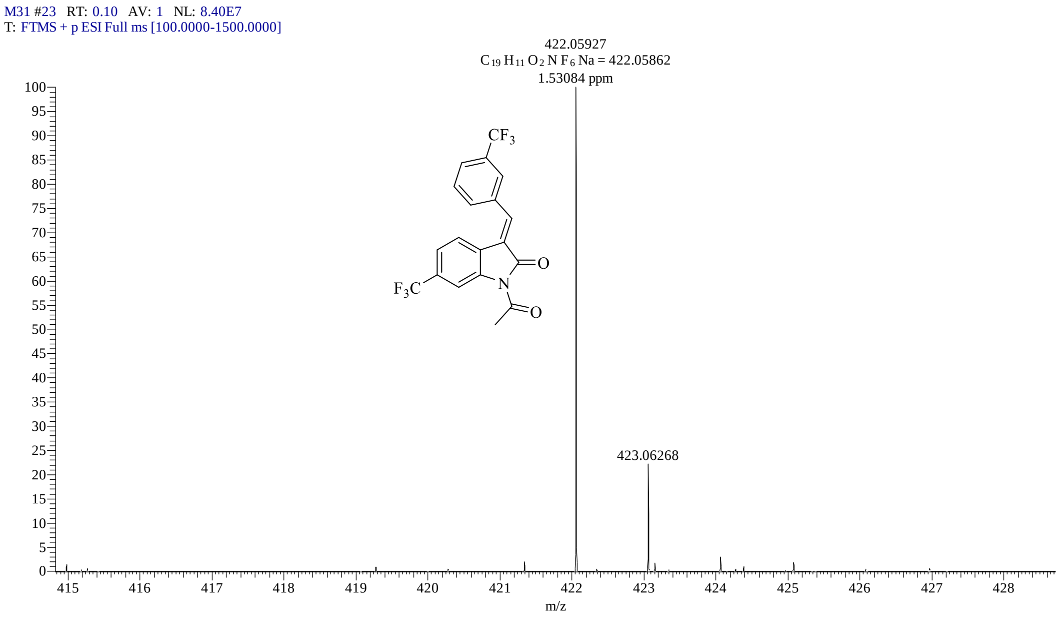

Figure S1-3. HRMS of compound **4a**

(*E*)-1-acetyl-6-(trifluoromethyl)-3-(3-(trifluoromethyl)benzylidene)indolin-2-one (4b)

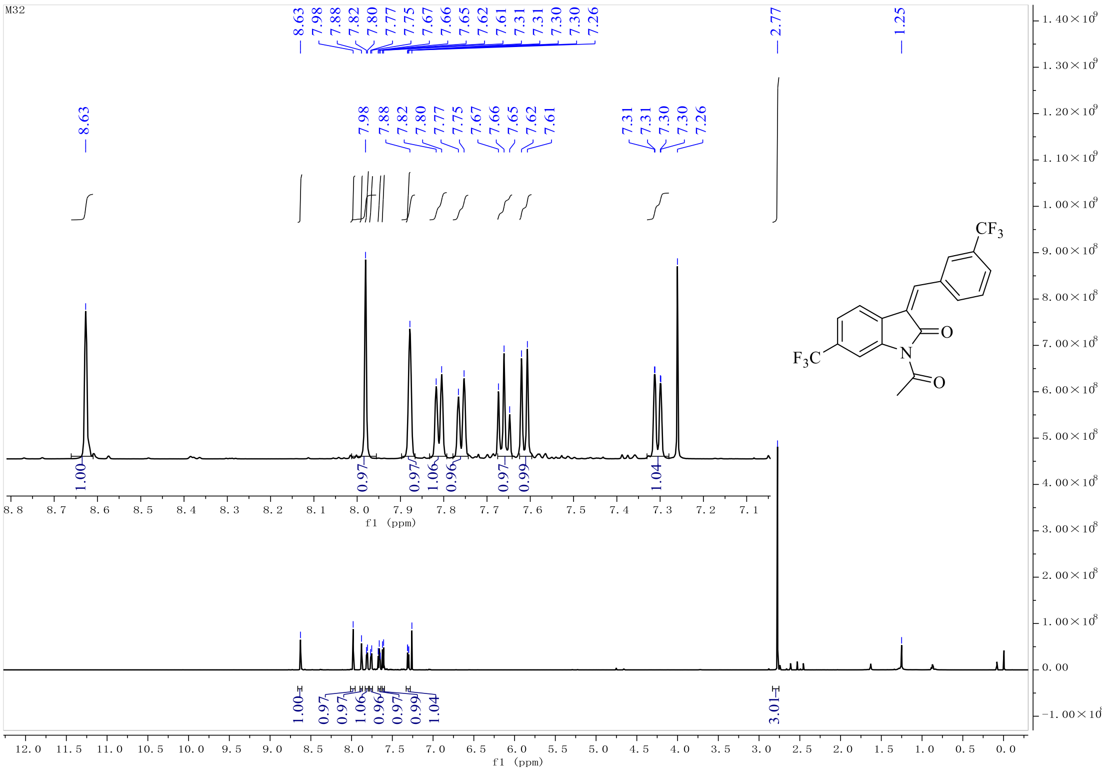

Figure S2-1. ^{1}H NMR spectrum (600 Hz, CDCl3) of compound **4b**

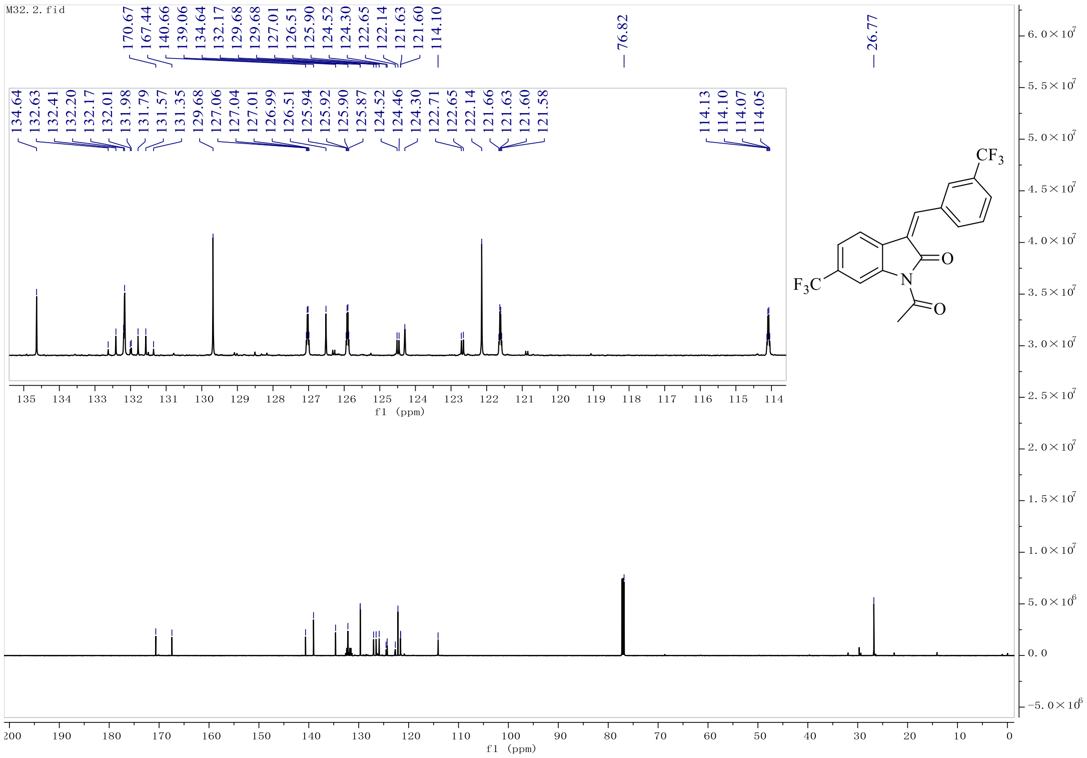

Figure S2-2. ^{13}C NMR spectrum (150 Hz, CDCl3) of compound **4b**

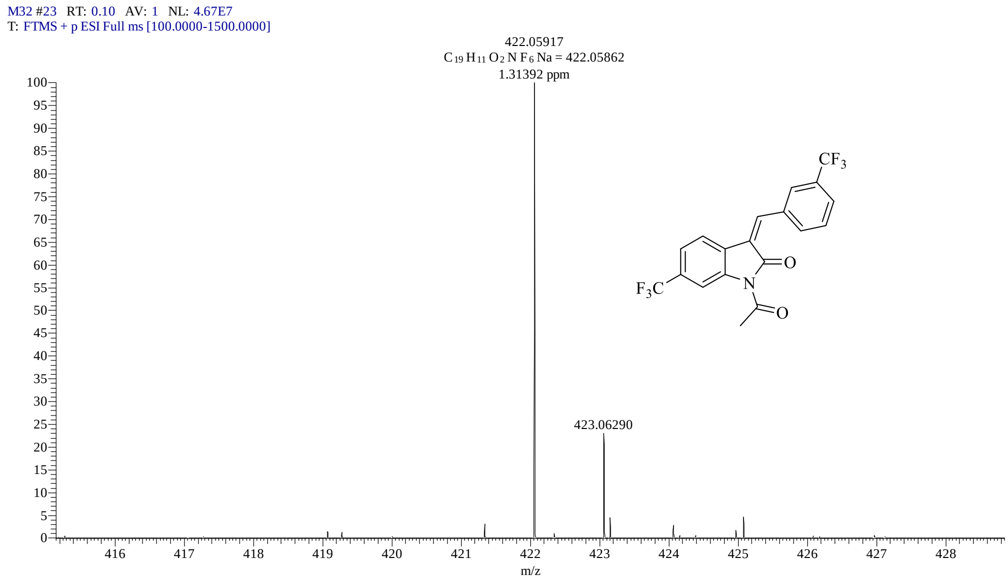

Figure S2-3. HRMS of compound **4b**

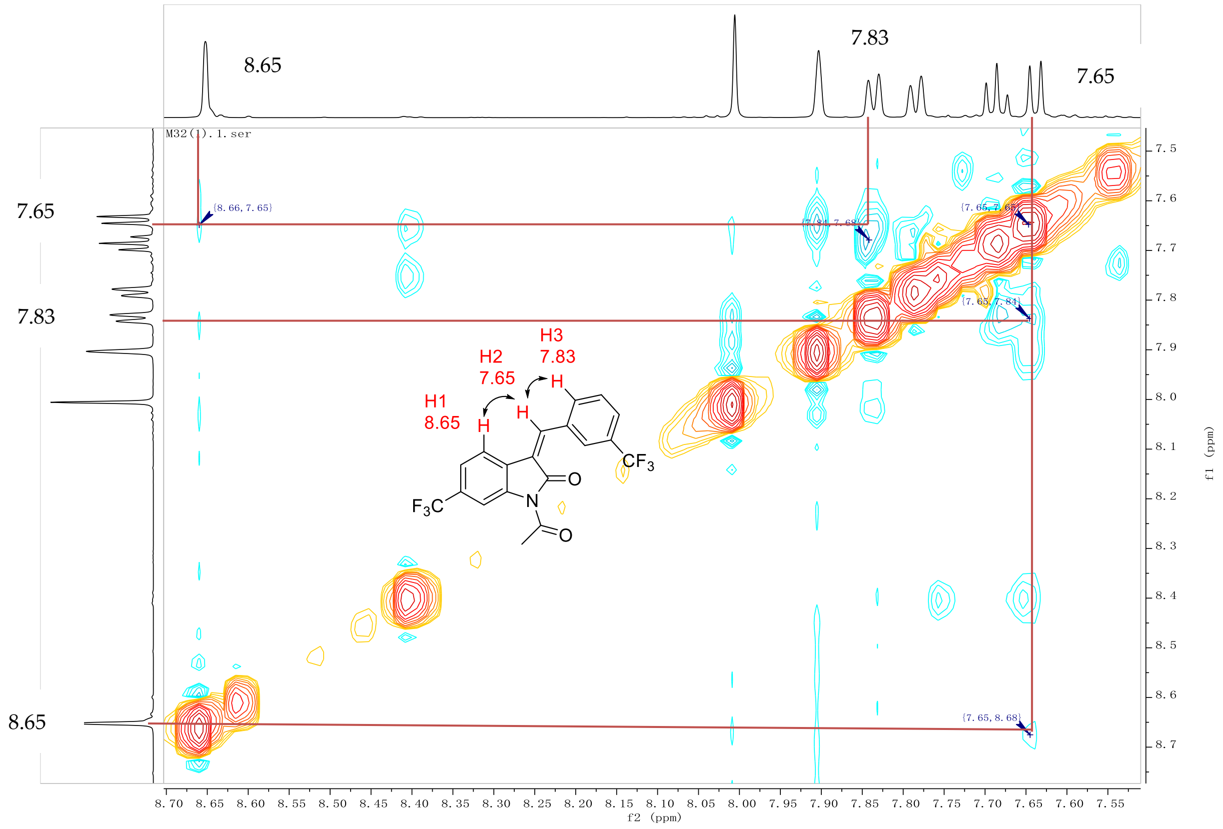

Figure S3-4. 2D-NOEY spectrum (600 Hz, $CDCl_3$) of compound **4b**

1-Acetyl-5-chloro-3-(3-(trifluoromethyl)benzylidene)indolin-2-one(4c)

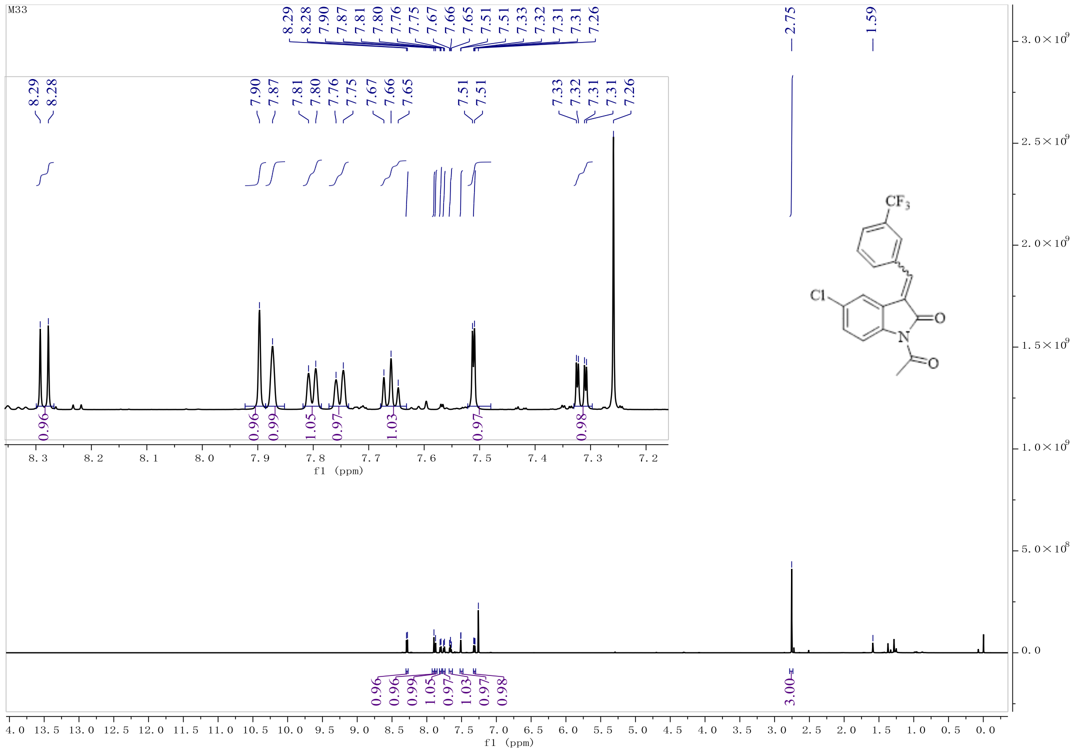

Figure S3-1. ¹H, NMR spectrum (600 Hz, CDCl₃) of compound **4c**

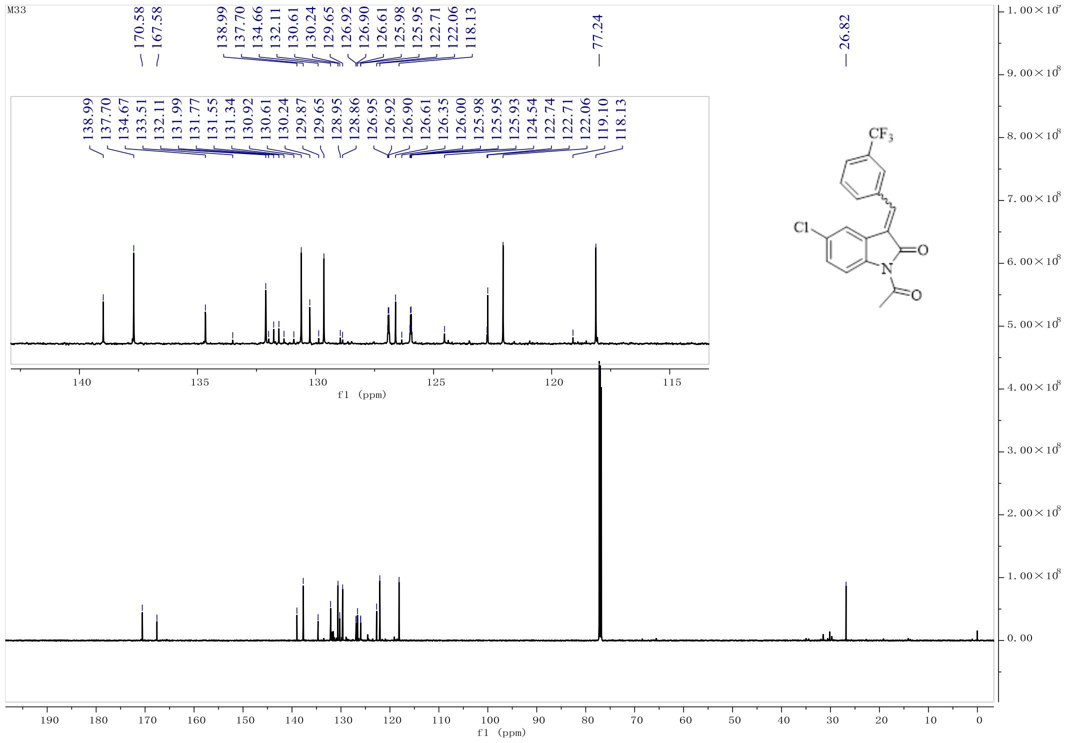

Figure S3-2. ¹³C NMR spectrum (150 Hz, CDCl₃) of compound **4c**

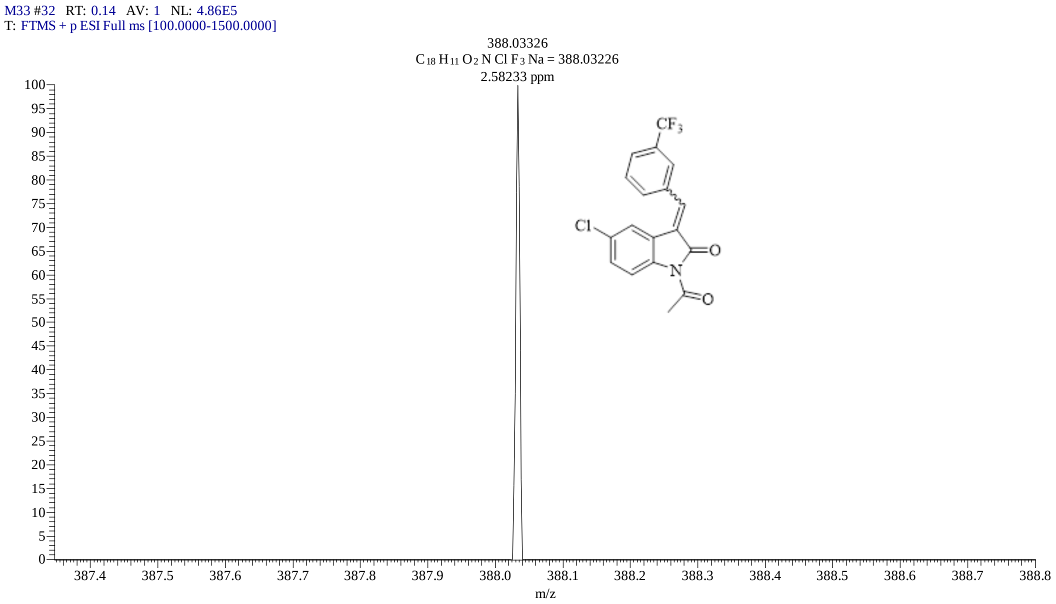

Figure S3-3. HRMS of compound **4c**

1-Acetyl-3-(3, 5-bis(trifluoromethyl)benzylidene)-6-chloroindolin-2-one (4d)

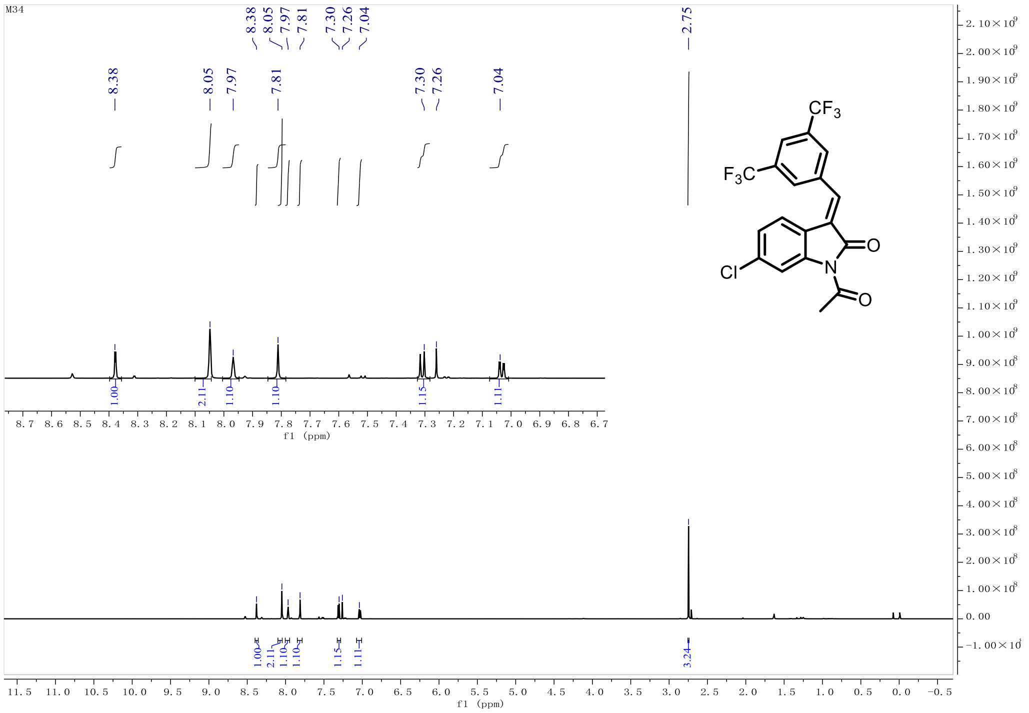

Figure S4-1. ^{1}H, NMR spectrum (600 Hz, $CDCl_3$) of compound **4d**

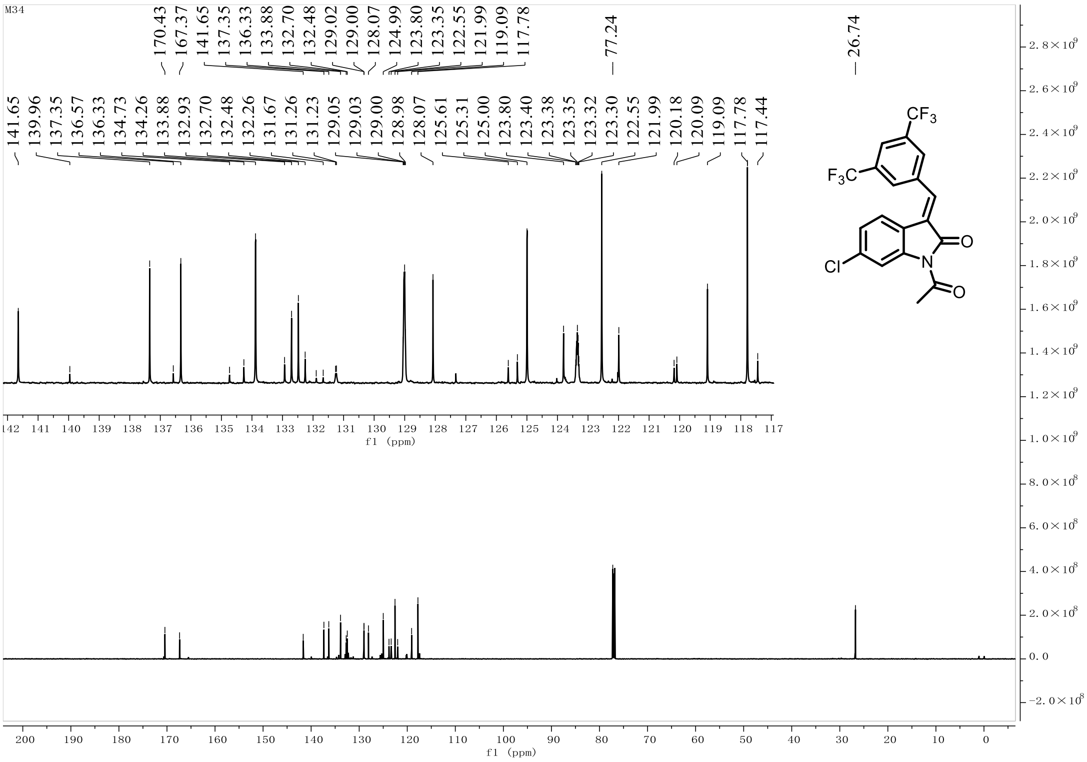

Figure S4-2. ^{13}C NMR spectrum (150 Hz, $CDCl_3$) of compound **4d**

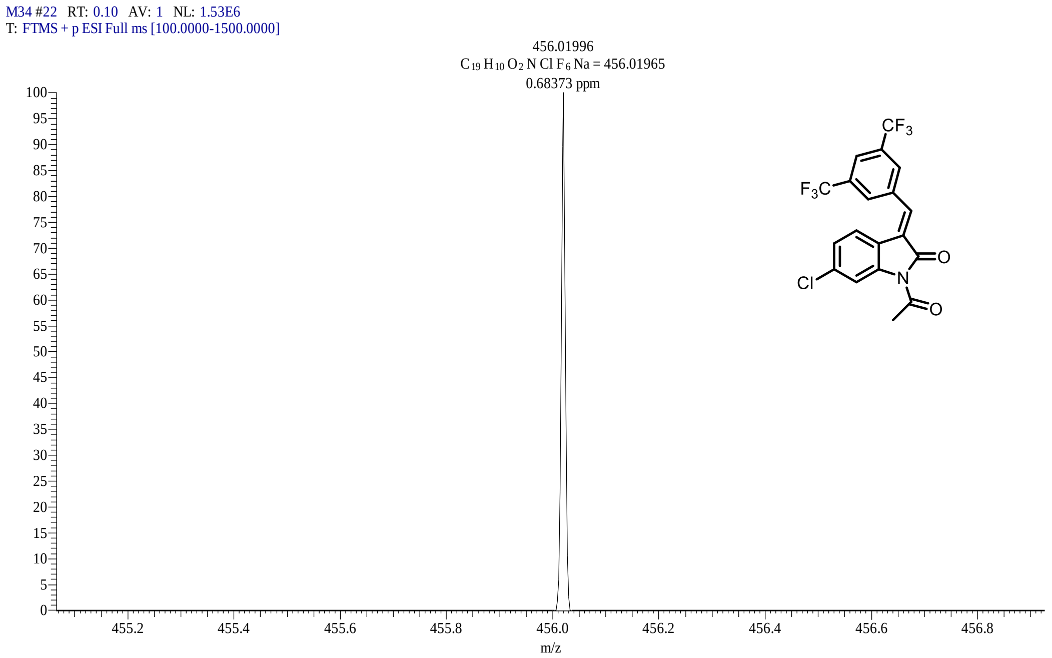

Figure S4-3. HRMS of compound **4d**

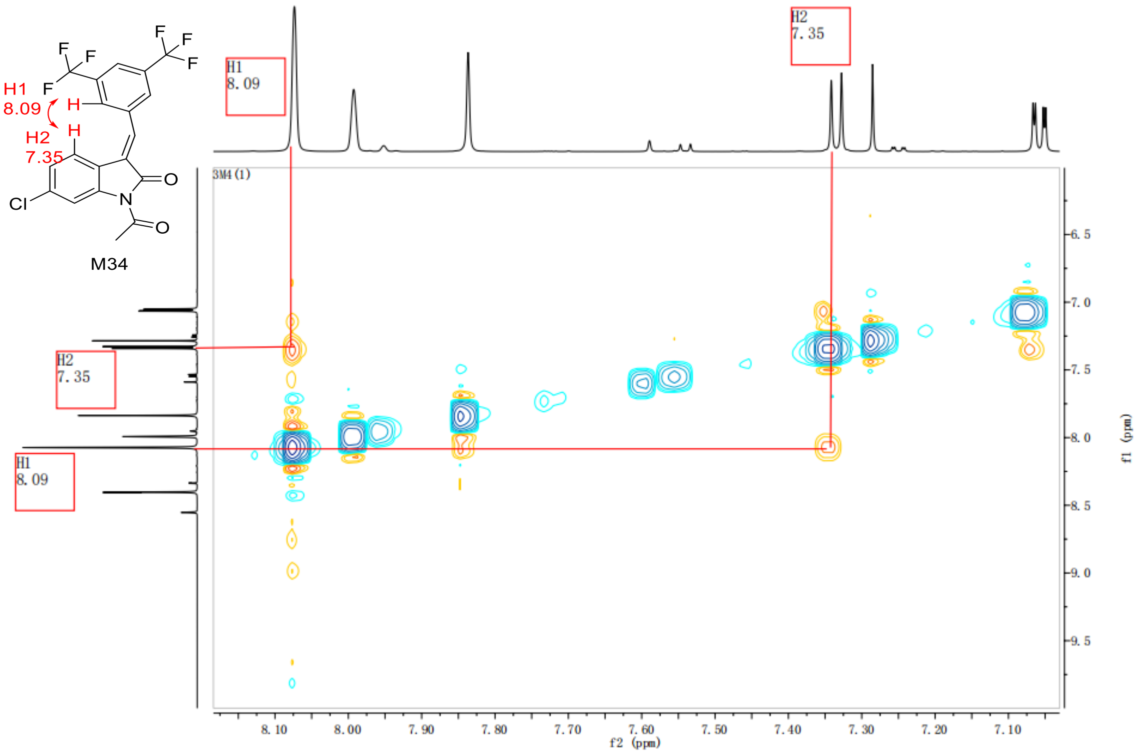

Figure S4-4. 2D-NOEY spectrum (600 Hz, $CDCl_3$) of compound **4d**

(*E*)1-acetyl-5-amino-3-(3-(trifluoromethyl)benzylidene)indolin-2-one (4e)

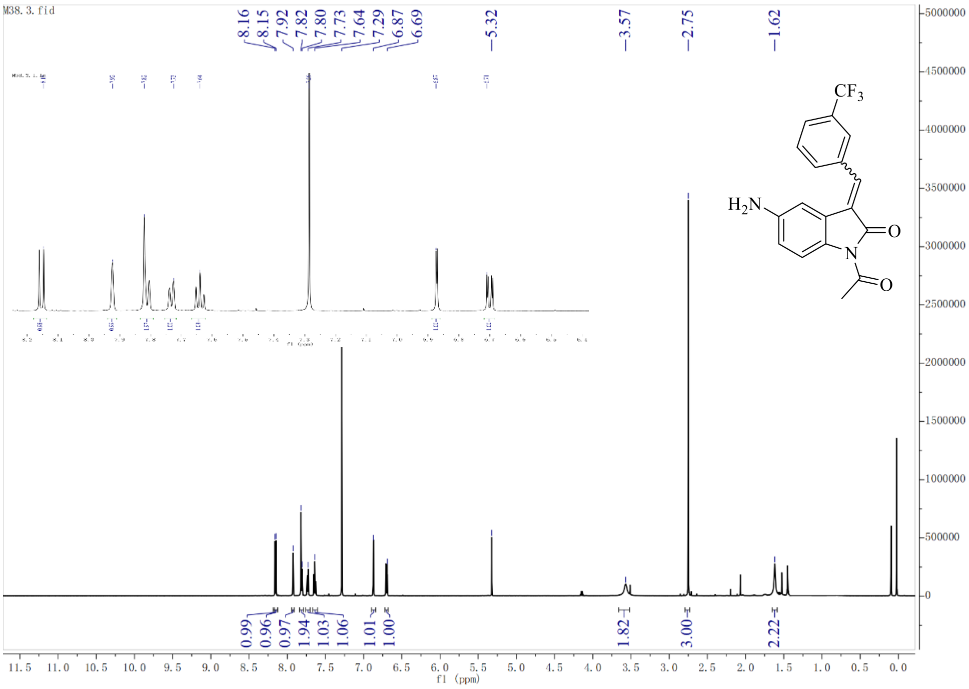

Figure S5-1. ^{1}H NMR spectrum (600 Hz, $CDCl_3$) of compound **4e**

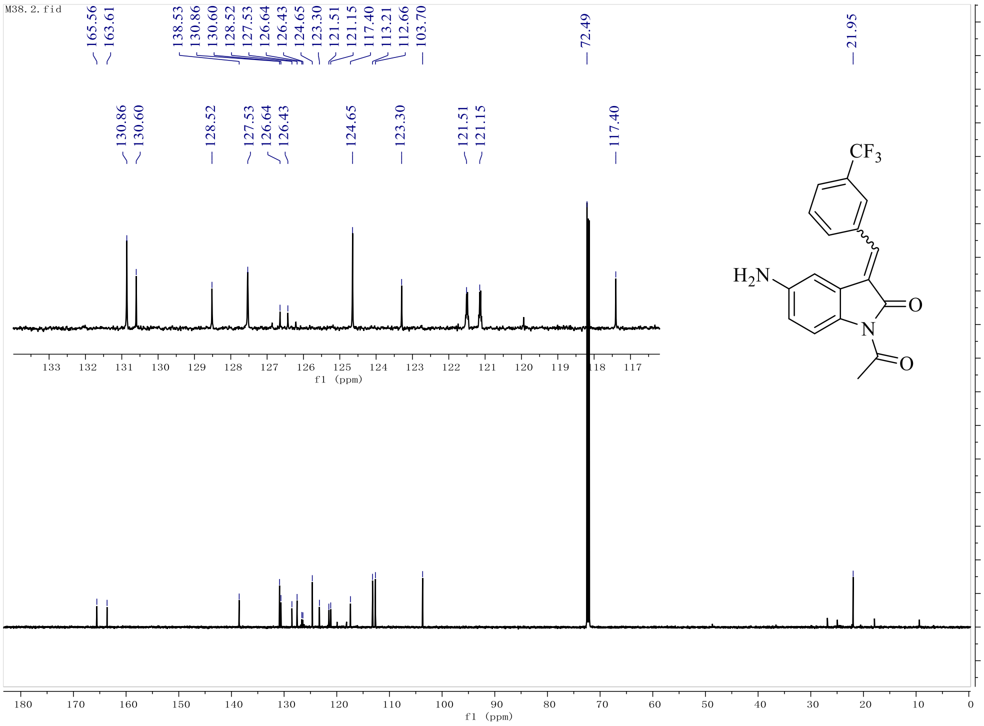

Figure S5-2. ^{1}H, ^{13}C NMR spectrum (150 Hz, DMSO) of compound **4e**

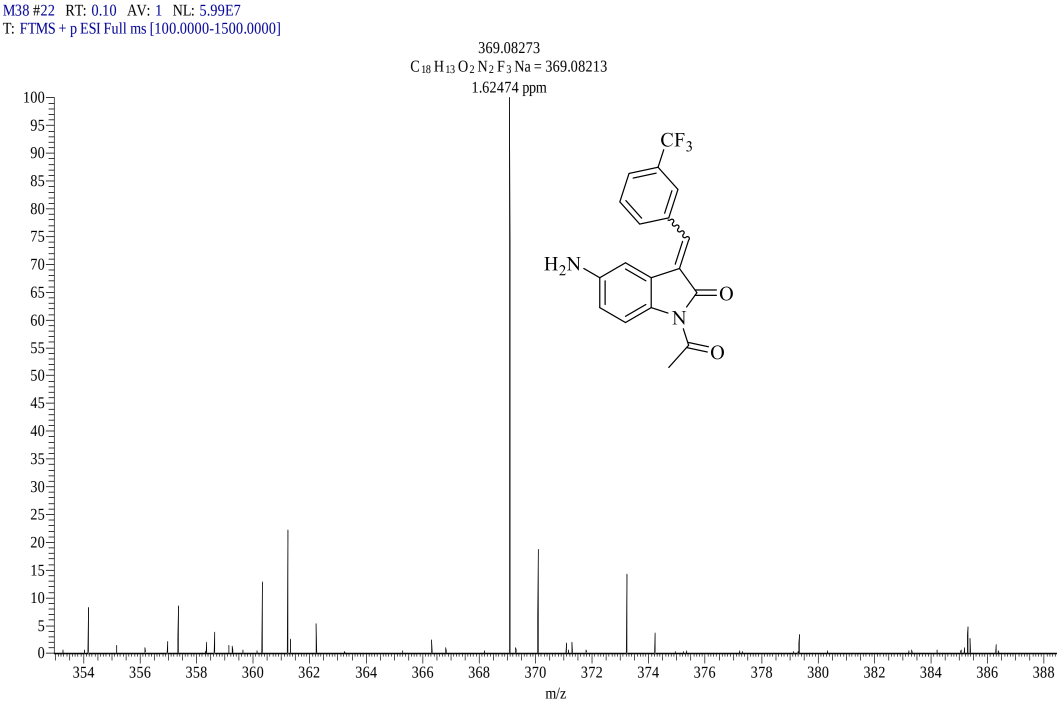

Figure S5-3. HRMS of compound **4e**

(Z)-1-acetyl-3-(3,5-bis(trifluoromethyl)benzylidene)-6-chloroindolin-2-one(4f)

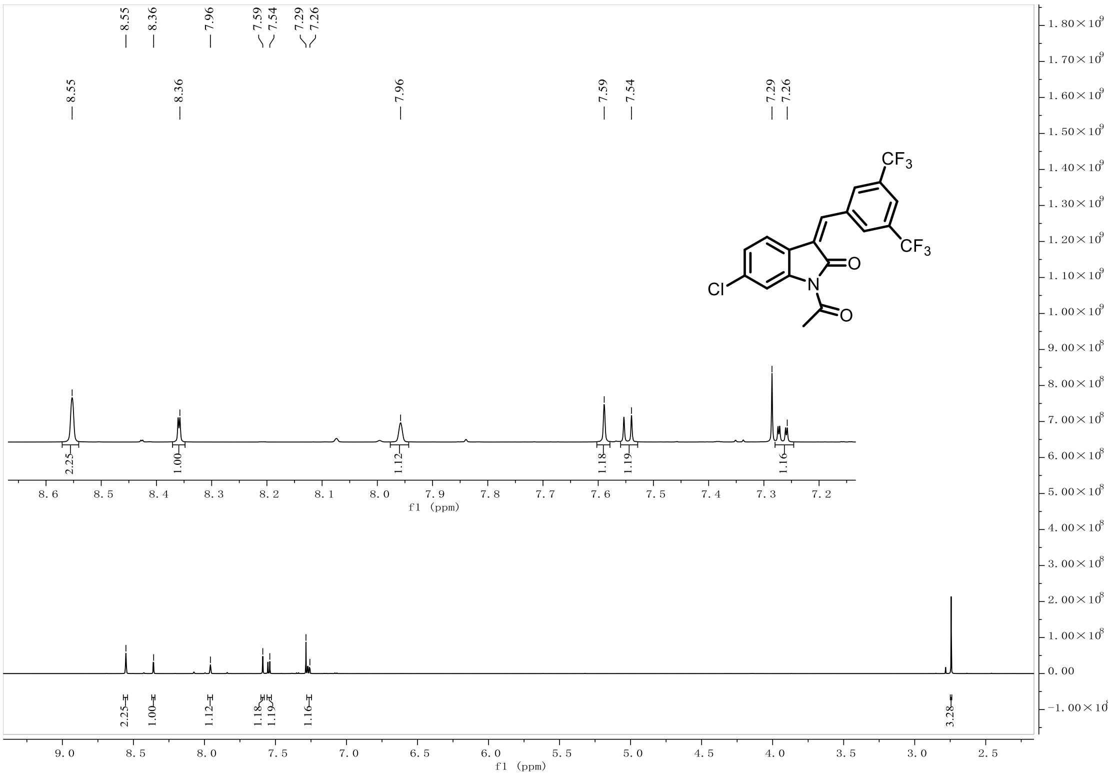

Figure S6-1. ^{1}H NMR spectrum (600 Hz, $CDCl_3$) of compound **4f**

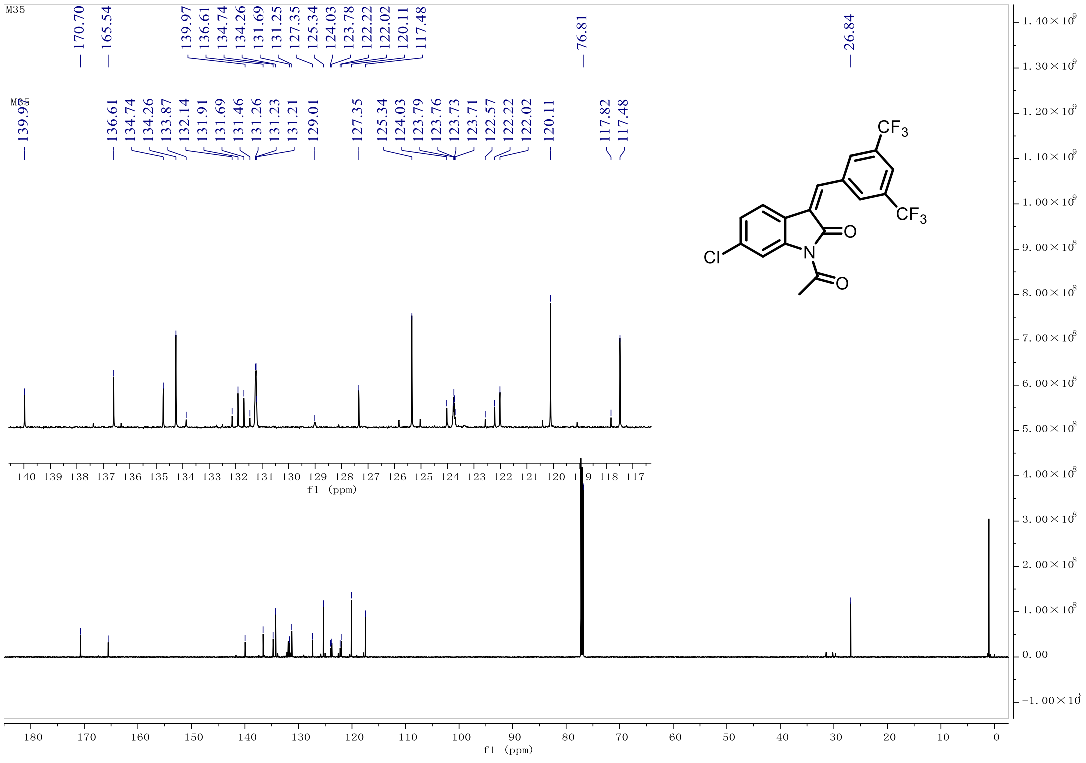

Figure S6-2. ^{1}H, ^{13}C NMR spectrum (600 Hz, 150 Hz, $CDCl_3$) of compound **4f**

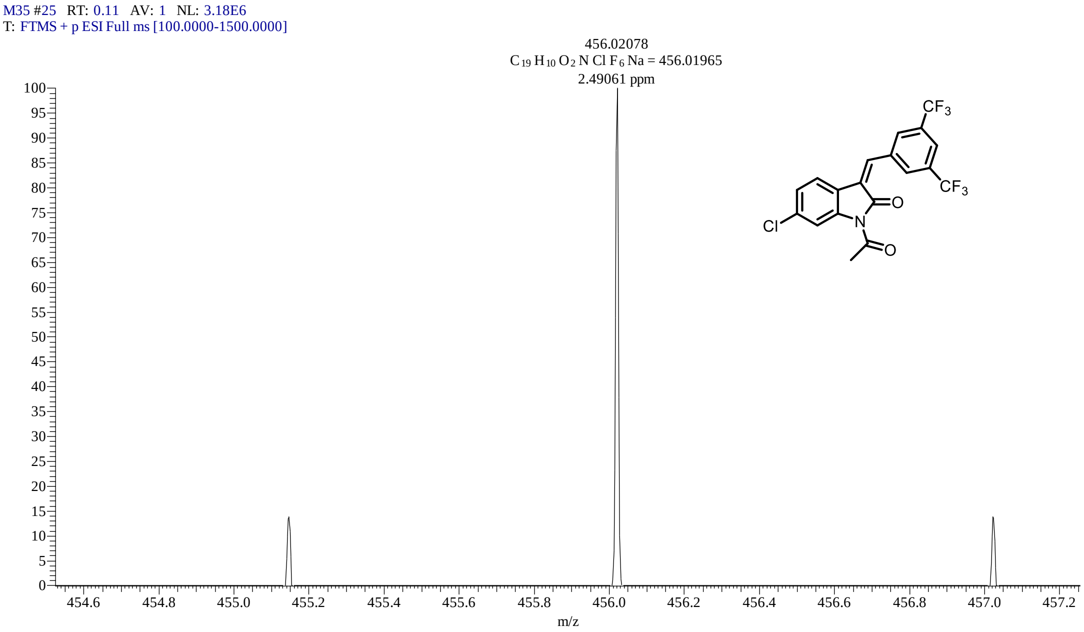

Figure S6-3. HRMS of compound **4f**

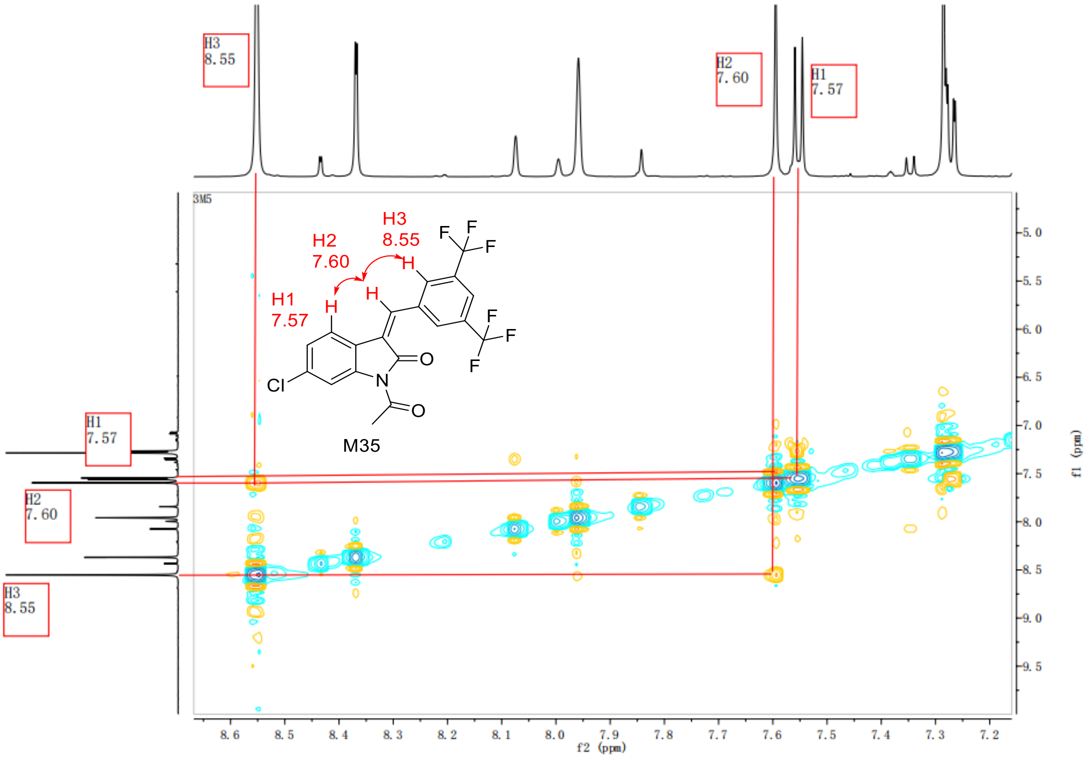

Figure S6-4. 2D-NOEY spectrum (600 Hz, $CDCl_3$) of compound **4f**

1-Acetyl-3-benzylideneindolin-2-one (4g)

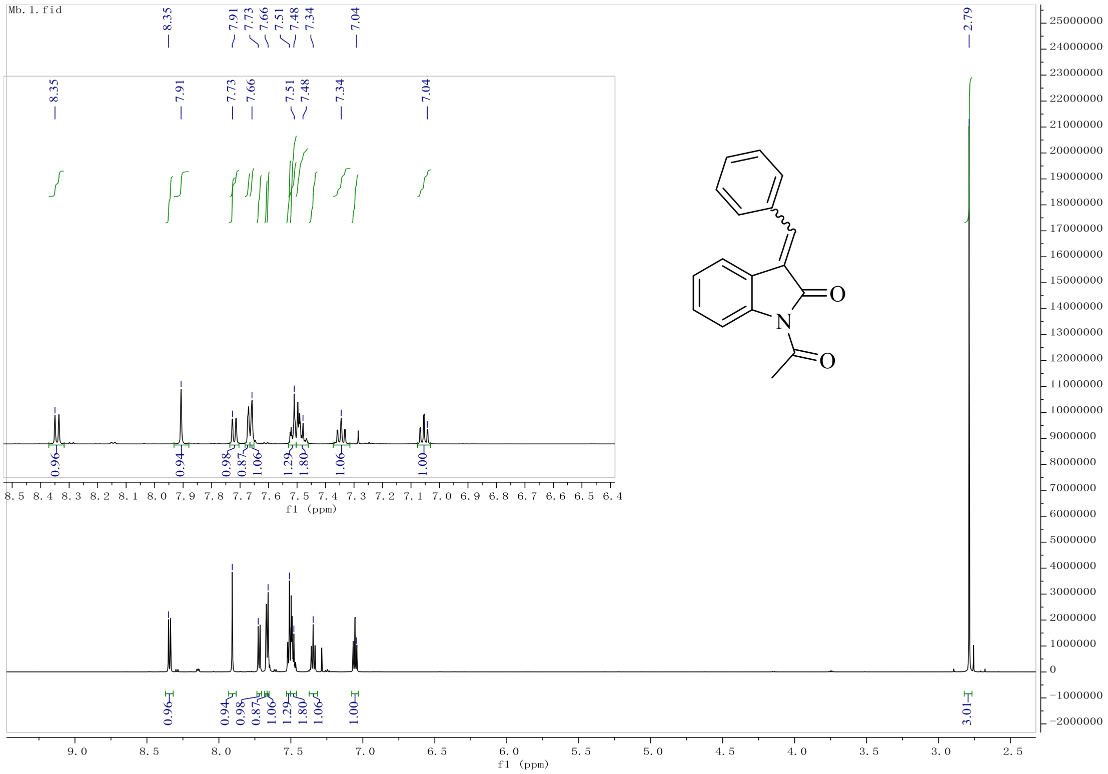

Figure S7-1. ^{1}H NMR spectrum (600 Hz, $CDCl_3$) of compound **4g**

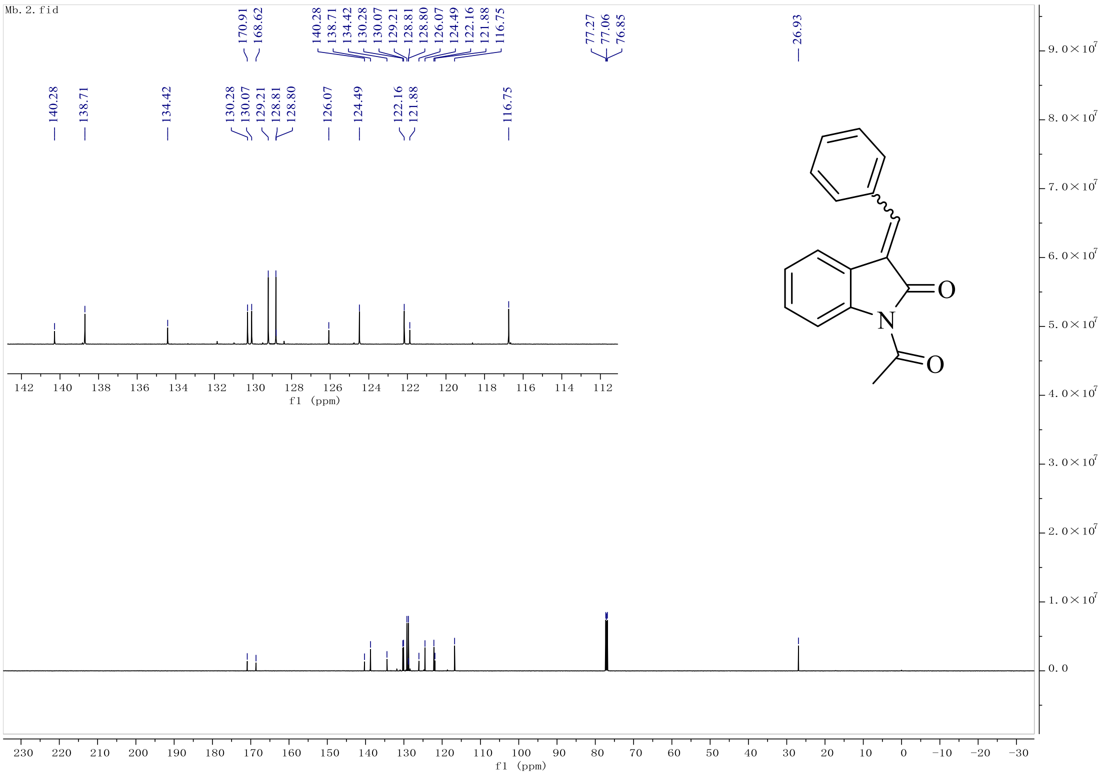

Figure S7-2. ^{13}C NMR spectrum (150 Hz, $CDCl_3$) of compound **4g**

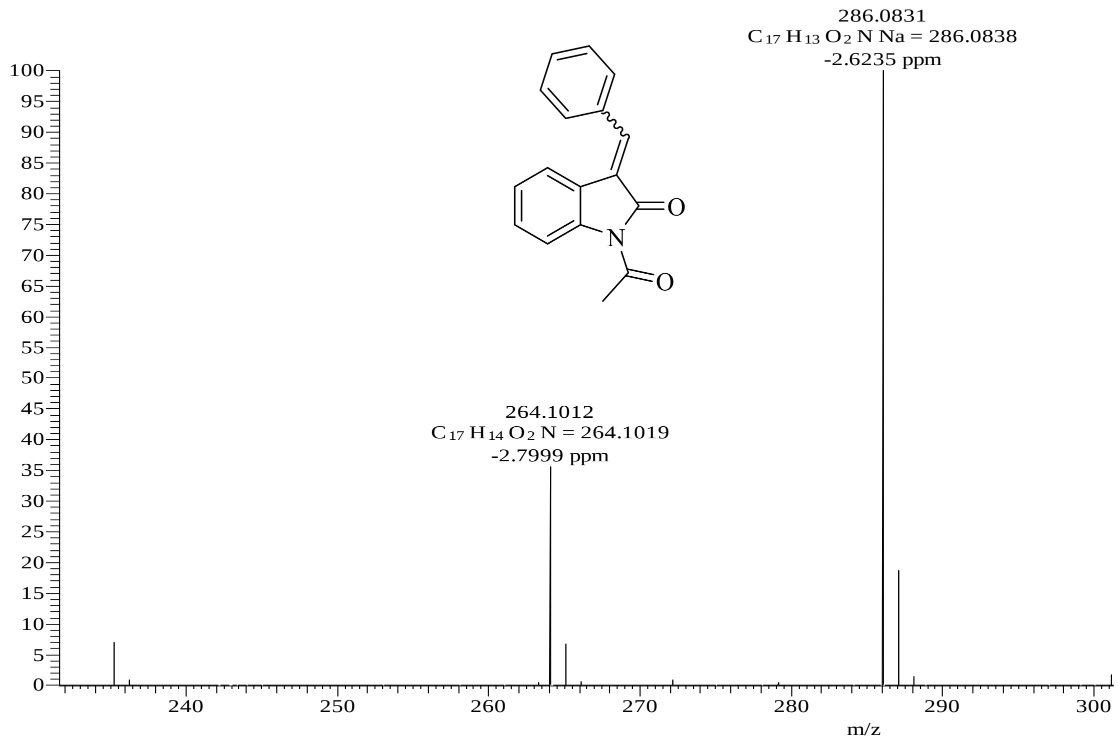

Figure S7-3. HRMS of compound **4g**

1-Acetyl-6-chloro-3-(3-(trifluoromethyl)benzylidene)indolin-2-one(4h)

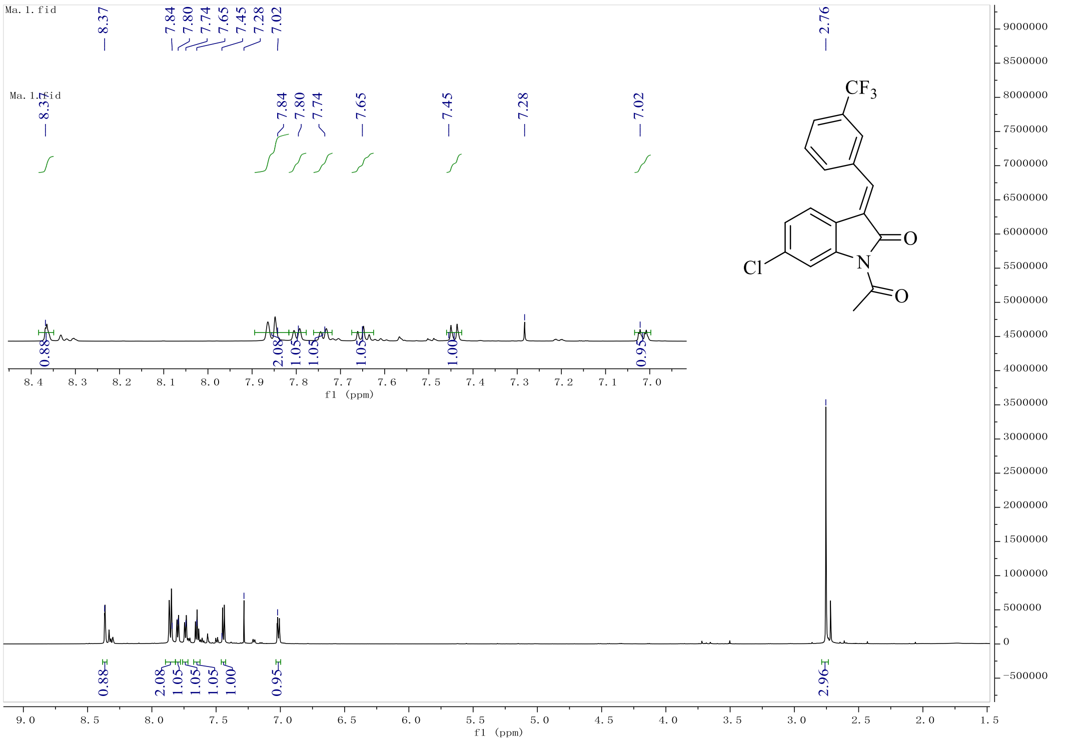

Figure S8-1. ^{1}H NMR spectrum (600 Hz, $CDCl_3$) of compound **4h**

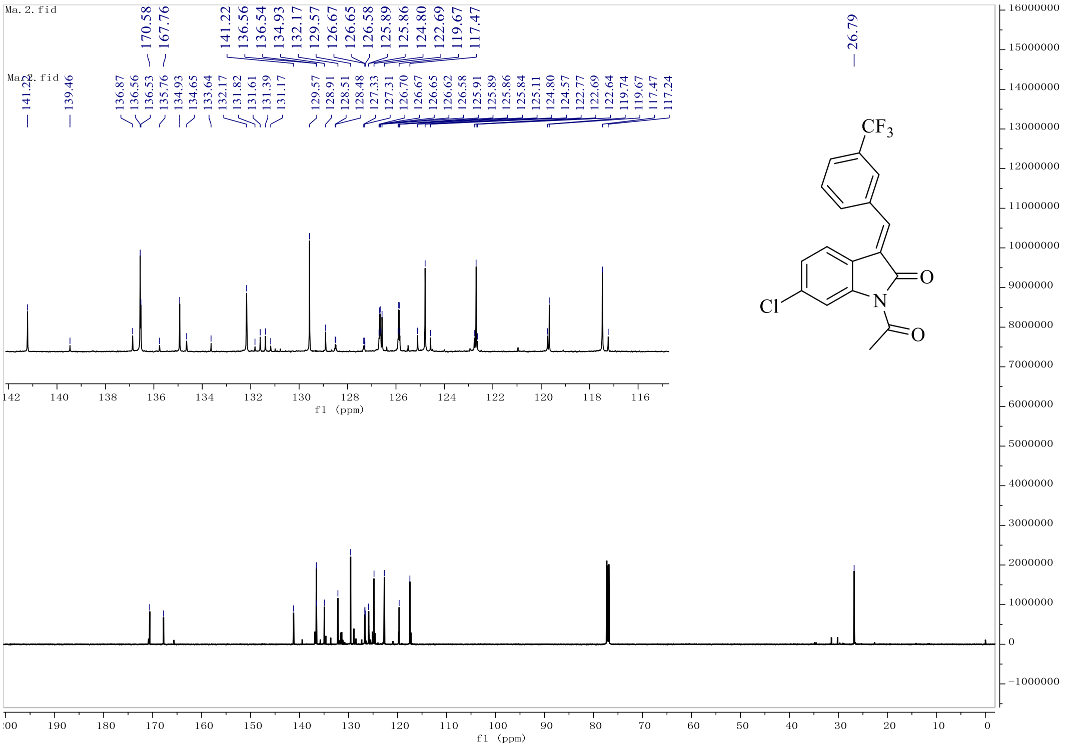

Figure S8-1. ^{13}C NMR spectrum (150 Hz, $CDCl_3$) of compound **4h**

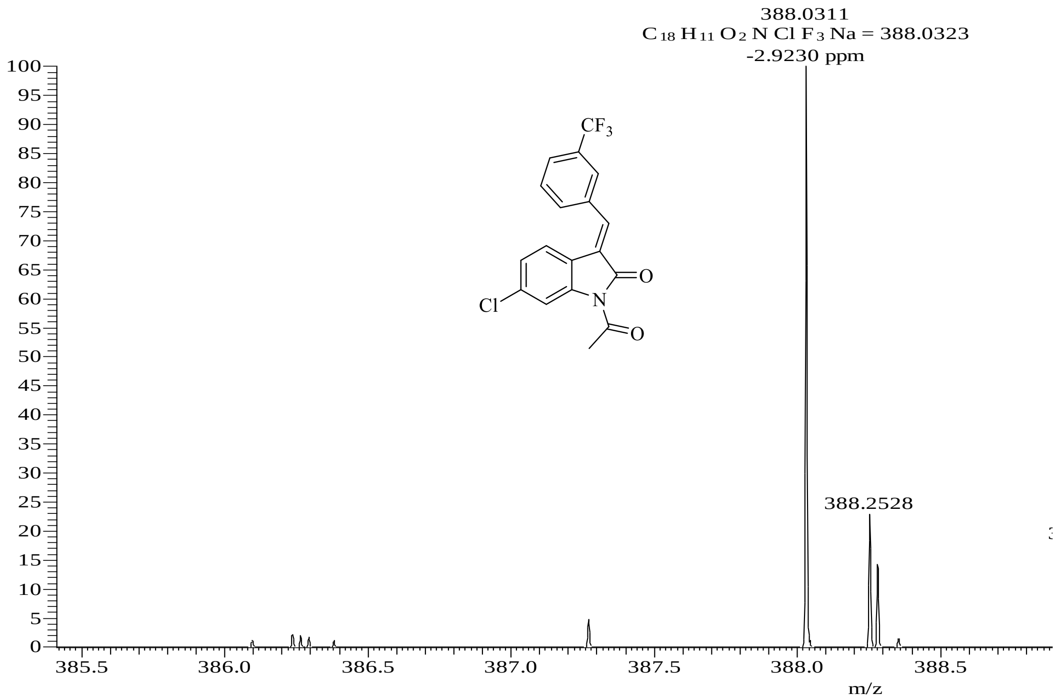

Figure S8-3. HRMS of compound **4h**

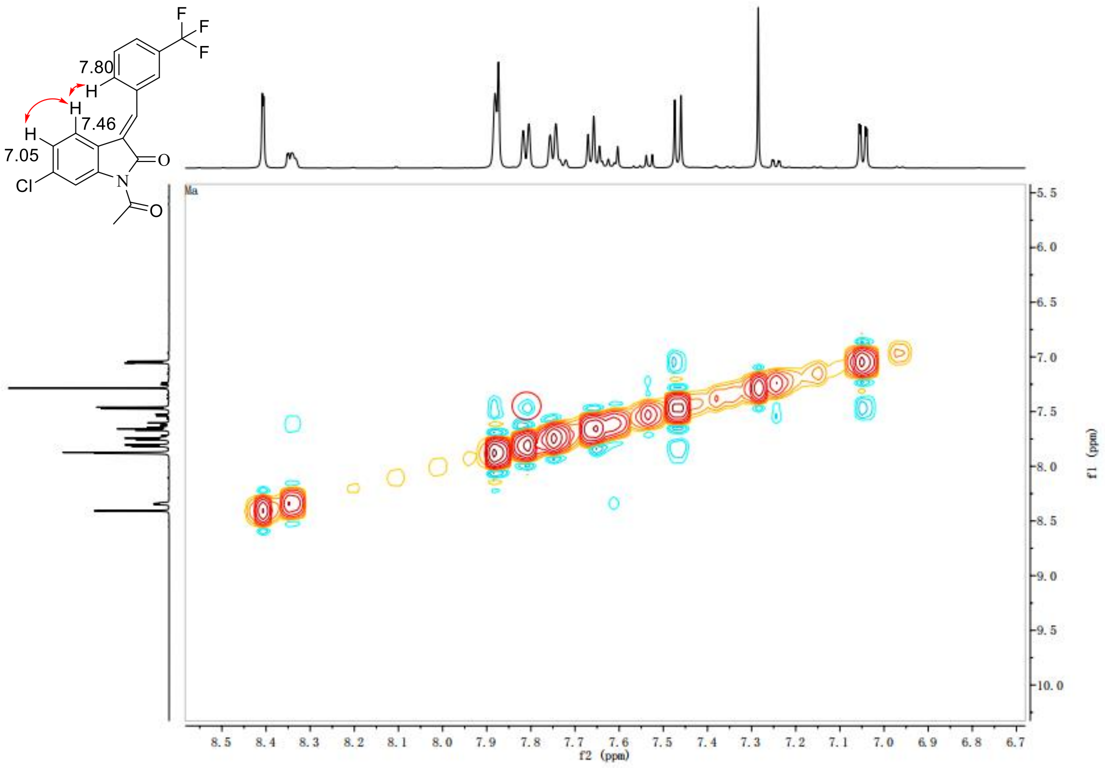

Figure S8-4. 2D-NOEY spectrum (600 Hz, $CDCl_3$) of compound **4h**

1-Acetyl-6-chloro-3-(4-(trifluoromethyl)benzylidene)indolin-2-one(4i)

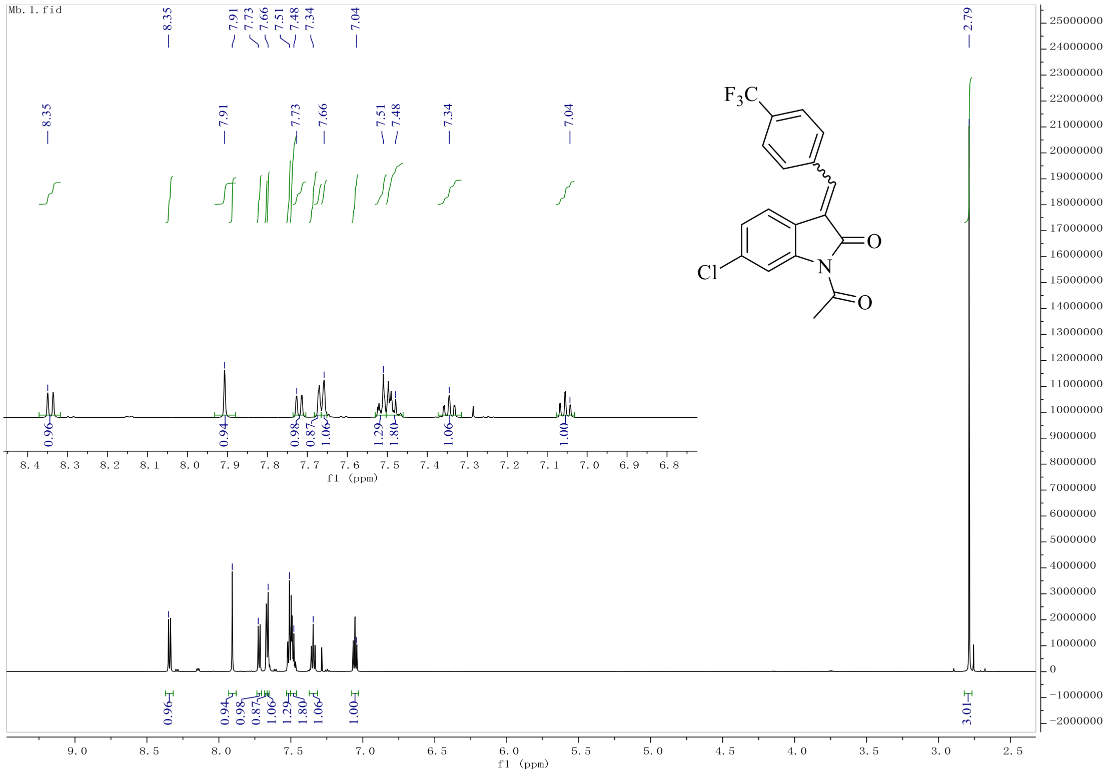

Figure S9-1. ^{1}H NMR spectrum (600 MHz, $CDCl_3$) of compound **4i**

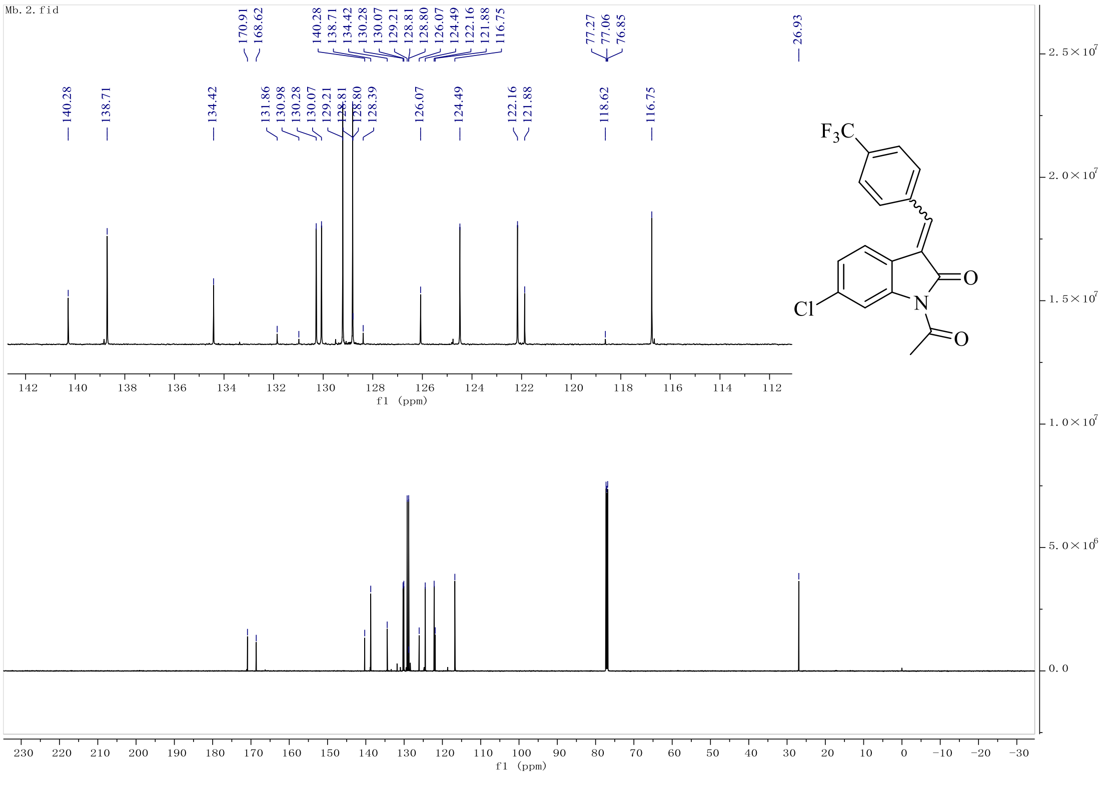

Figure S9-2. ^{13}C NMR spectrum (150 MHz, $CDCl_3$) of compound **4i**

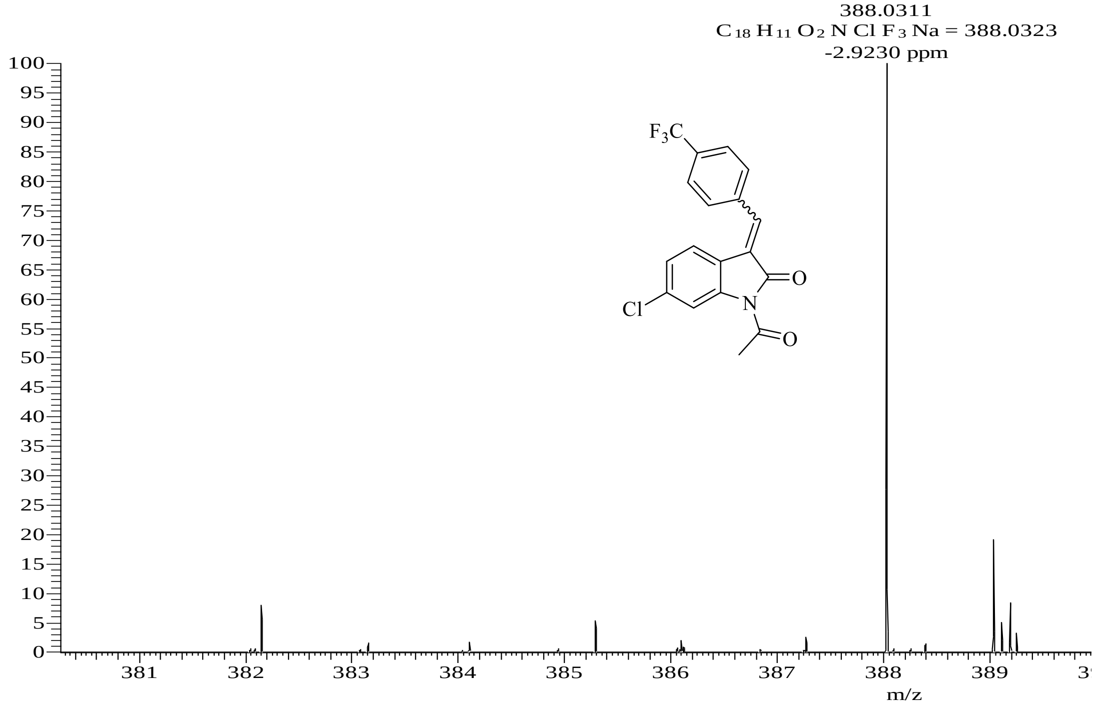

Figure S9-3. HRMS of compound **4i**

1-Acetyl-3-(3-(trifluoromethyl)benzylidene)indolin-2-one(4j)

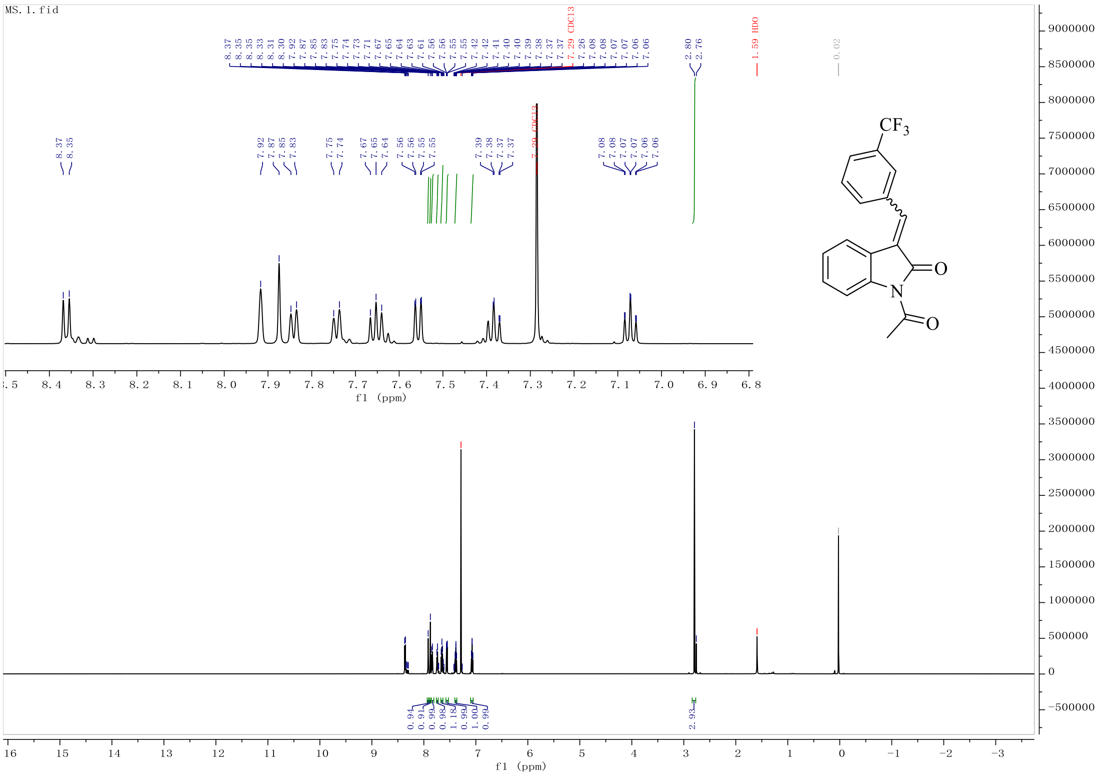

Figure S10-1. ^{1}H NMR spectrum (600 Hz, $CDCl_3$) of compound **4j**

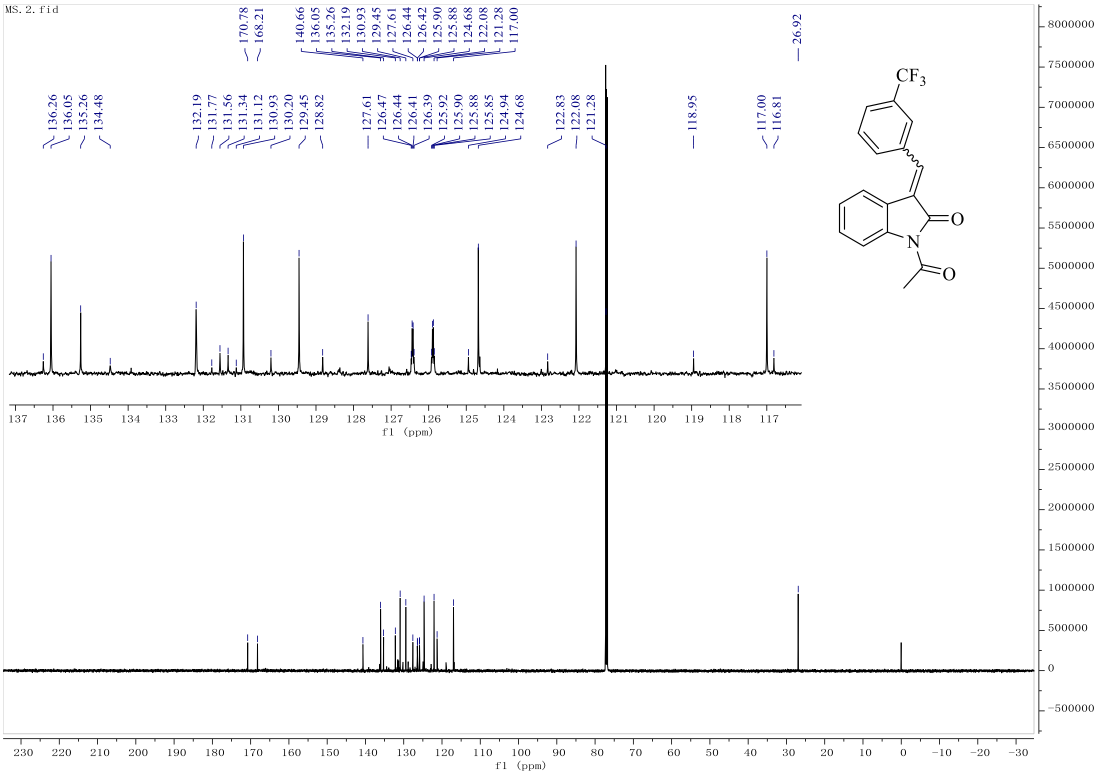

Figure S10-2. ^{13}C NMR spectrum (150 Hz, $CDCl_3$) of compound **4j**

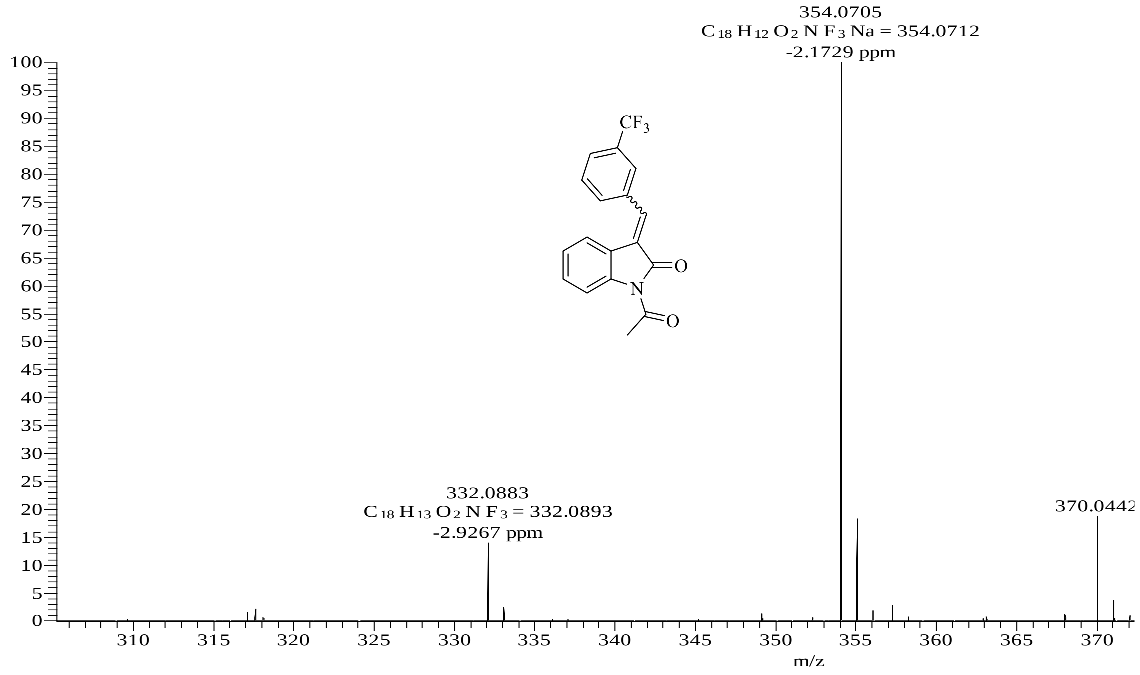

Figure S10-3. HRMS of compound **4j**

1-Acetyl-6-chloro-3-(2-(trifluoromethyl)benzylidene)indolin-2-one(4k)

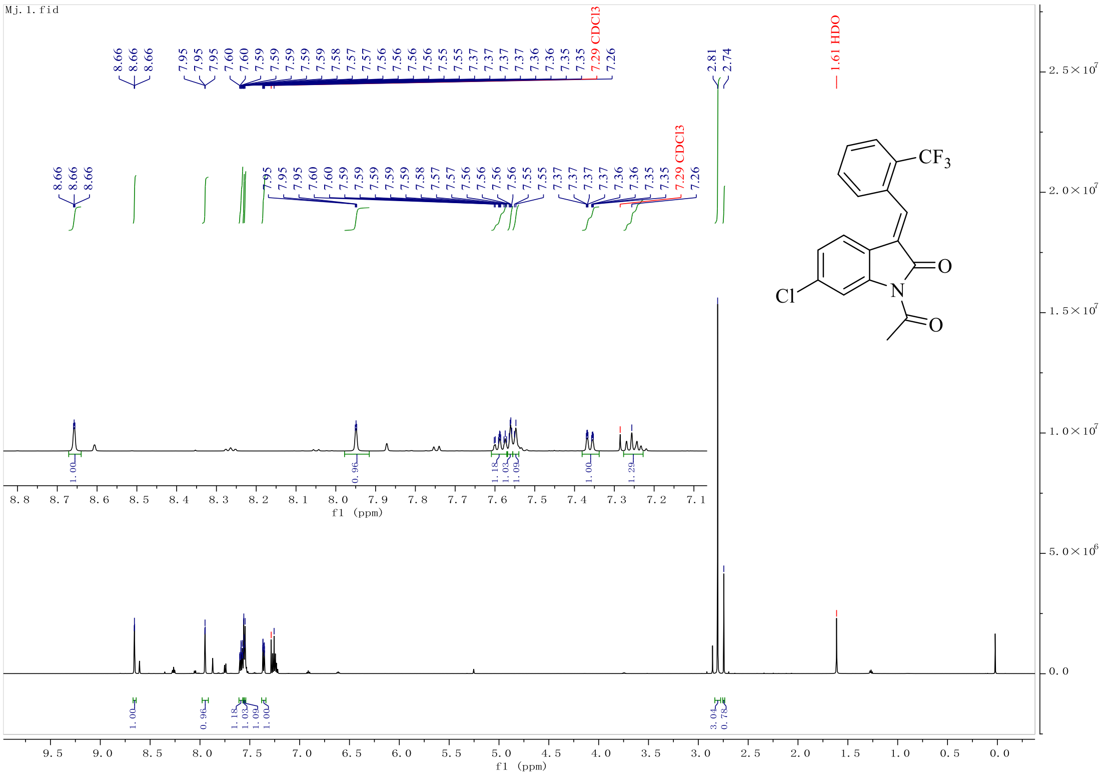

Figure S11-1. ^{1}H NMR spectrum (600 Hz, $CDCl_3$) of compound **4k**

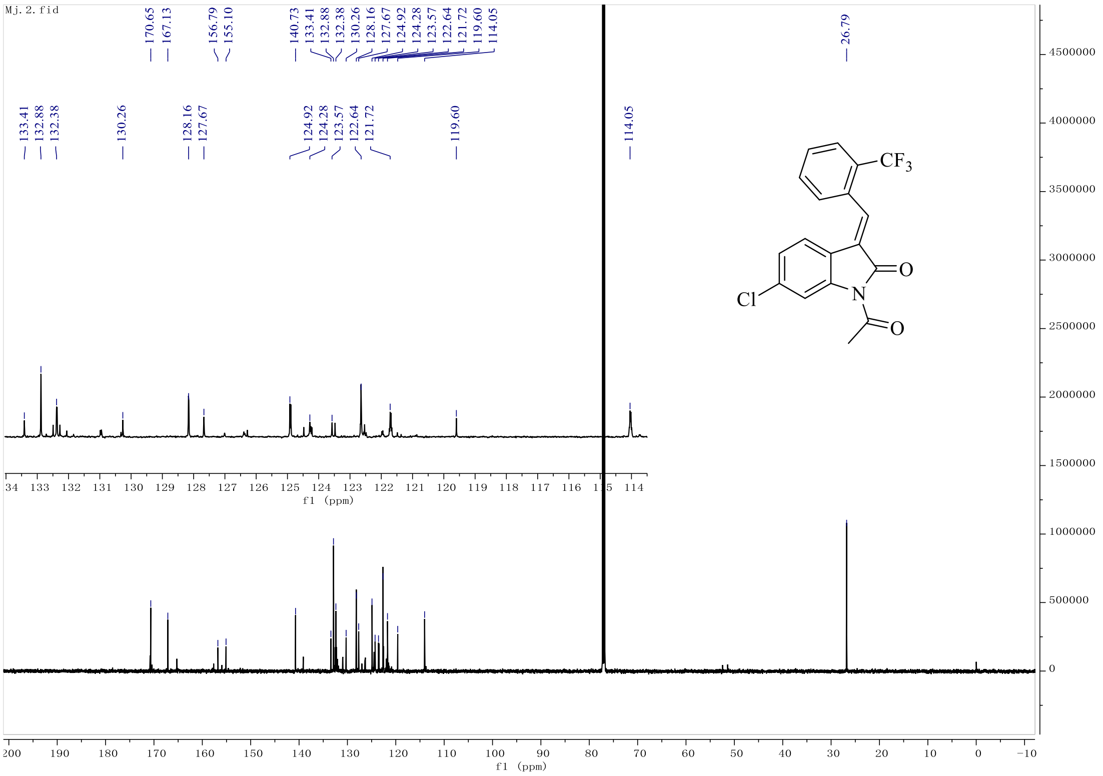

Figure S11-2. ^{13}C NMR spectrum (150 Hz, $CDCl_3$) of compound **4k**

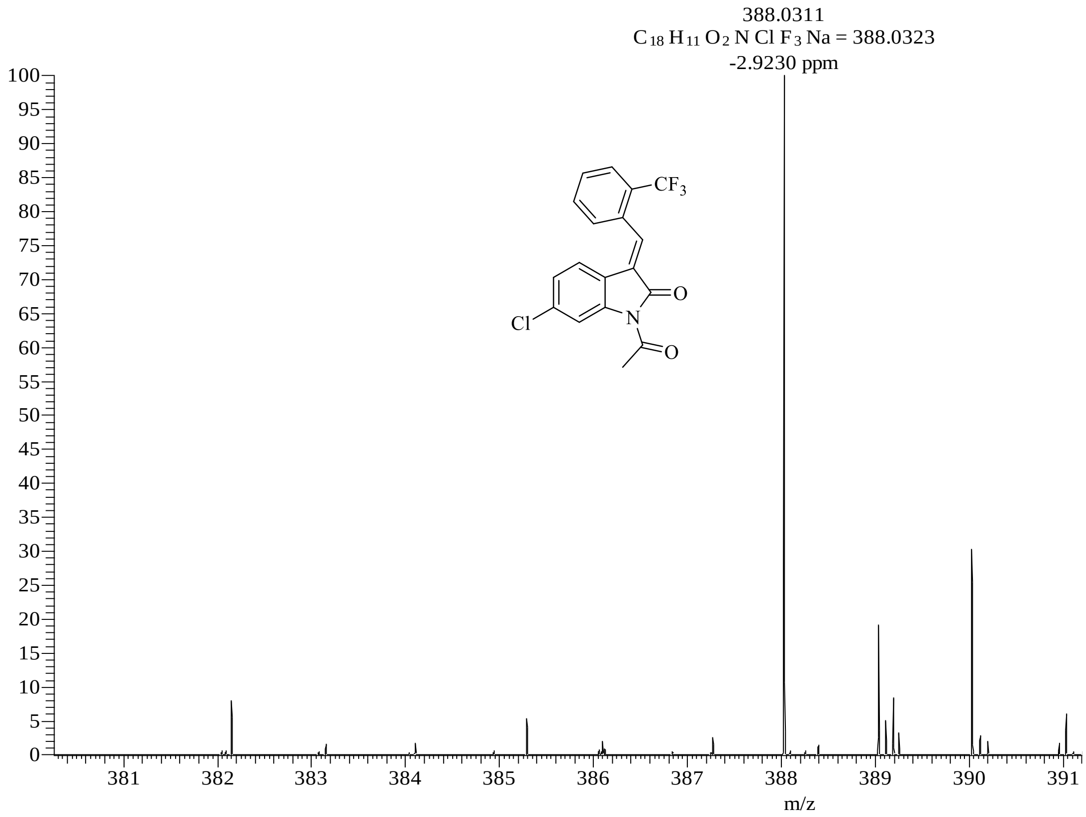

Figure S11-3. HRMS of compound **4k**

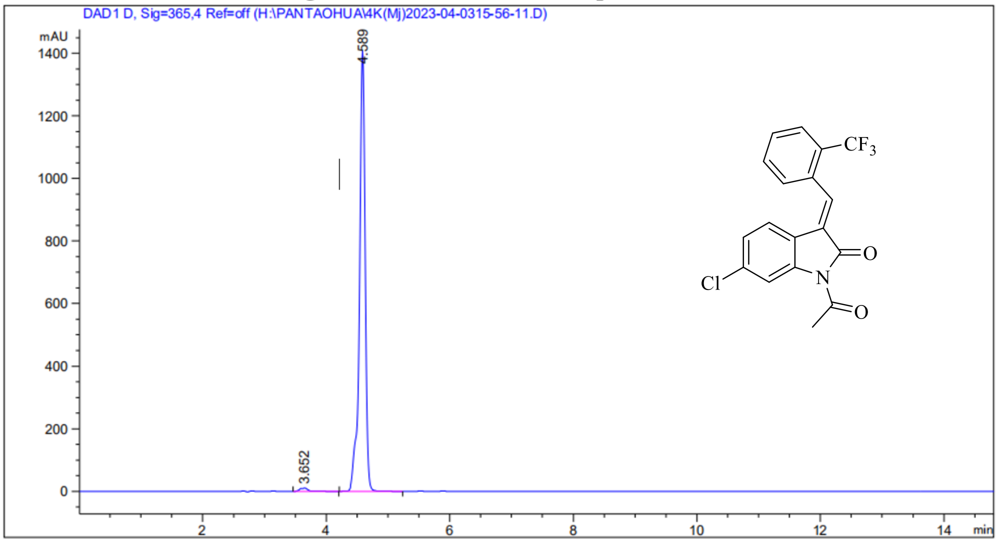

Peak #	RetTime [min]	Type	Width [min]	Area [mAU*s]	Height [mAU]	Area %
1	3.652	BV R	0.1241	92.01453	9.92733	1.0192
2	4.589	BB	0.0961	8936.00000	1406.58325	98.9808
Totals :				9028.01453	1416.51058	

Figure S11-4. HPLC of compound **4k**

1-Acetyl-6-chloro-3-(4-chloro-3-(trifluoromethyl)benzylidene)indolin-2-one(4l)

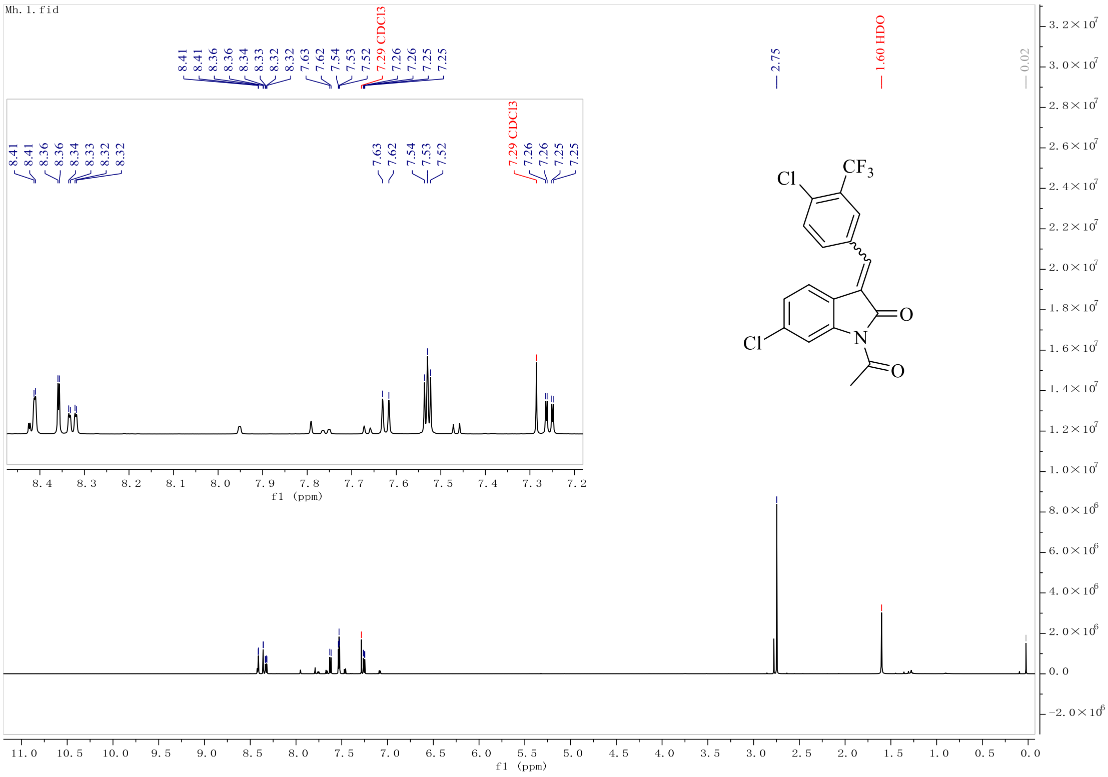

Figure S12-1. ^{1}H, NMR spectrum (600 Hz, $CDCl_3$) of compound **4l**

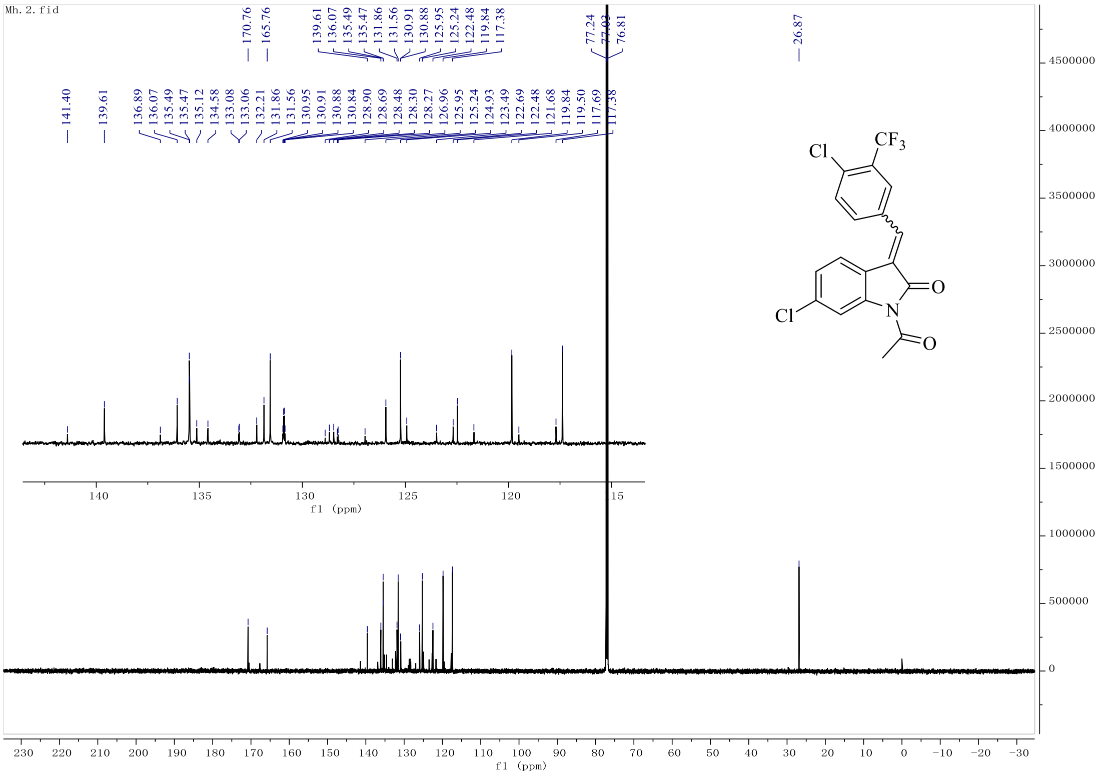

Figure S12-2. ^{13}C NMR spectrum (150 Hz, $CDCl_3$) of compound **4l**

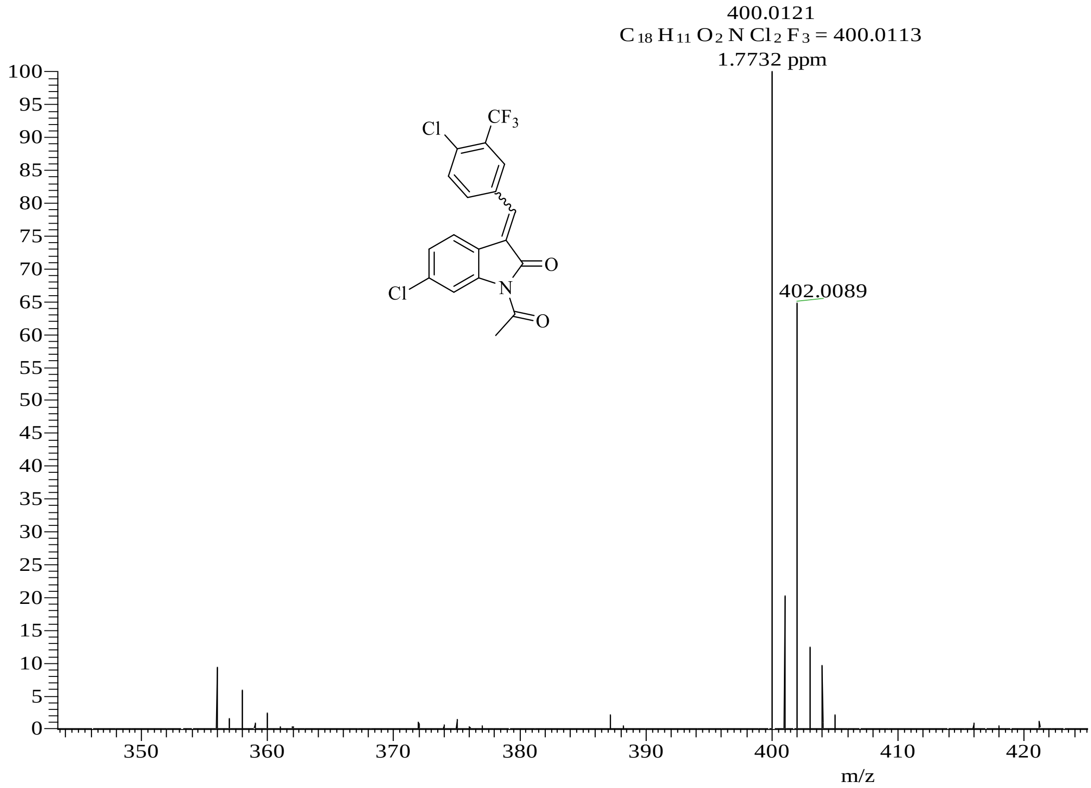

Figure S12-3. HRMS of compound **4l**

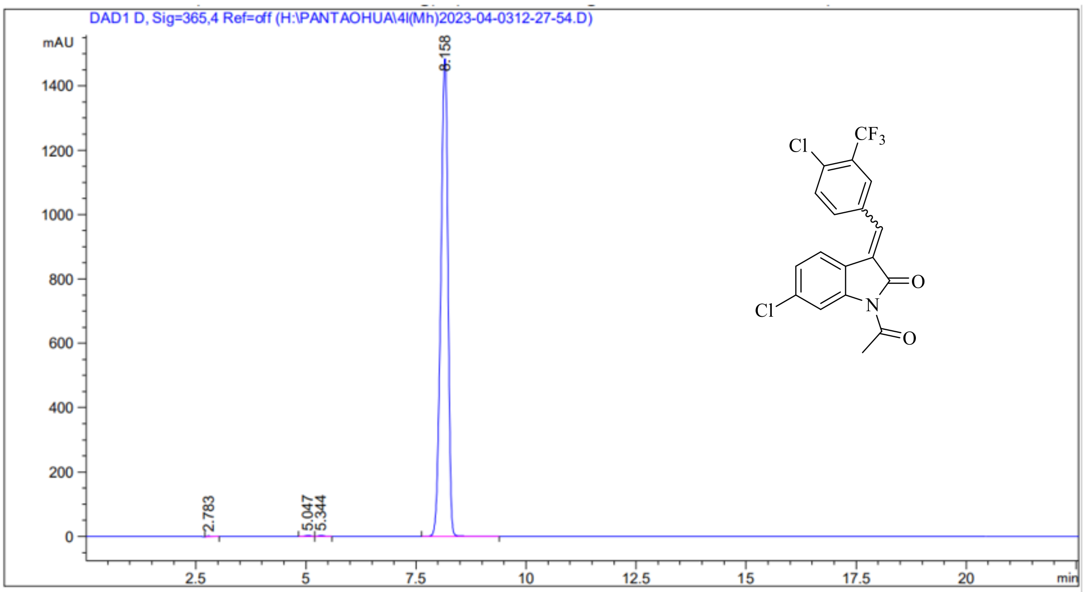

Peak #	RetTime [min]	Type	Width [min]	Area [mAU*s]	Height [mAU]	Area %
1	2.783	BB	0.1095	12.15048	1.65704	0.0717
2	5.047	BV	0.0993	21.11228	3.27005	0.1246
3	5.344	VB	0.1065	20.82145	3.01587	0.1229
4	8.158	BB	0.1807	1.68834e4	1484.52014	99.6807

Figure S12-4. HPLC of compound **4l**

1-Acetyl-6-chloro-3-(4-chloro-3-fluorobenzylidene)indolin-2-one(4m)

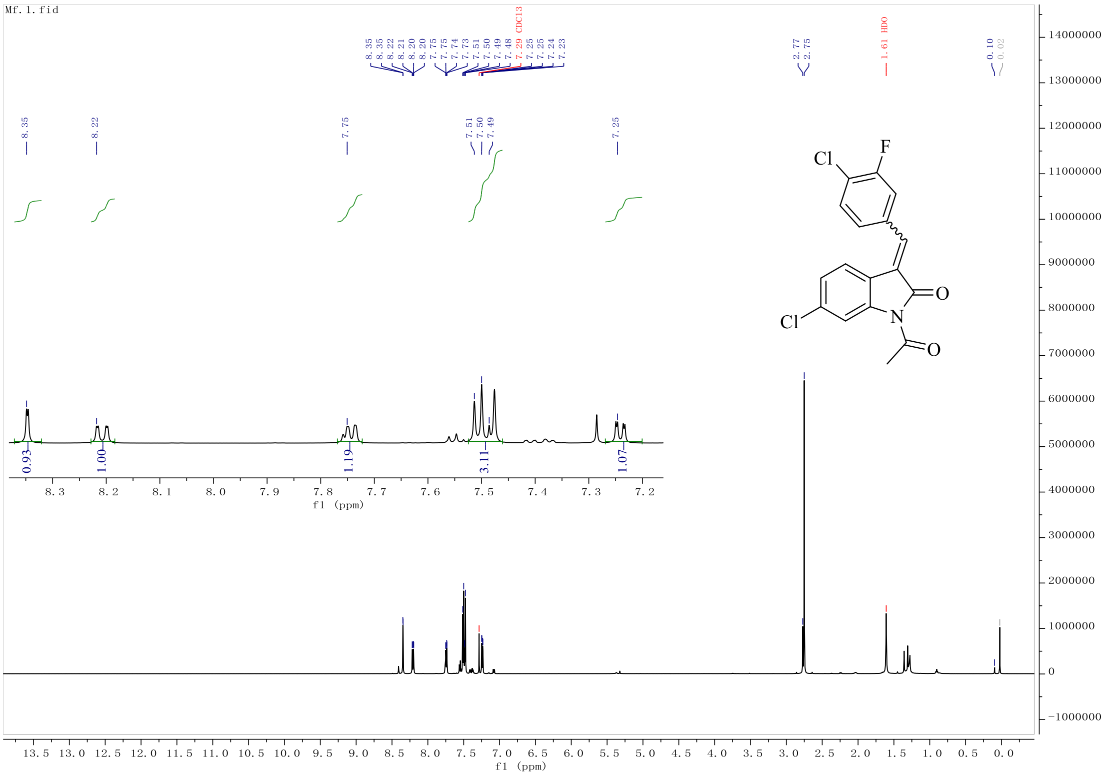

Figure S13-1. ^{1}H NMR spectrum (600 MHz $CDCl_3$) of compound **4m**

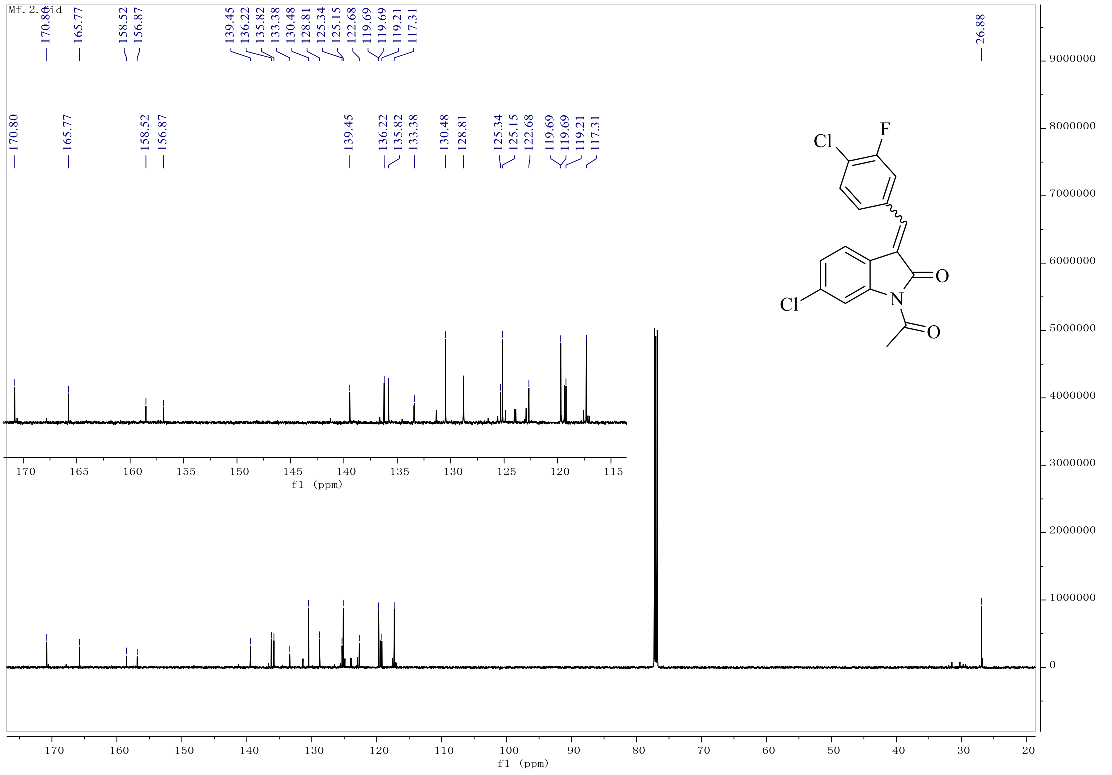

Figure S13-2. ^{13}C NMR spectrum (150 MHz $CDCl_3$) of compound **4m**

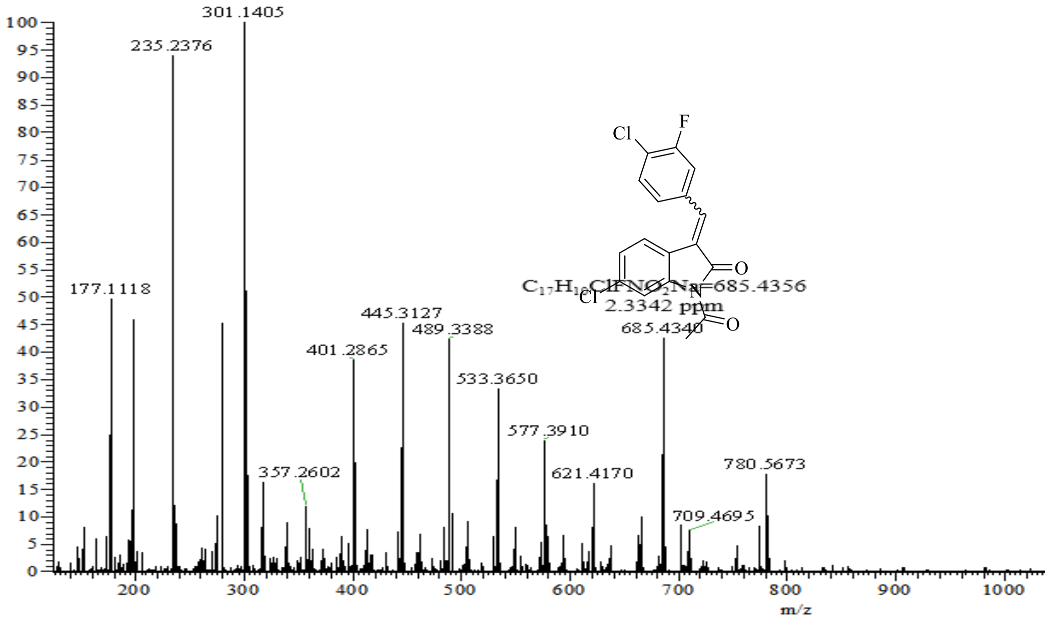

Figure S13-3. HRMS of compound **4m**

1-Acetyl-6-chloro-3-(2, 3-difluorobenzylidene)indolin-2-one(4n)

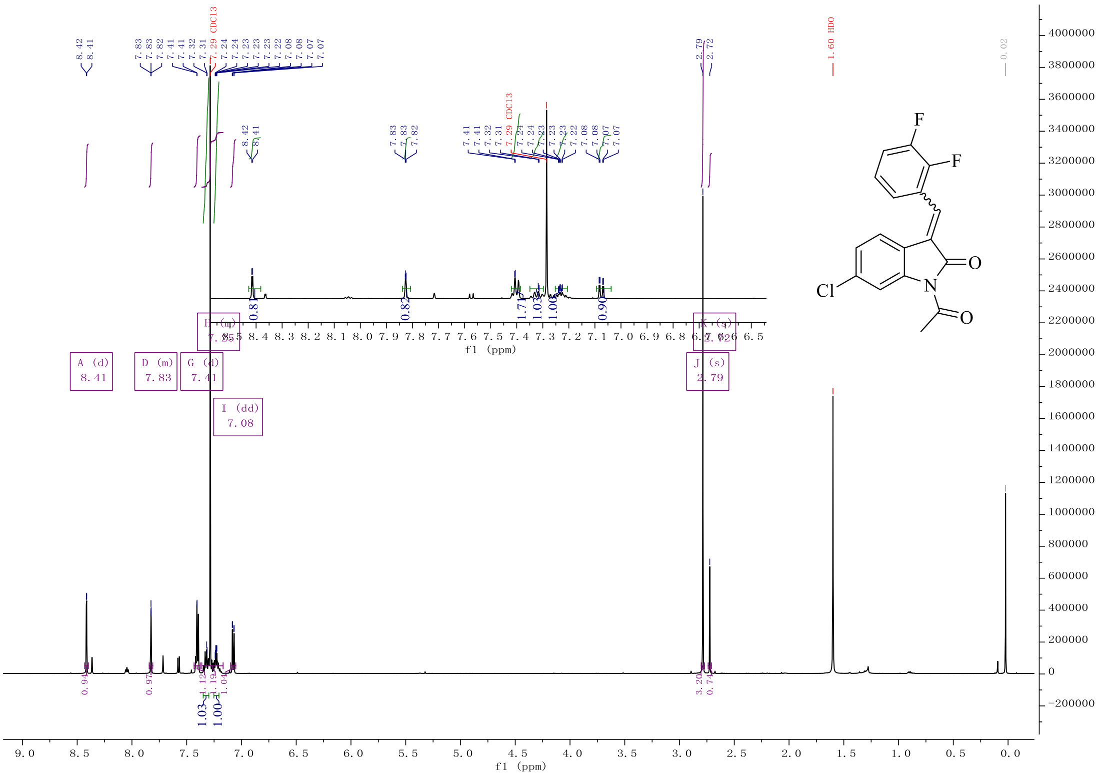

Figure S14-1. ^{1}H NMR spectrum (600 MHz, $CDCl_3$) of compound **4n**

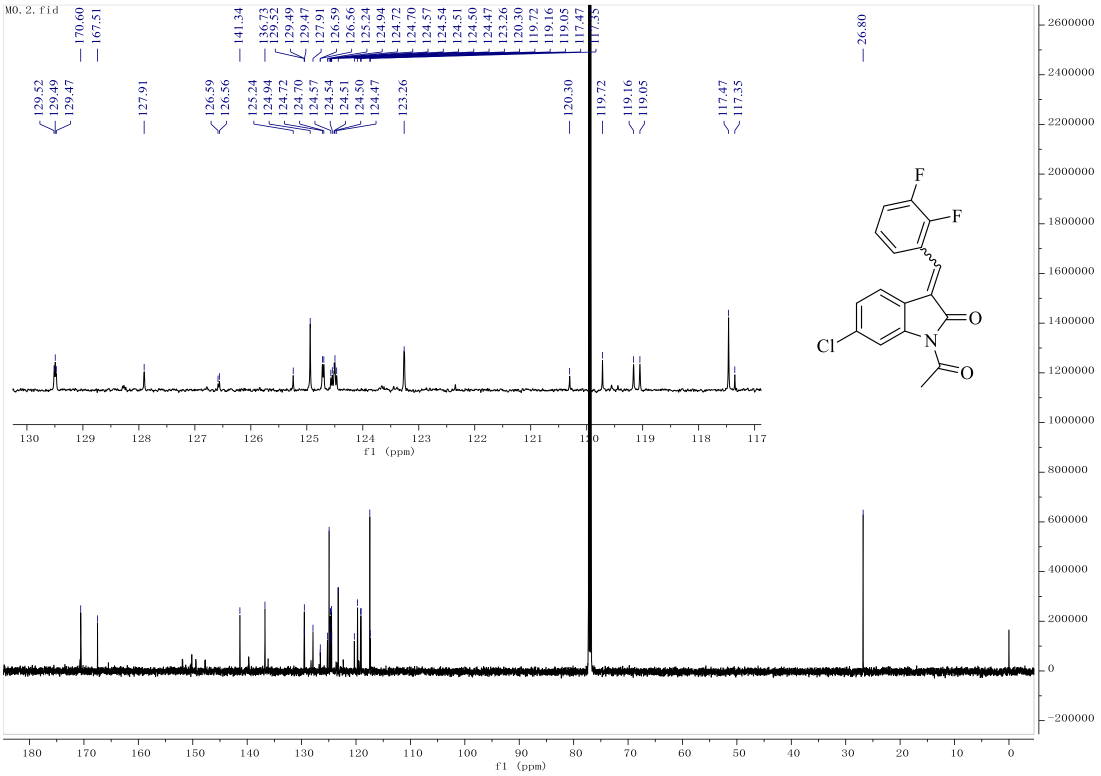

Figure S14-2. ^{13}C NMR spectrum (150 MHz, $CDCl_3$) of compound **4n**

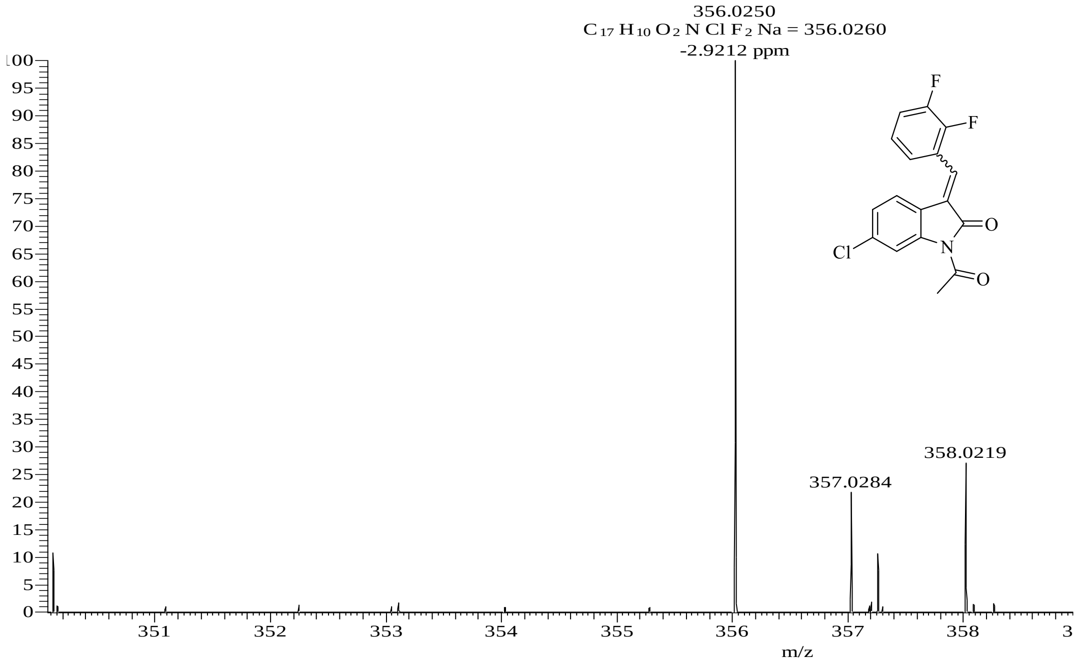

Figure S14-3. HRMS of compound **4n**

1-Acetyl-5-chloro-3-(3-chloro-2-fluorobenzylidene)indolin-2-one(4o)

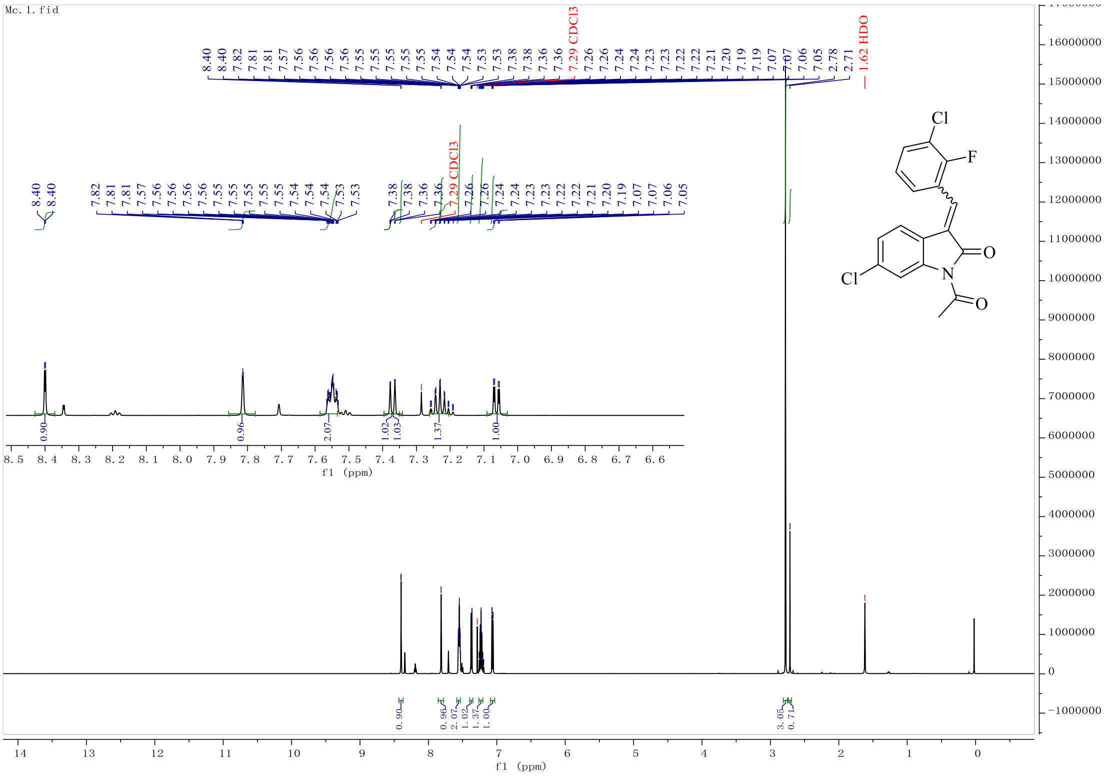

Figure S15-1. ^{1}H NMR spectrum (600 MHz, $CDCl_3$) of compound **4o**

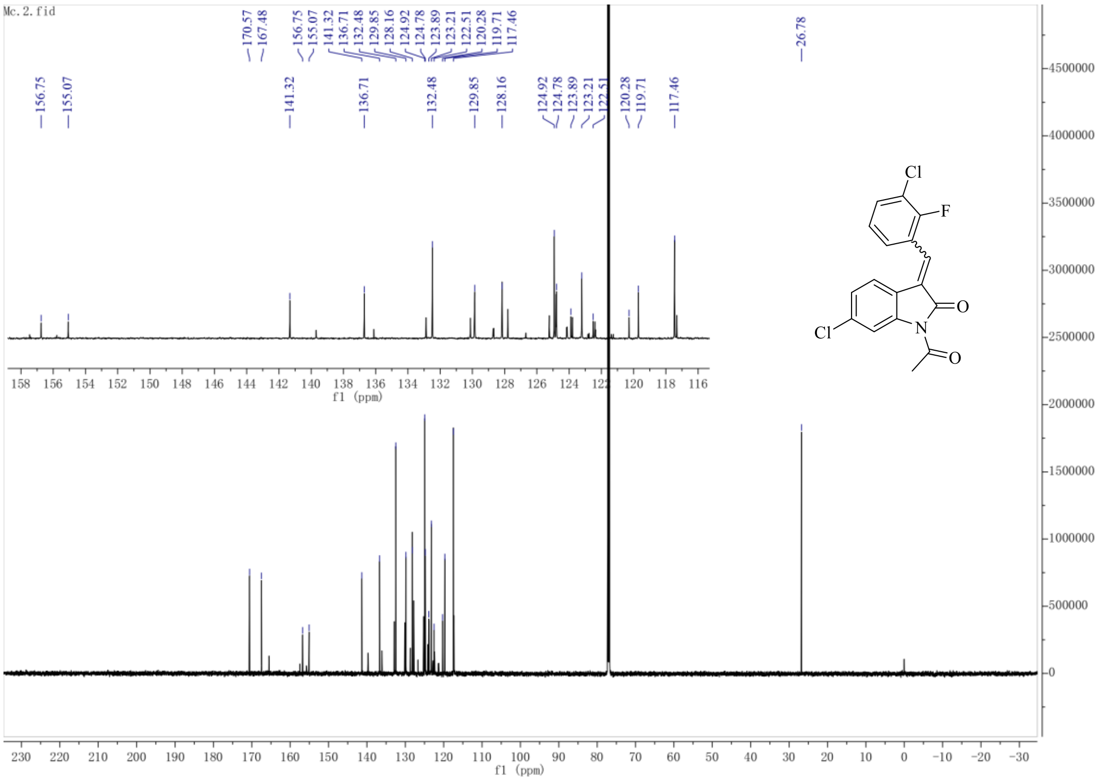

Figure S15-2. ^{13}C NMR spectrum (150 MHz, $CDCl_3$) of compound **4o**

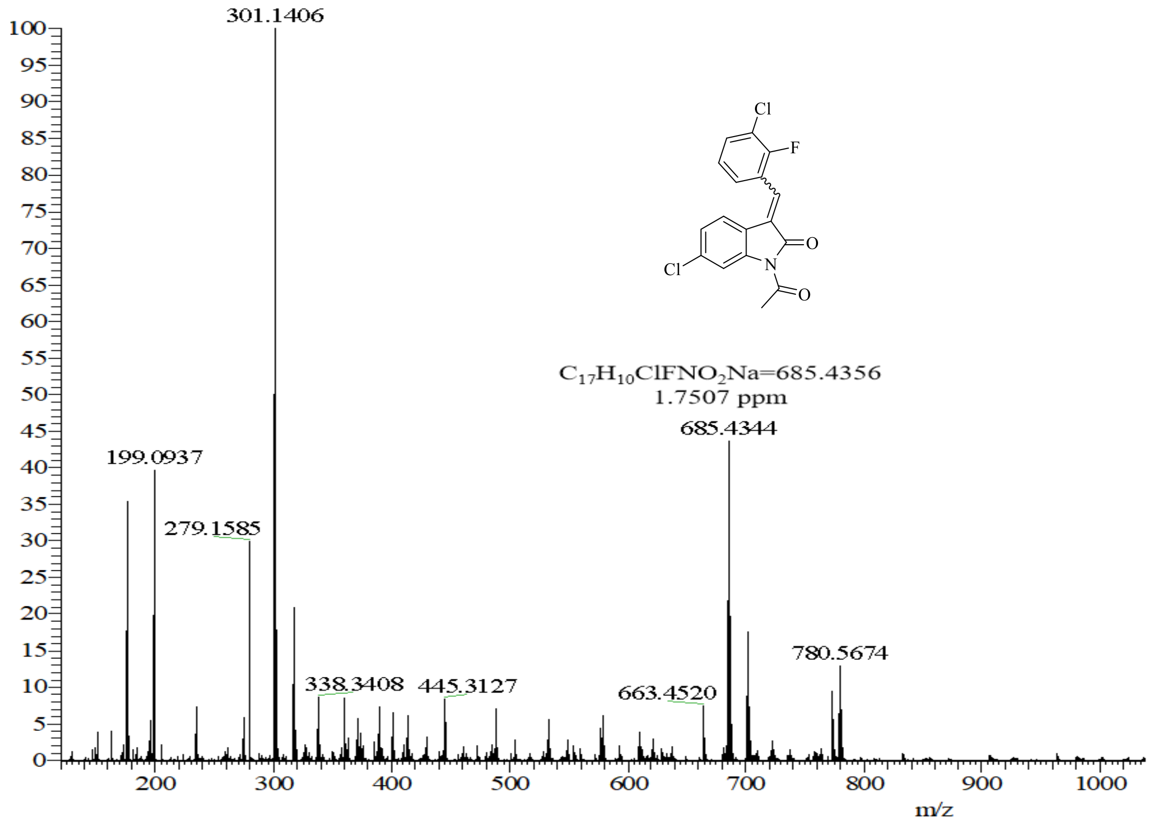

Figure S15-3. HRMS of compound **4o**

1-Acetyl-3-(3-chloro-2-fluorobenzylidene)-6-(trifluoromethyl)indolin-2-one (4p)

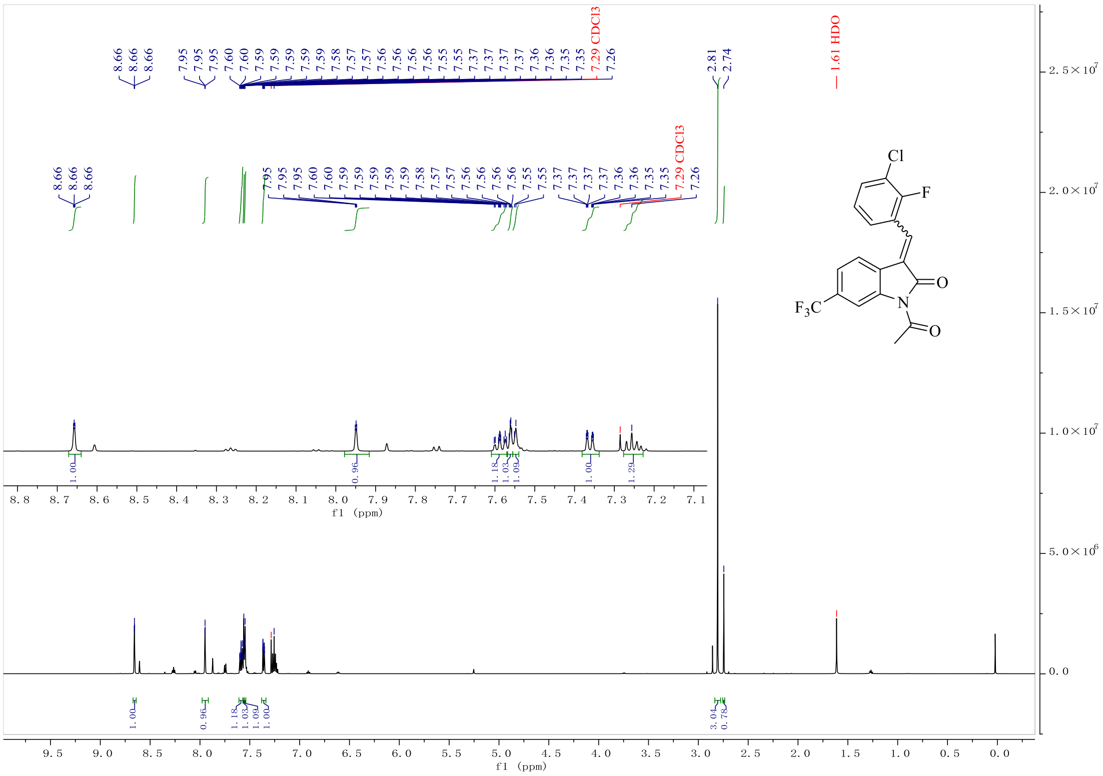

Figure S16-1. ^{1}H NMR spectrum (600 MHz, $CDCl_3$) of compound **4p**

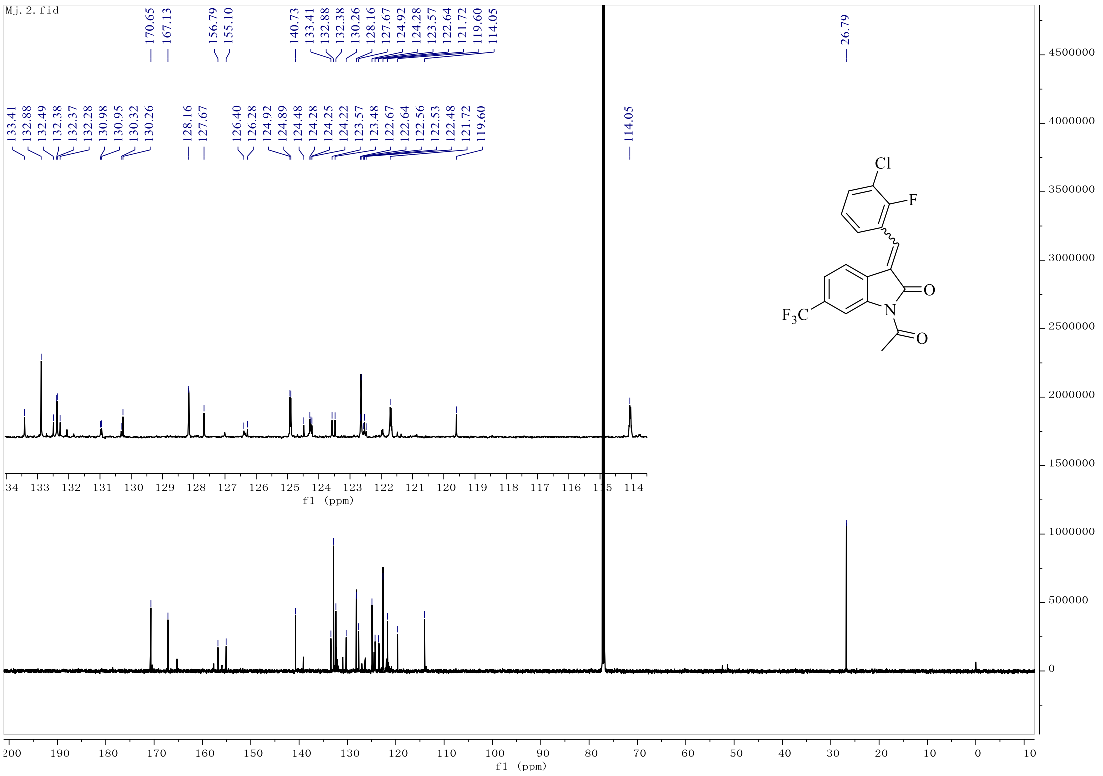

Figure S16-2. ^{13}C NMR spectrum (150 MHz, $CDCl_3$) of compound **4p**

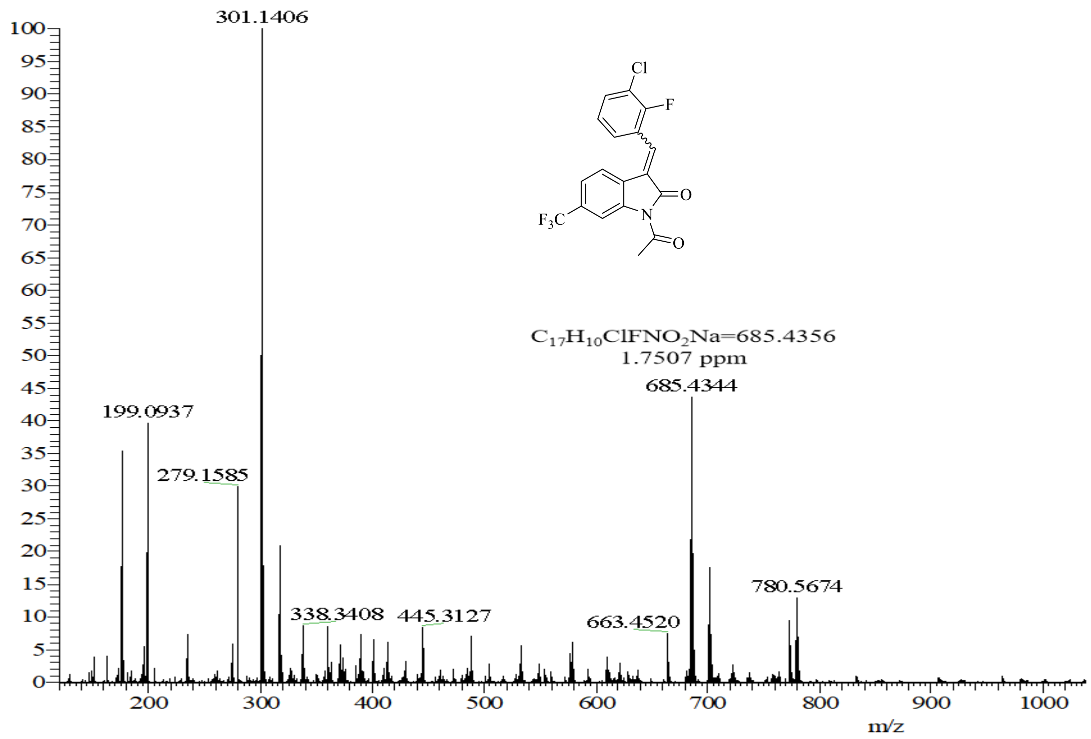

Figure S16-3. HRMS of compound **4p**

1-Acetyl-6-chloro-3-(3-chloro-2-fluorobenzylidene)indolin-2-one(4q)

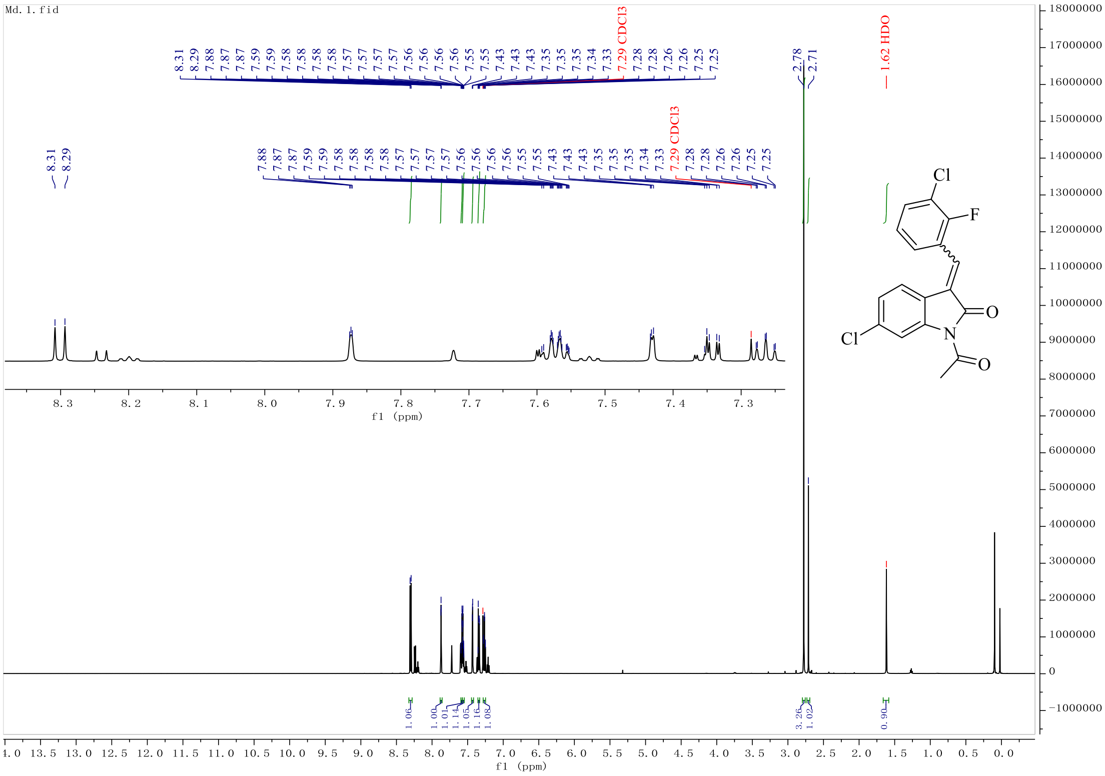

Figure S17-1. ^{1}H NMR spectrum (600 MHz, $CDCl_3$) of compound **4q**

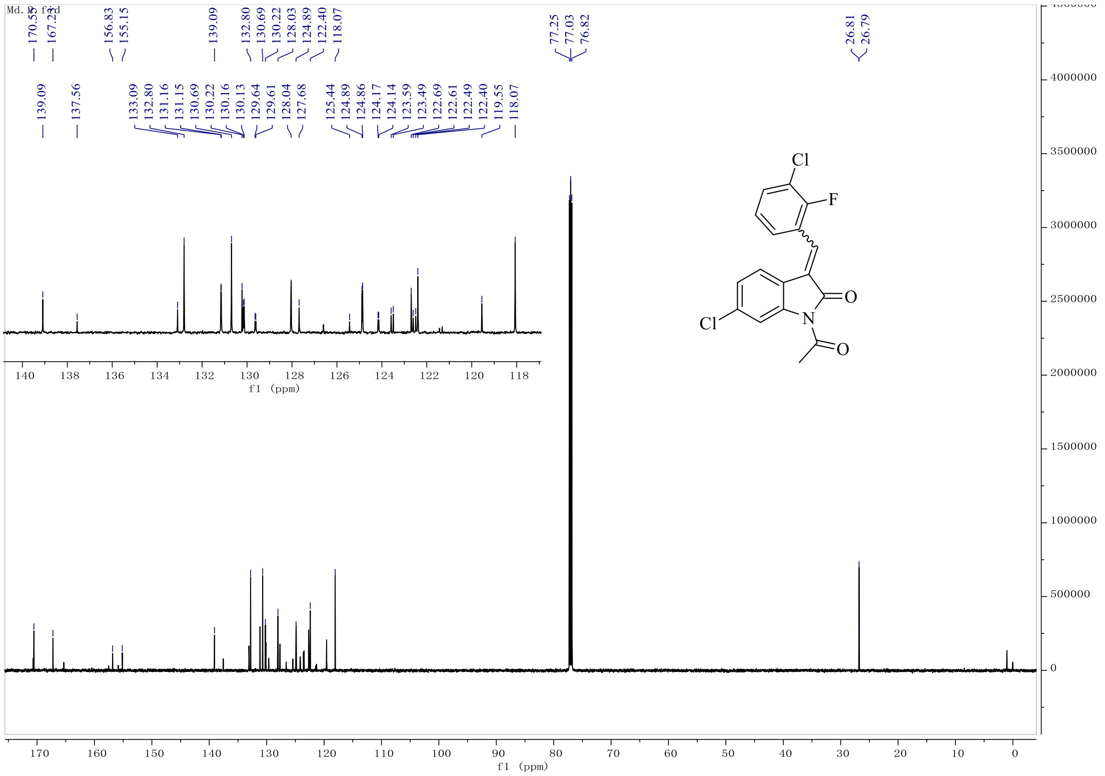

Figure S17-2. ^{13}C NMR spectrum (150 MHz, $CDCl_3$) of compound **4q**

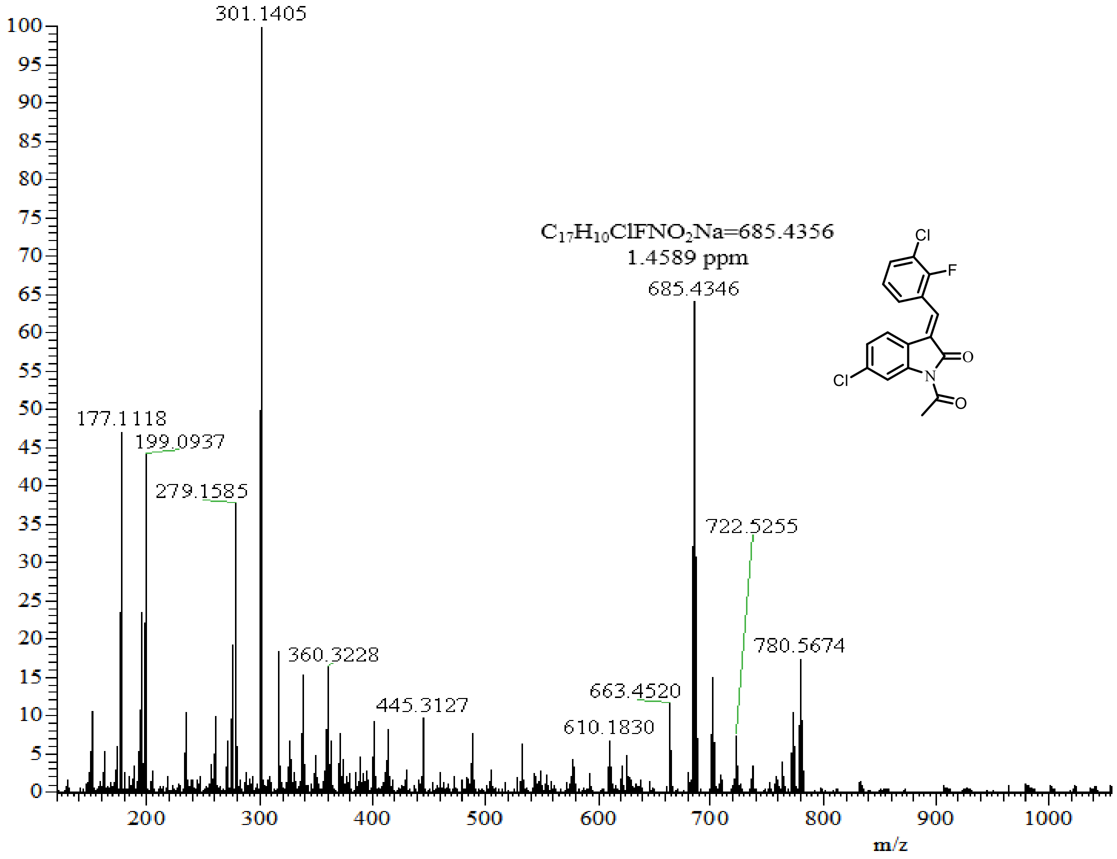

Figure S17-3. HRMS of compound **4q**

1-Acetyl-6-chloro-3-(3-methoxy-5-(trifluoromethyl)benzylidene)indolin-2-one(4r)

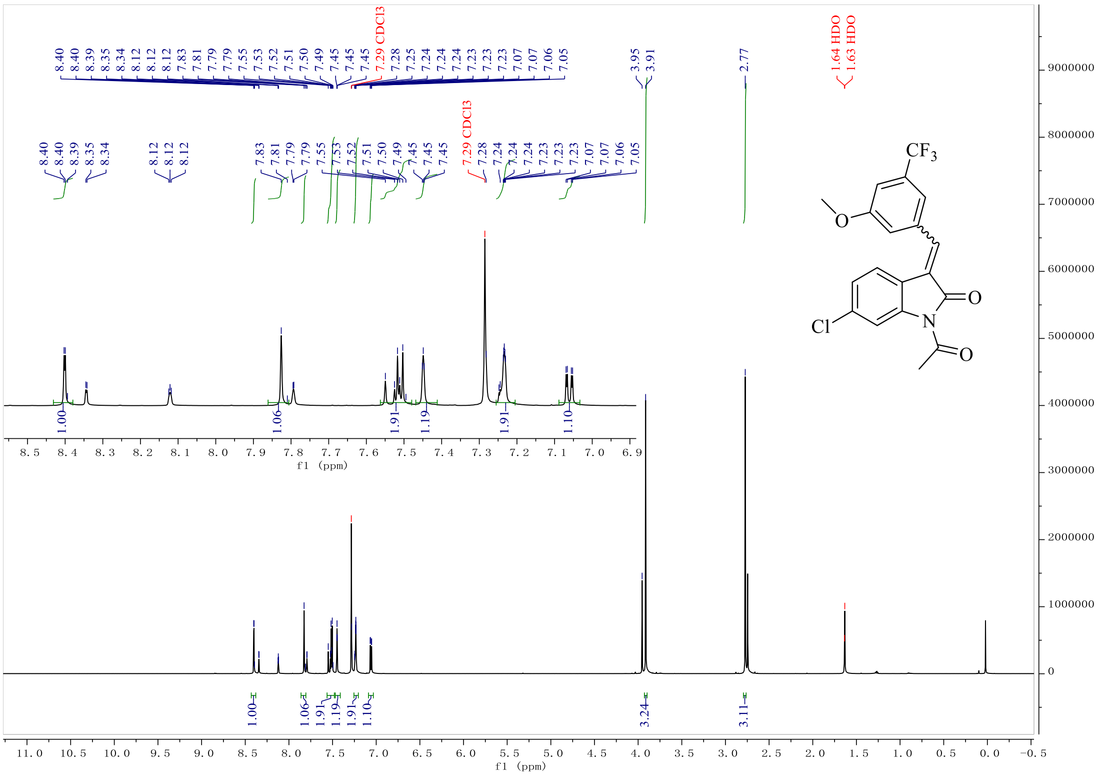

Figure S18-1. ^{1}H NMR spectrum (600 MHz, $CDCl_3$) of compound **4r**

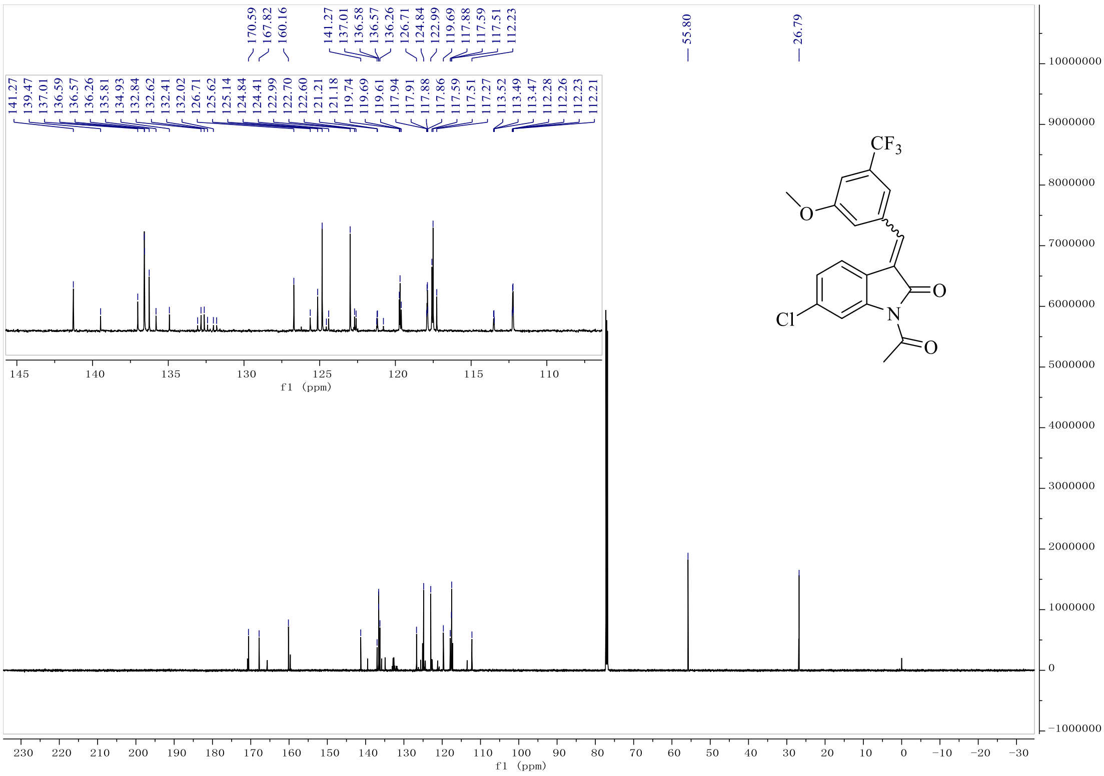

Figure S18-2. ^{13}C NMR spectrum (150 MHz, $CDCl_3$) of compound **4r**

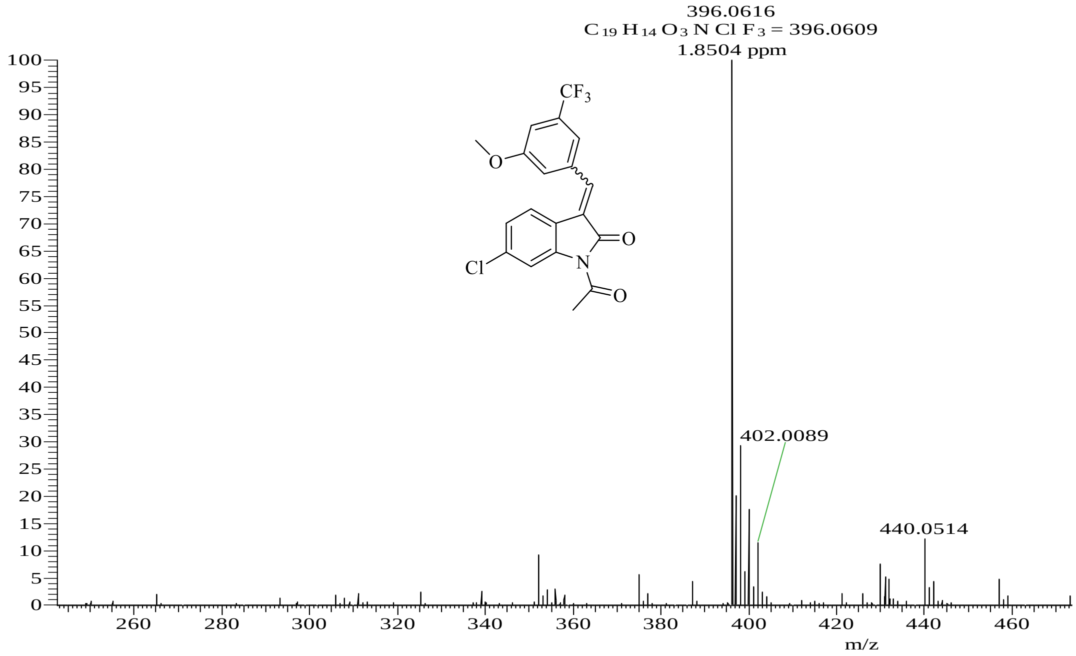

Figure S18-3. HRMS of compound **4r**

1-Acetyl-6-chloro-3-(3-methoxybenzylidene)indolin-2-one(4s)

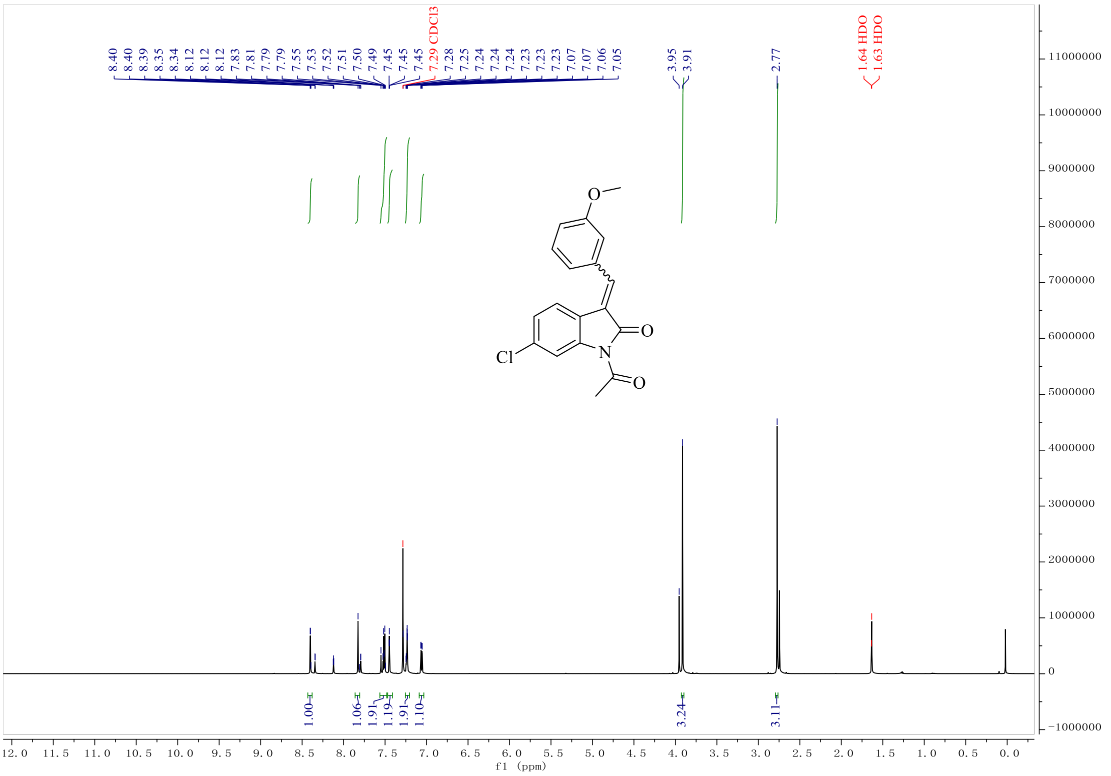

Figure S19-1. ^{1}H NMR spectrum (600 MHz, 150 MHz, $CDCl_3$) of compound **4s**

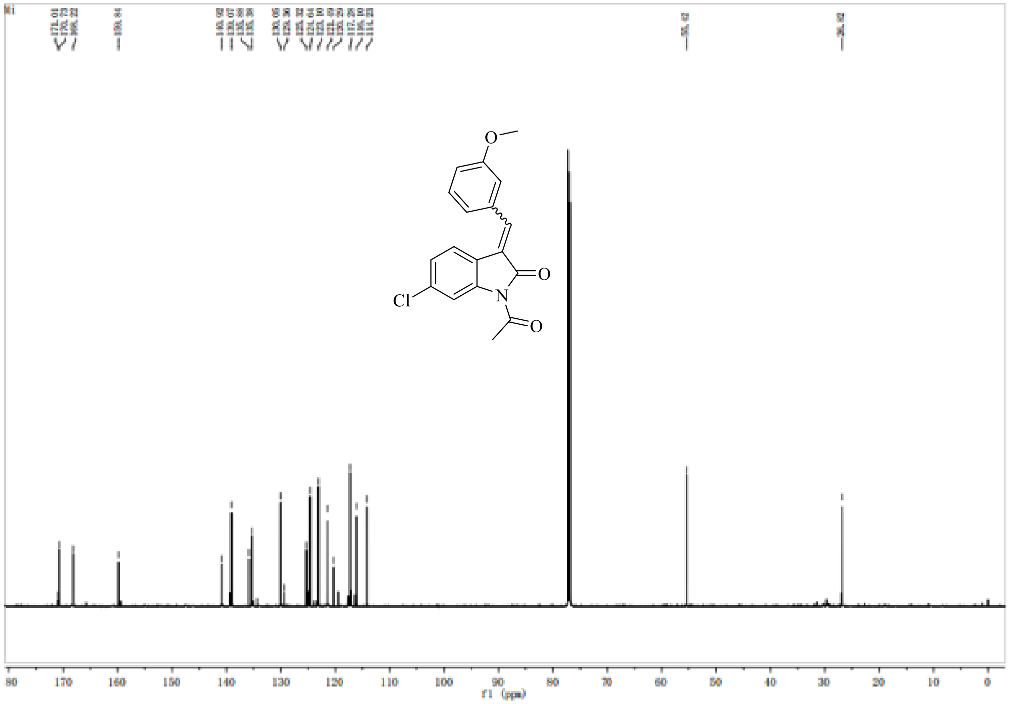

Figure S19-2. ^{13}C NMR spectrum (150 MHz, $CDCl_3$) of compound **4s**

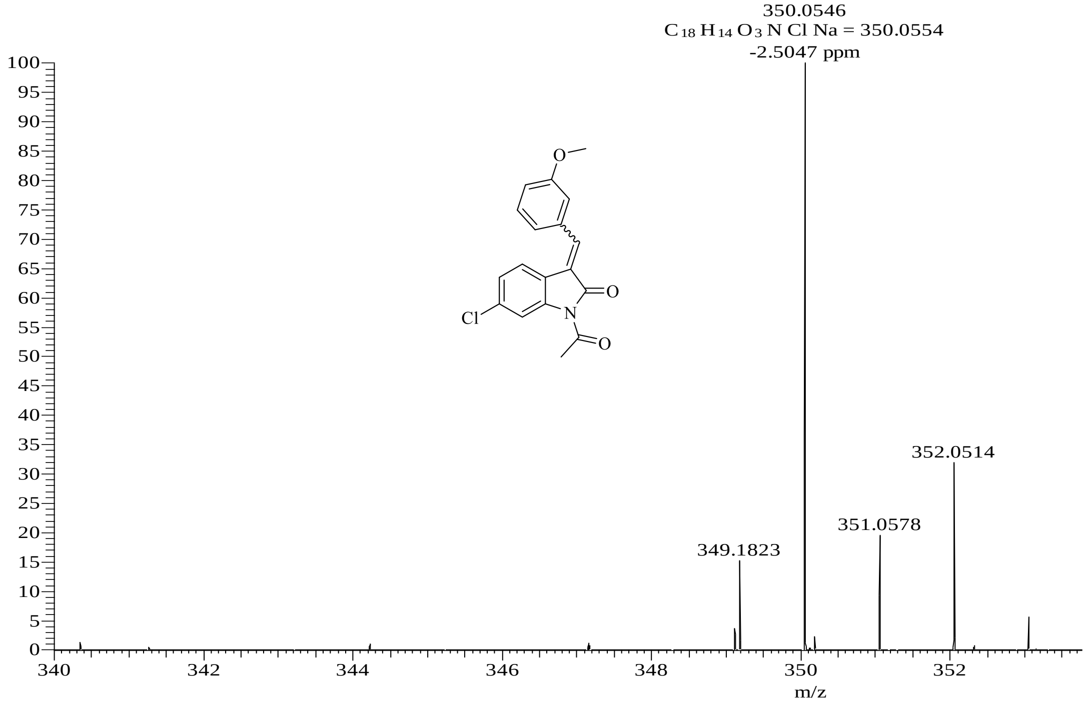

Figure S19-3. HRMS of compound **4s**

Tert-buty-6-chloro-2-oxo-3-(3-(trifluoromethyl)benzylidene)indoline-1-carboxylate(4t)

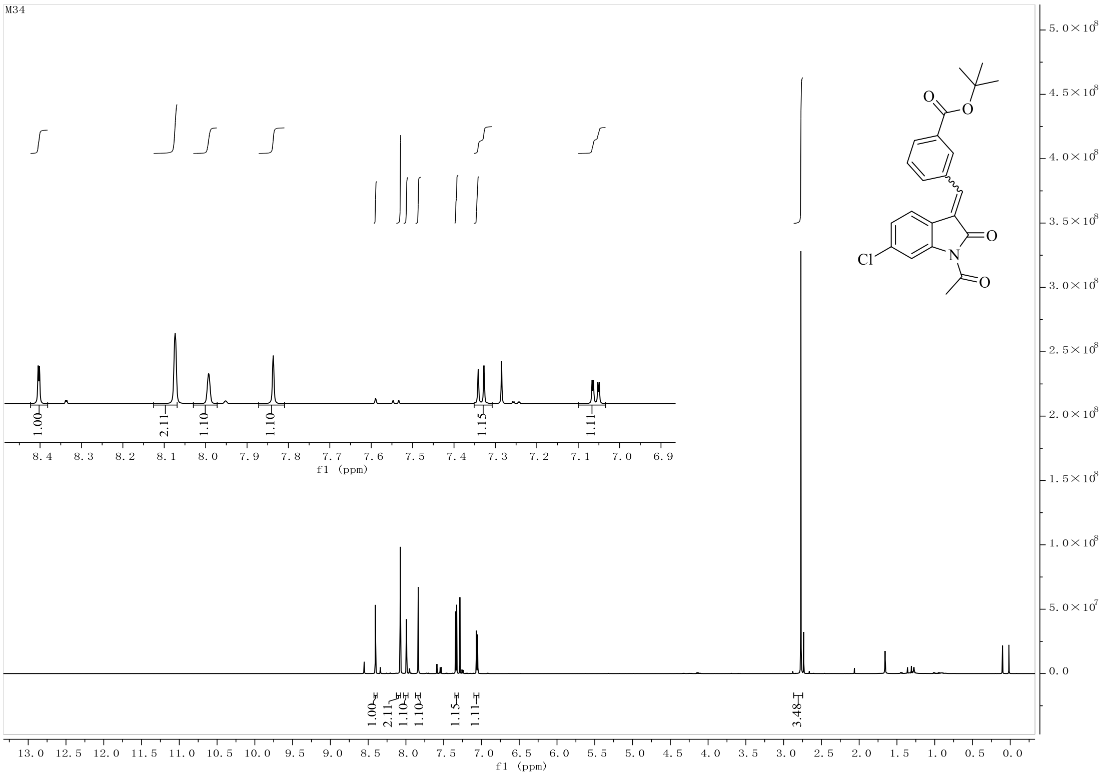

Figure S20-1. ^{1}H NMR spectrum (600 MHz, $CDCl_3$) of compound **4t**

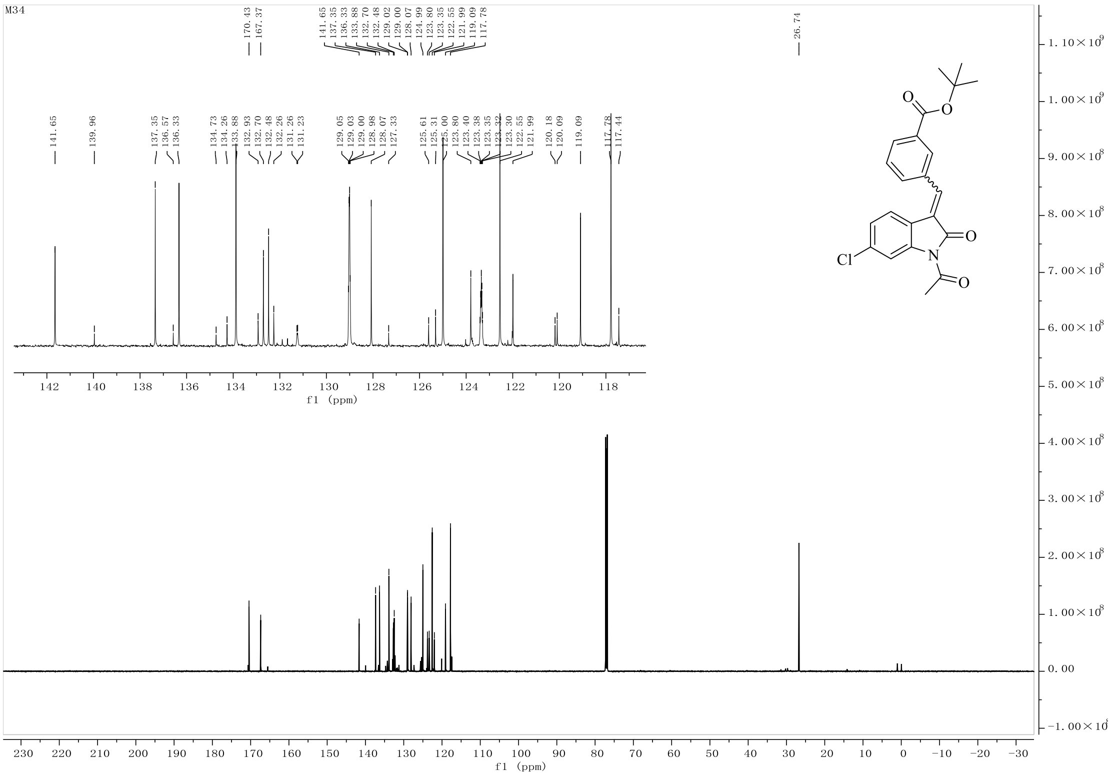

Figure S20-2. ^{13}C NMR spectrum (150 MHz, $CDCl_3$) of compound **4t**

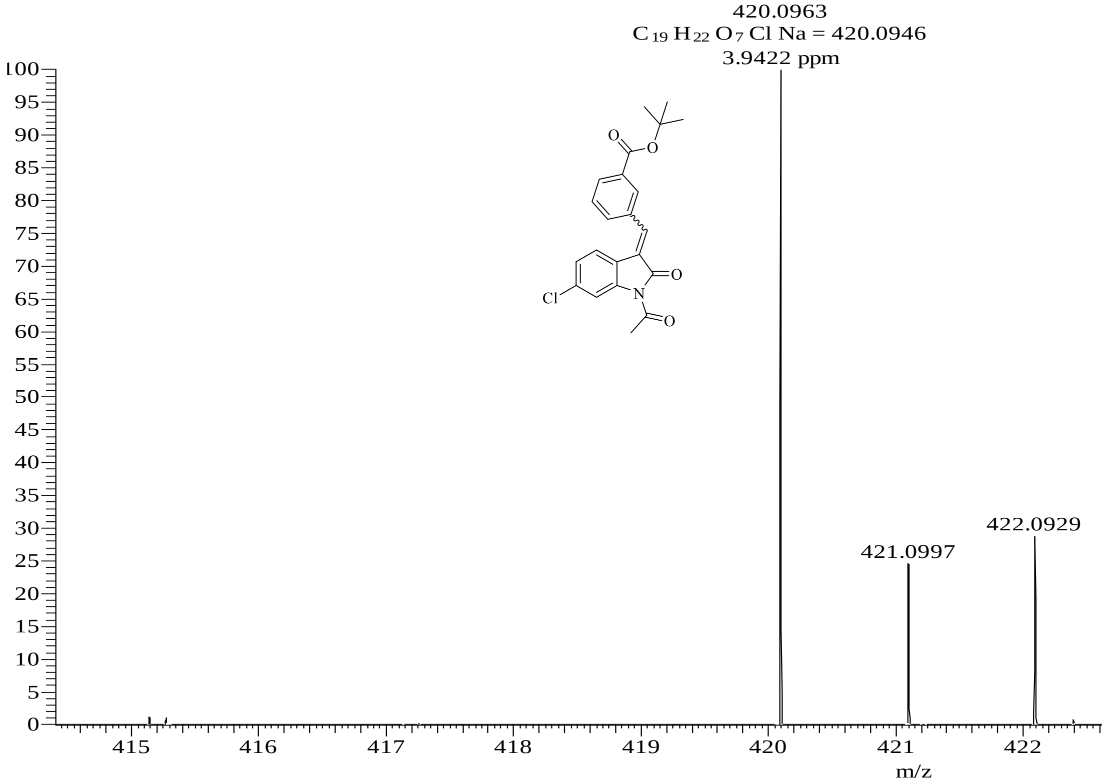

Figure S20-2. HRMS of compound **4t**

1-Acetyl-6-chloro-3-(3-(trifluoromethoxy)benzylidene)indolin-2-one(4u)

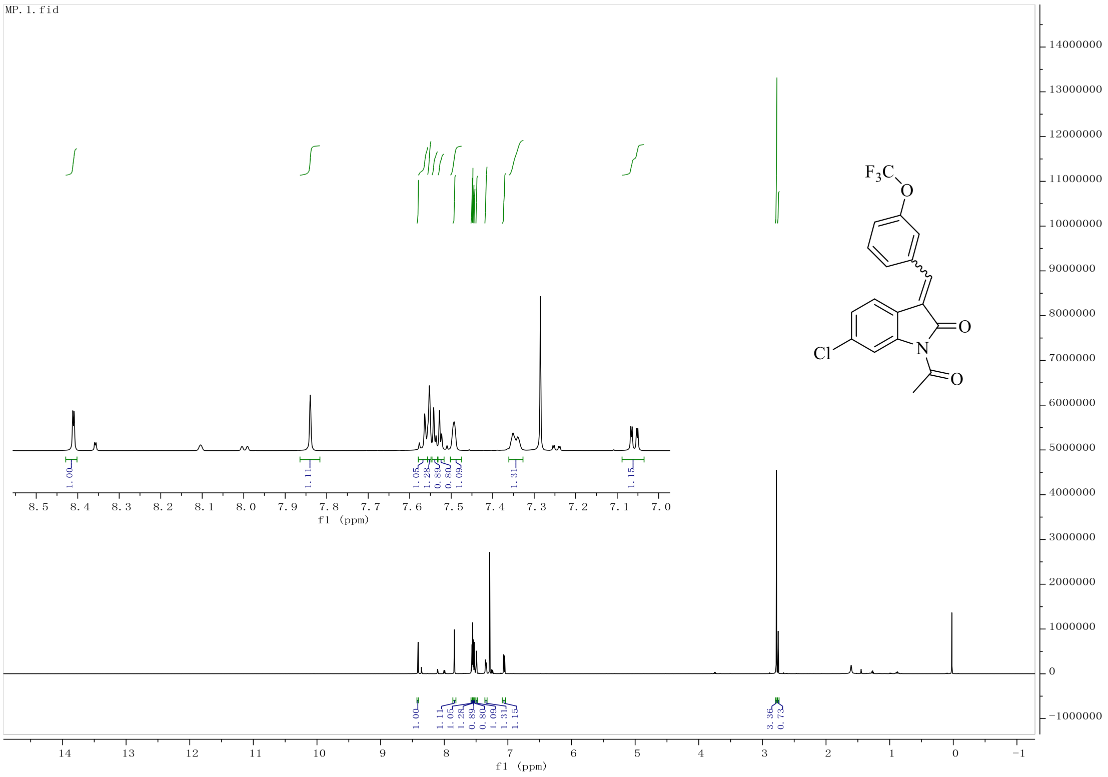

Figure S21-1. ^{1}H NMR spectrum (600 MHz, $CDCl_3$) of compound **4u**

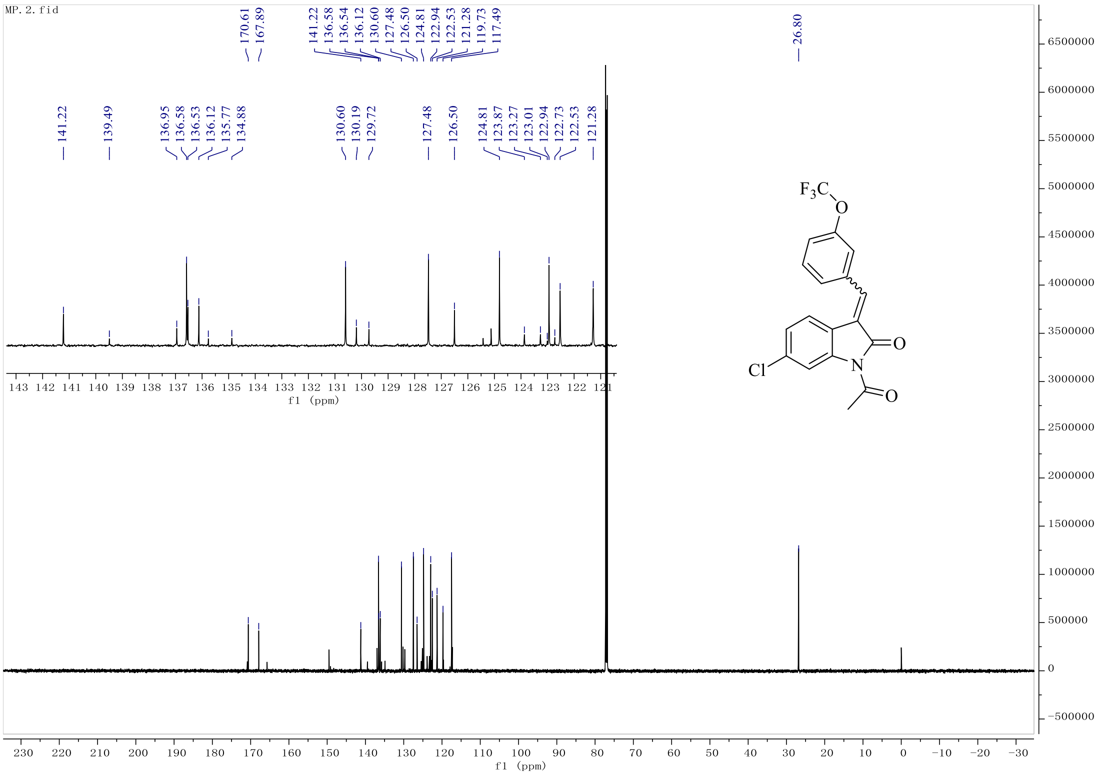

Figure S21-2. ^{13}C NMR spectrum (150 HMz, $CDCl_3$) of compound **4u**

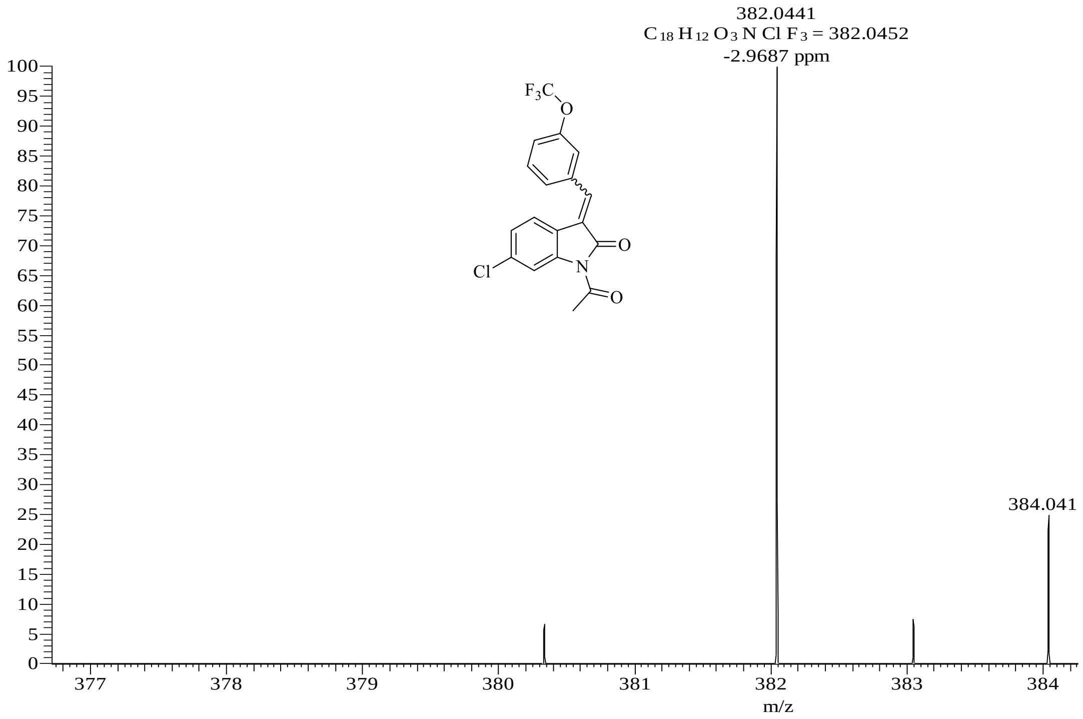

Figure S21-3. HRMS of compound **4u**

1-Acetyl-6-chloro-3-(pyridin-4-ylmethylene)indolin-2-one(4v)

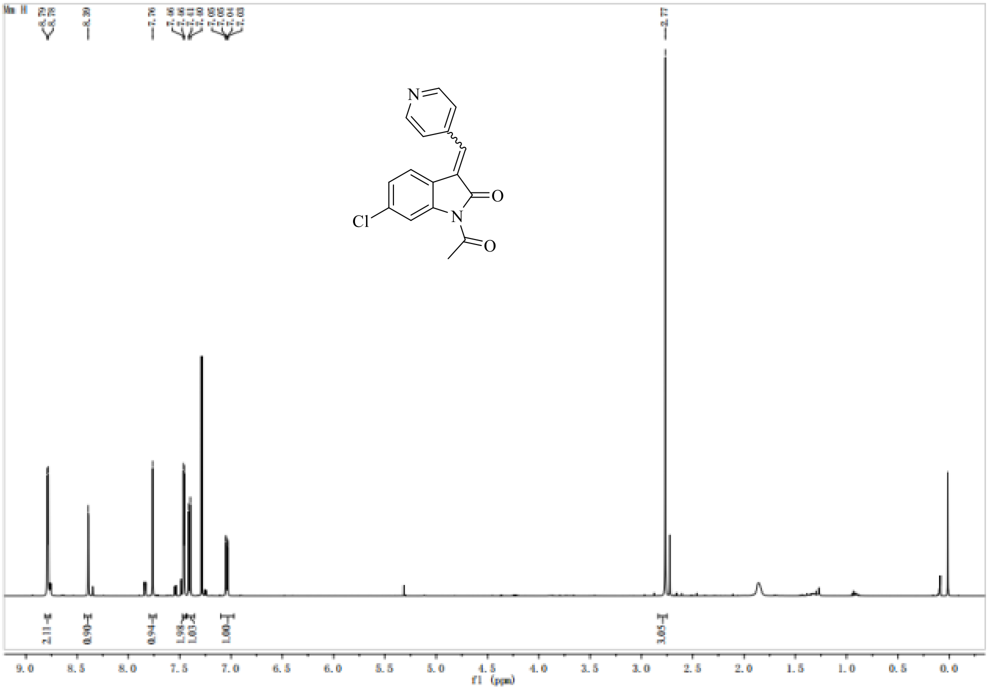

Figure S22-1. ^{1}H NMR spectrum (600 MHz, $CDCl_3$) of compound **4v**

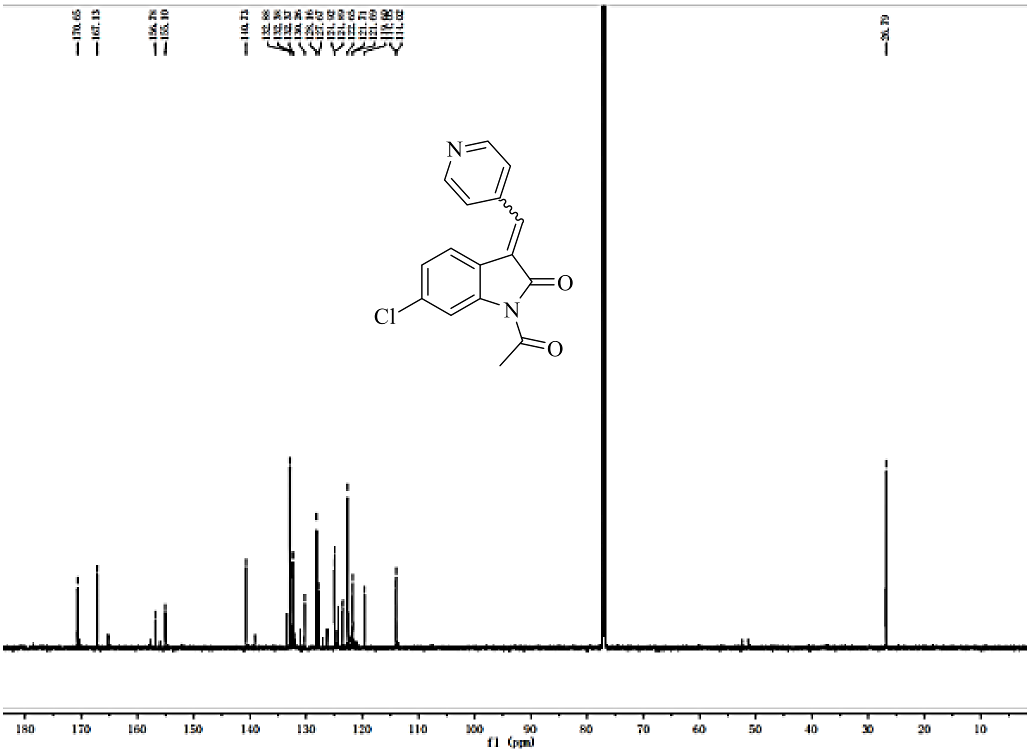

Figure S22-2. ^{13}C NMR spectrum (150 MHz, $CDCl_3$) of compound **4v**

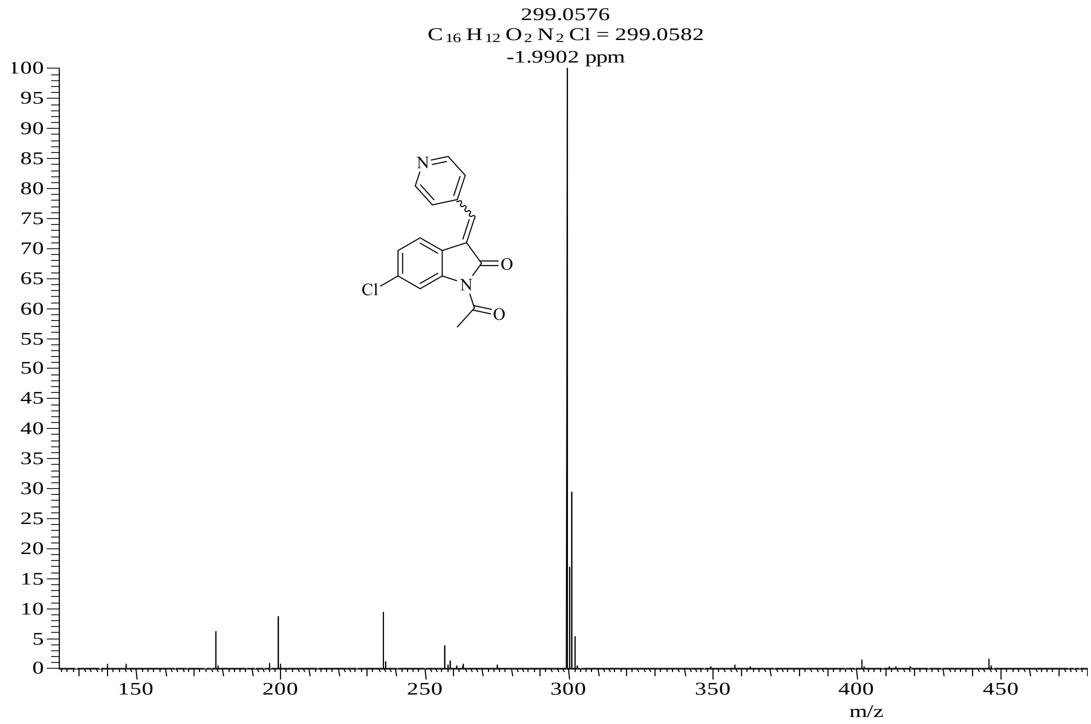

Figure S22-3. HRMS of compound **4v**

1-Acetyl-6-chloro-3-((2-(trifluoromethyl)pyridin-4-yl)methylene)indolin-2-one(4w)

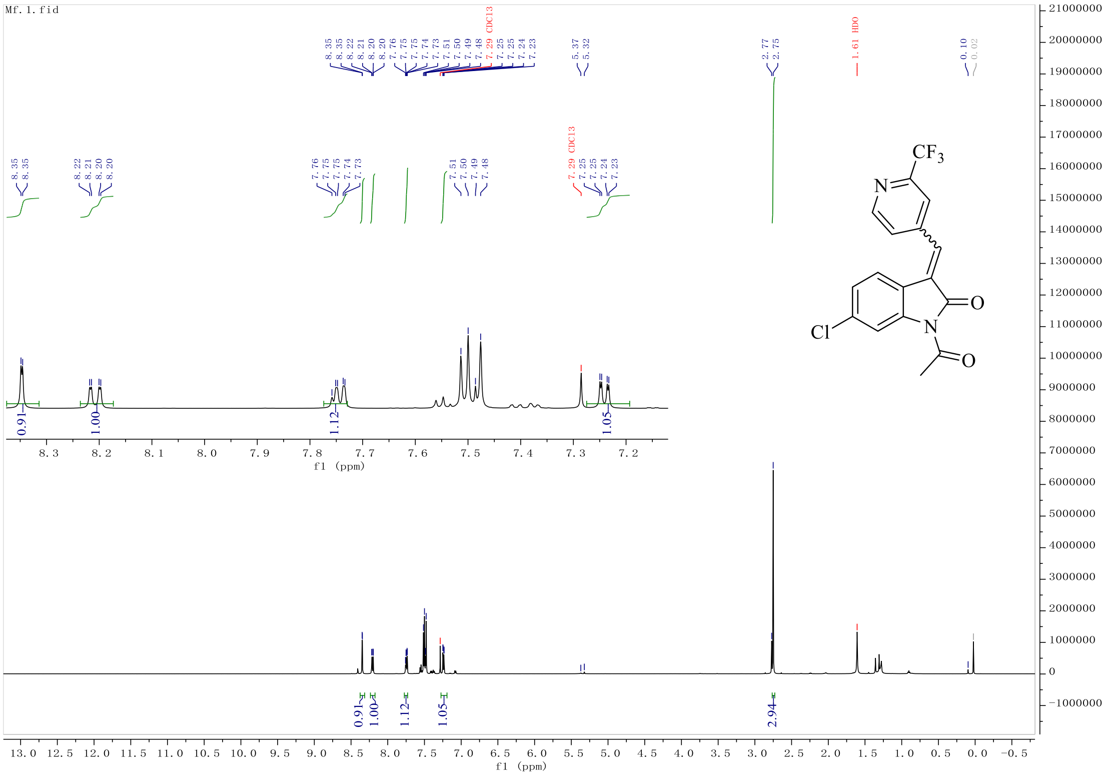

Figure S23-1. ¹H NMR spectrum (600 MHz, $CDCl_3$) of compound **4w**

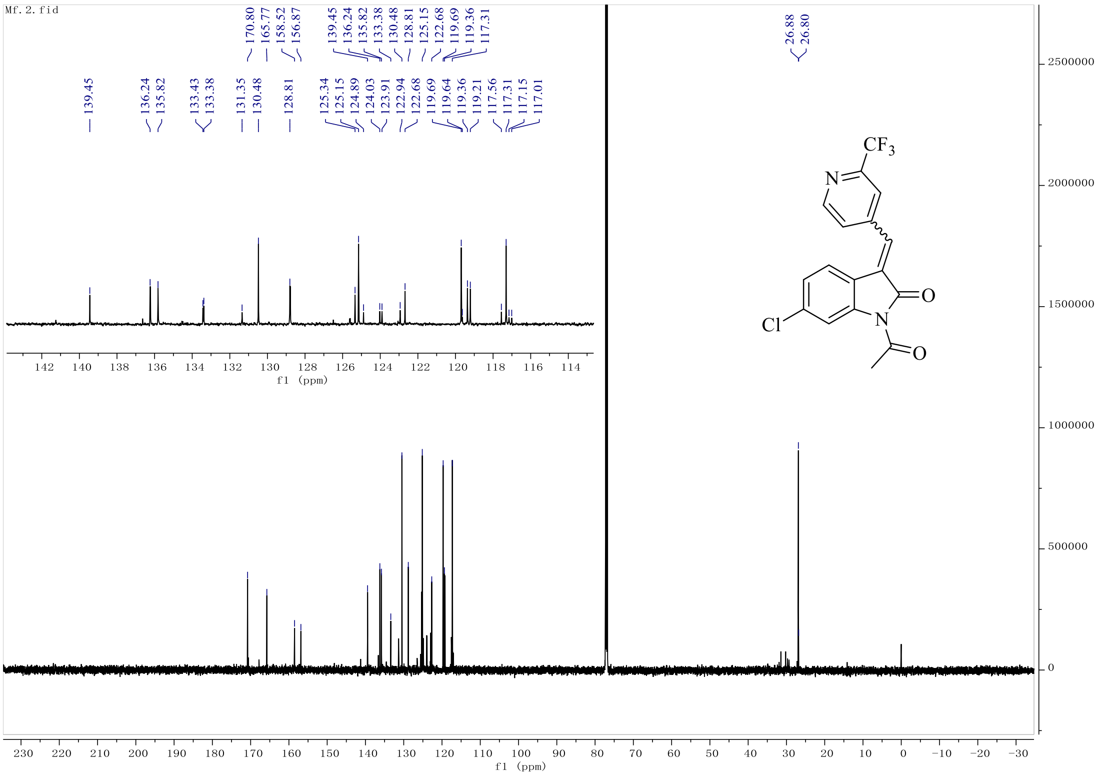

Figure S23-2. ¹³C NMR spectrum (150 MHz, $CDCl_3$) of compound **4w**

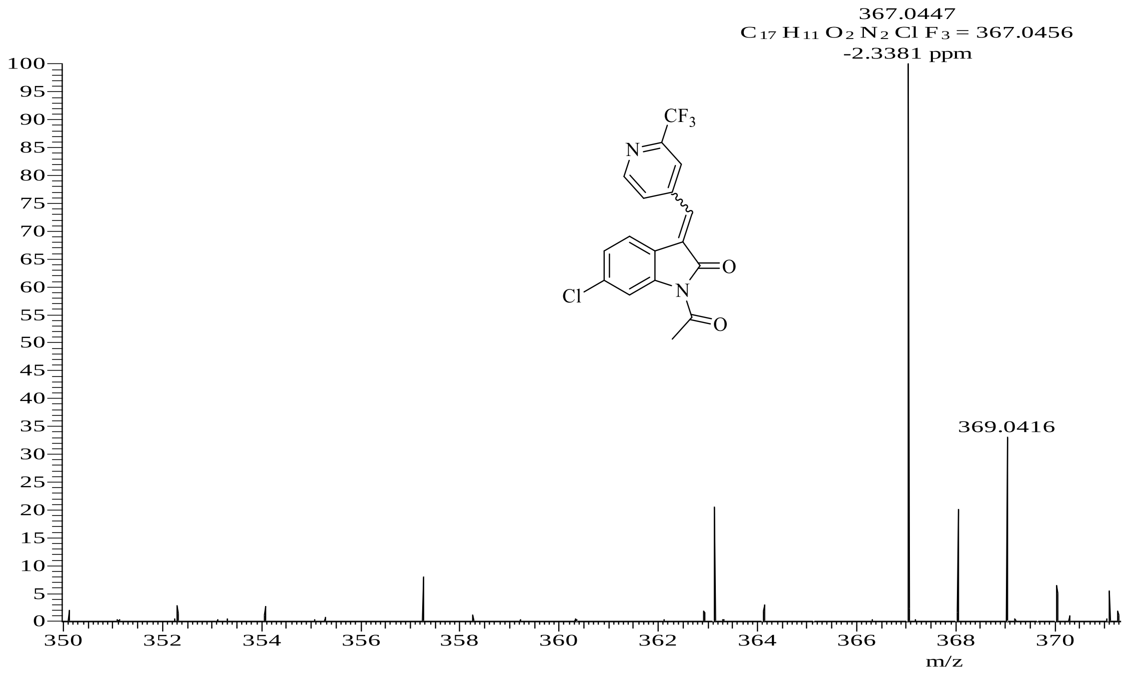

Figure S23-3. HRMS of compound **4w**

***Tert*-butyl -4-((1-acetyl-6-chloro-2-oxoindolin-3-ylidene)methyl)benzoate(4x)**

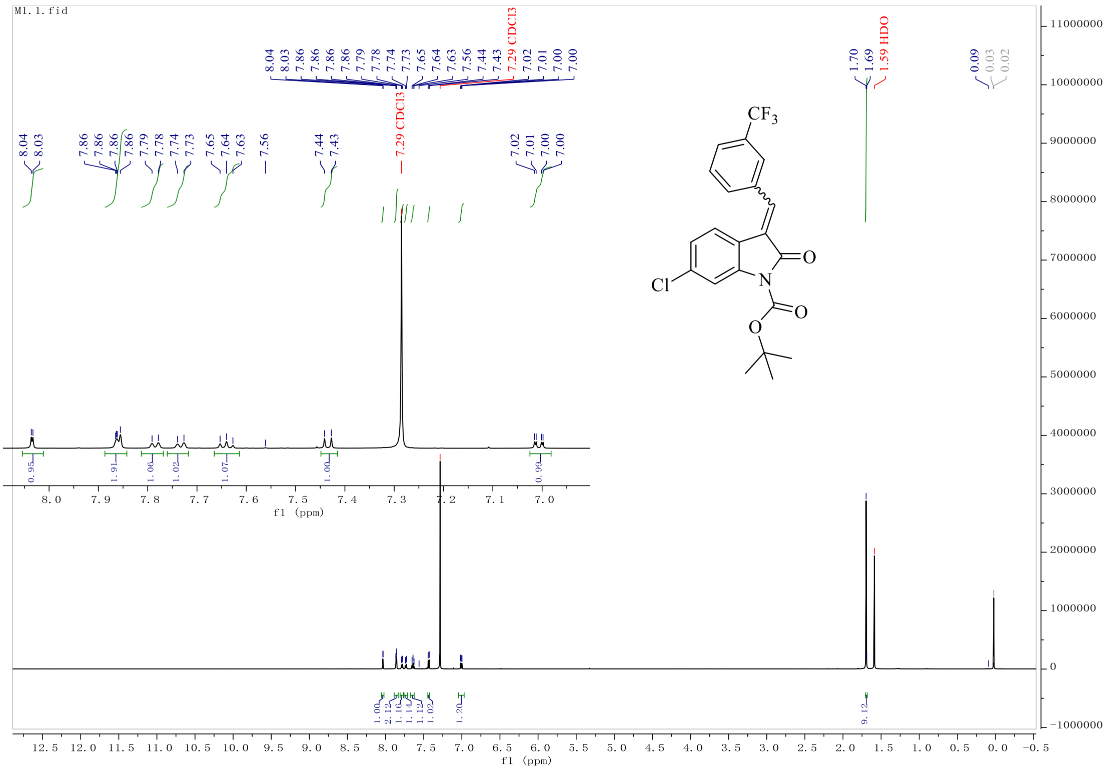

Figure S24-1. ^{1}H NMR spectrum (600 MHz, CDCl$_3$) of compound **4x**

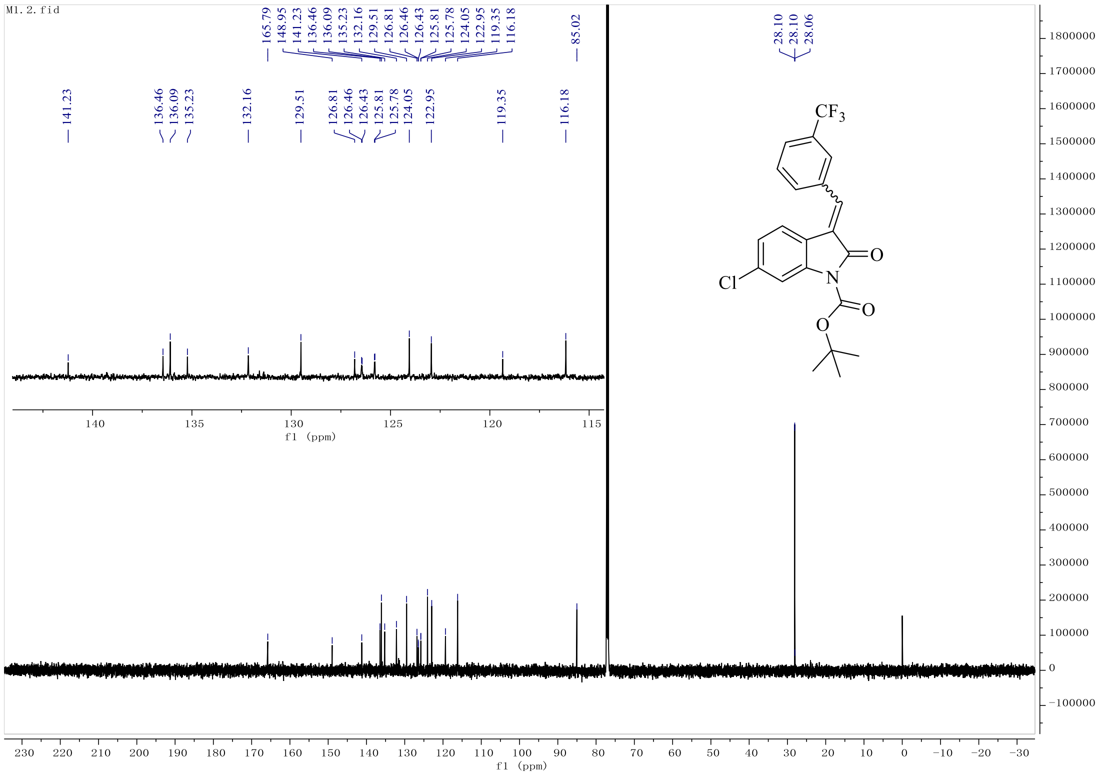

Figure S24-2. ^{13}C NMR spectrum (150 MHz, CDCl$_3$) of compound **4x**

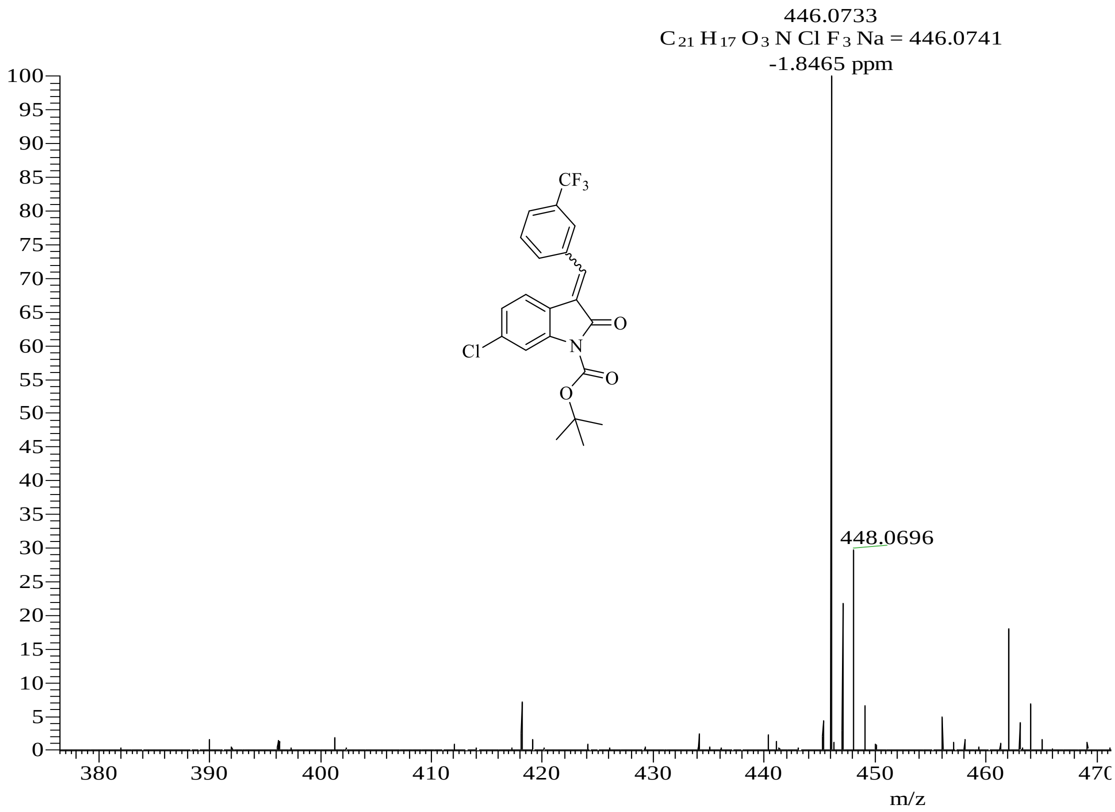

Figure S24-3. HRMS of compound **4x**

4-Nitro-N-(2-oxo-3-(3-(trifluoromethyl)benzylidene)indolin-5-yl)benzenesulfonamide (9d)

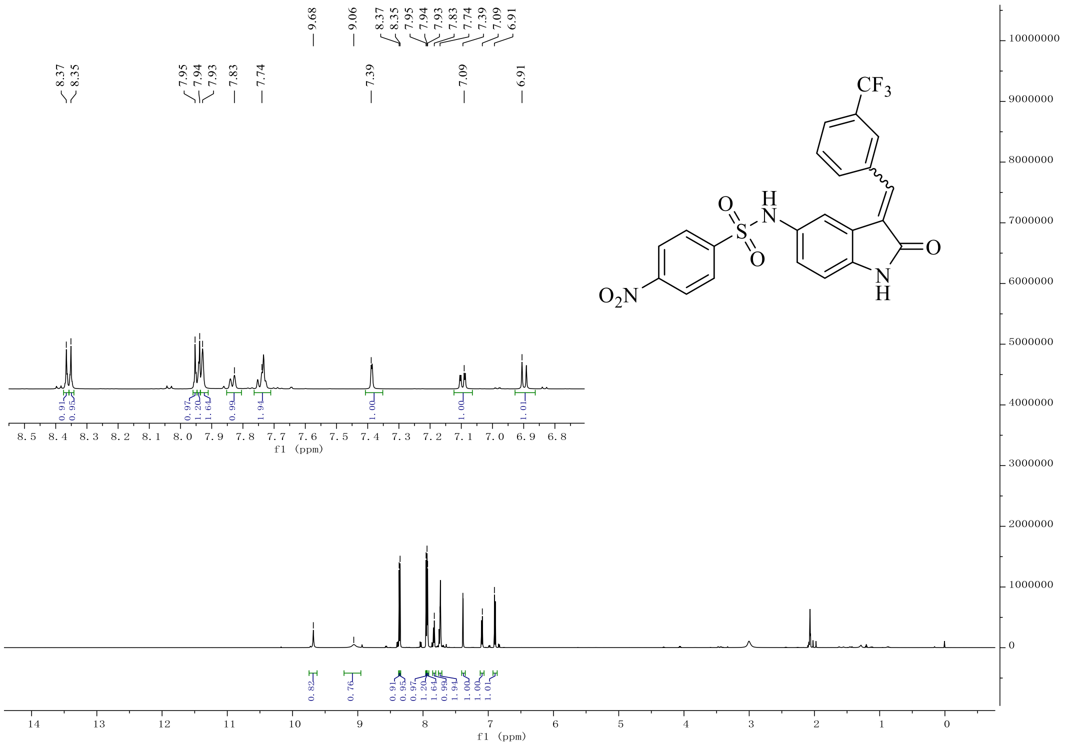

Figure S25-1. ^{1}H NMR spectrum (600 Hz, Acetone) of compound **9d**

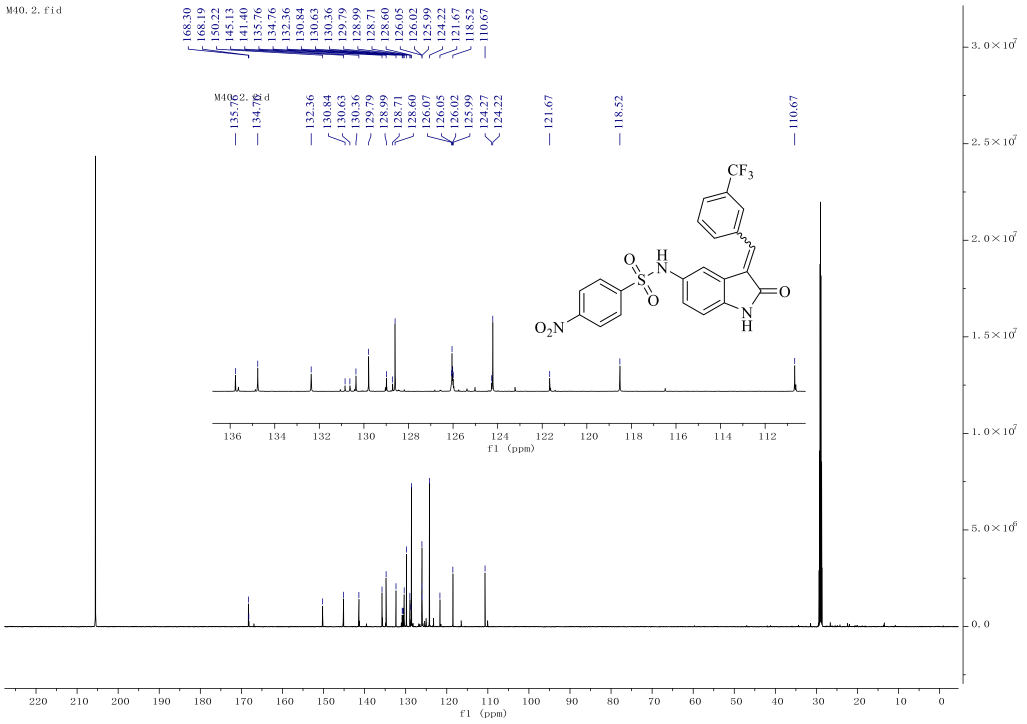

Figure S25-2. ^{13}C NMR spectrum (150 Hz, Acetone) of compound **9d**

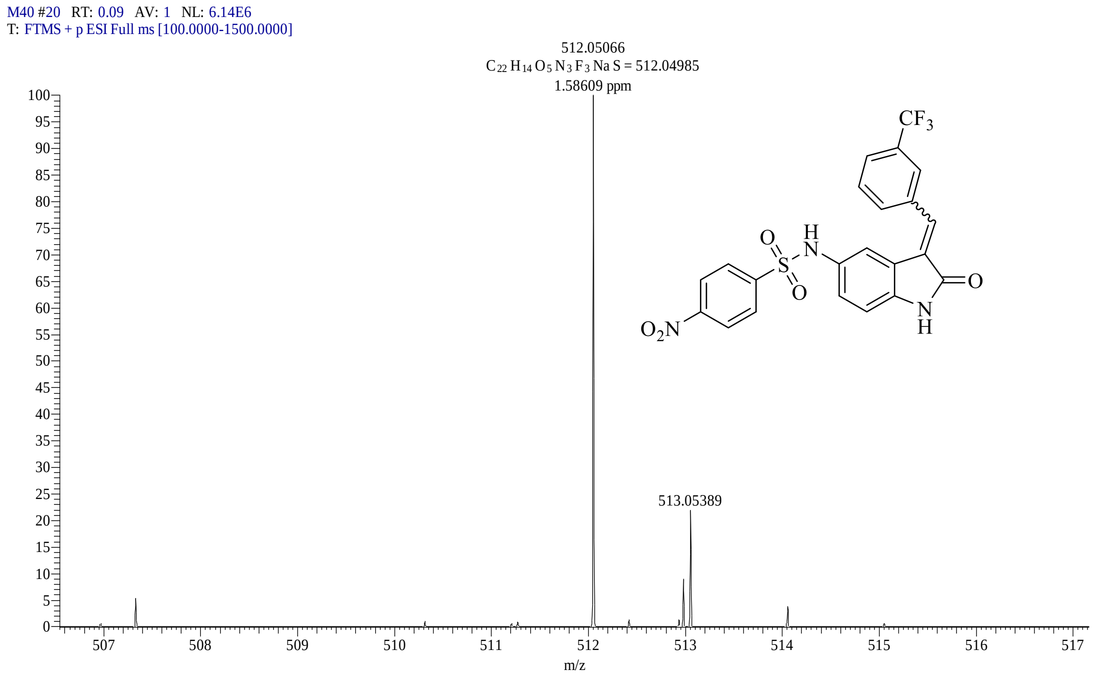

Figure S25-3. HRMS of compound **9d**

nitro-N-(2-oxo-3-(3-(trifluoromethyl)benzylidene)indolin-5-yl)benzenesulfonamide(**9a**)

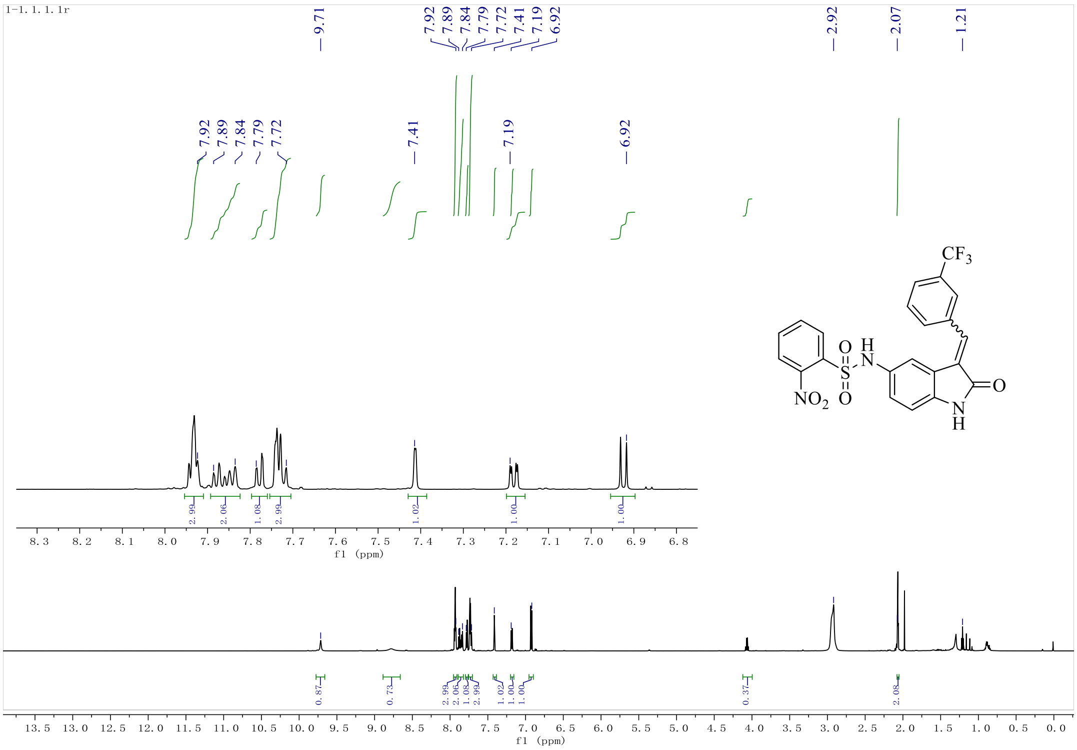

Figure S26-1. ^{1}H NMR spectrum (600 Hz, CD3COCD3) of compound **9a**

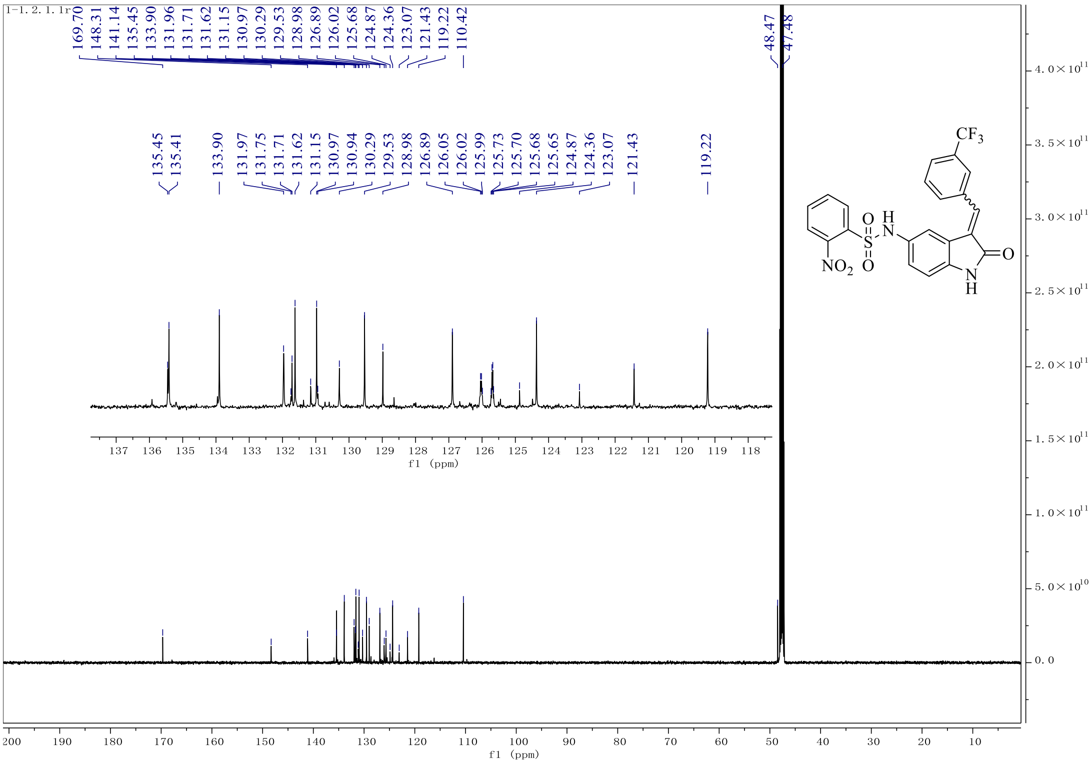

Figure S26-2. ^{13}C NMR spectrum (150 Hz, CD3OD) of compound **9a**

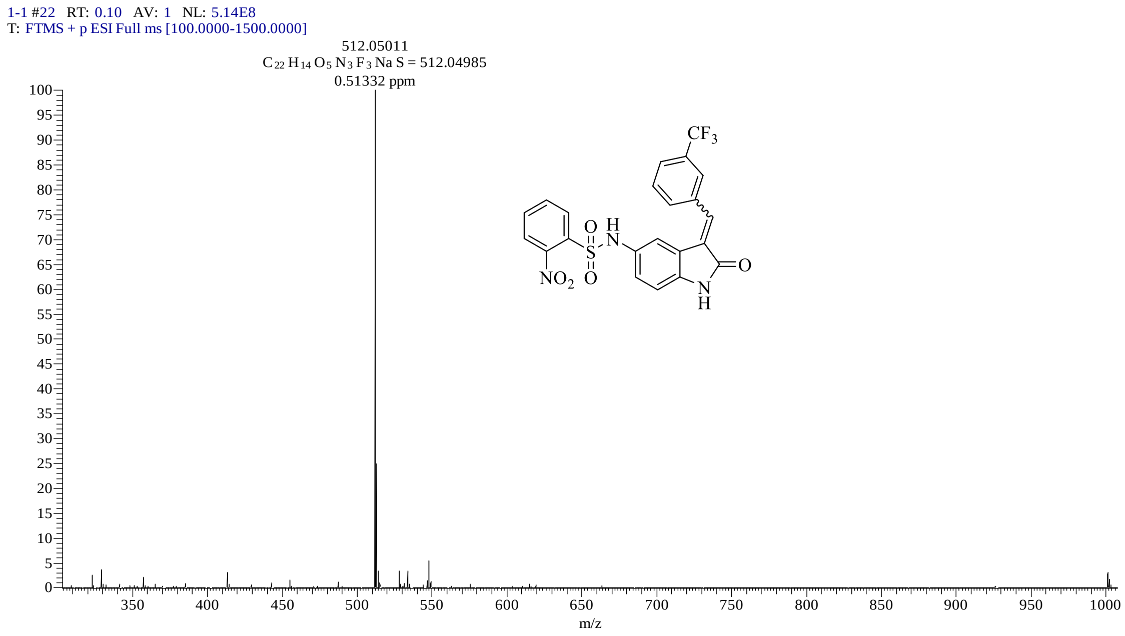

Figure S26-3. HRMS of compound **9a**

2,4-Difluoro-*N*-(2-oxo-3-(3-(trifluoromethyl)benzylidene)indolin-5-yl)benzenesulfonamide (9b)

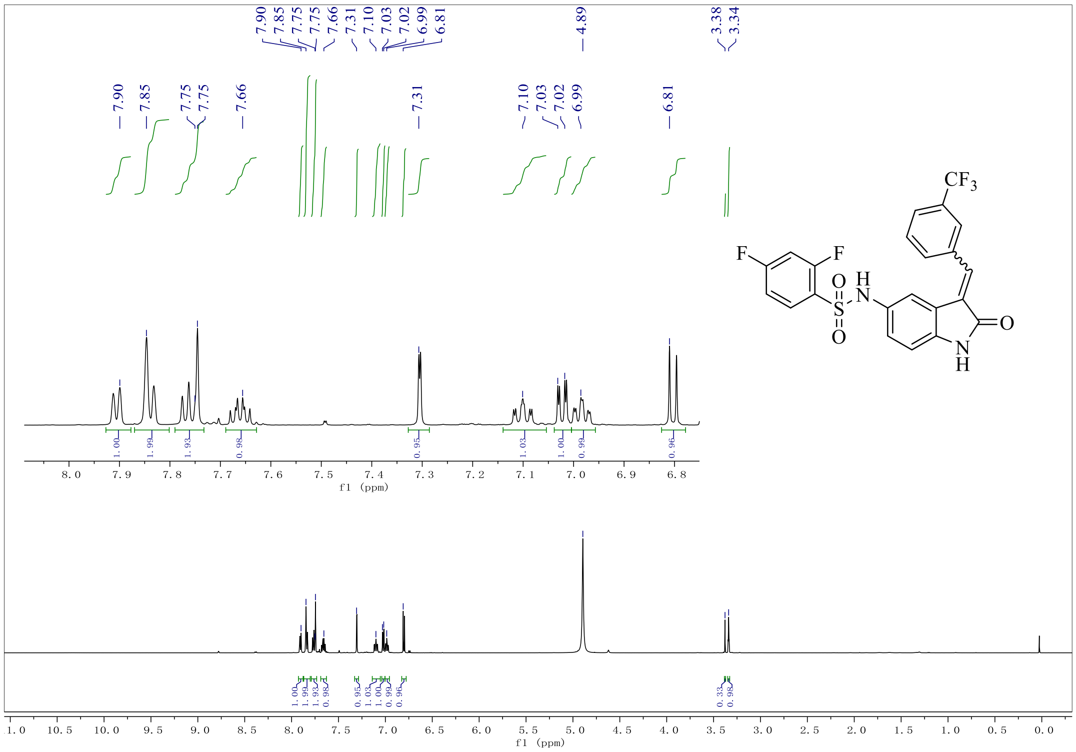

Figure S27-1. ^{1}H, NMR spectrum (150 Hz, CD_3OD) of compound **9b**

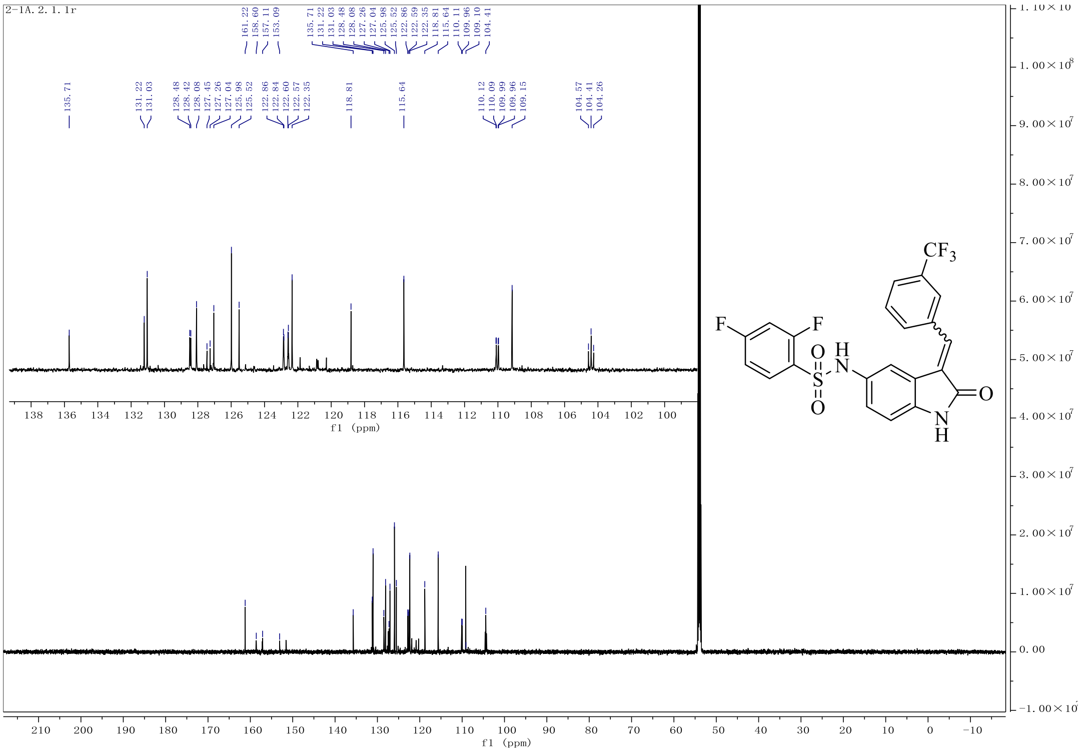

Figure S27-2. ^{13}C NMR spectrum (150 Hz, CD_3OD) of compound **9b**

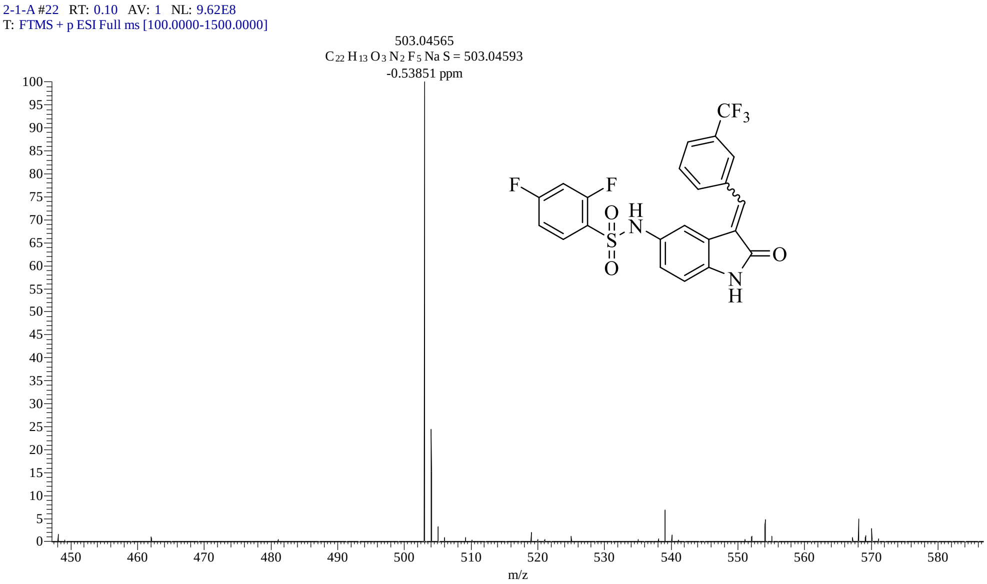

Figure S27-3. HRMS of compound **9b**

N-(2-oxo-3-(3-(trifluoromethyl)benzylidene)indolin-5-yl)ethanesulfonamide (9c)

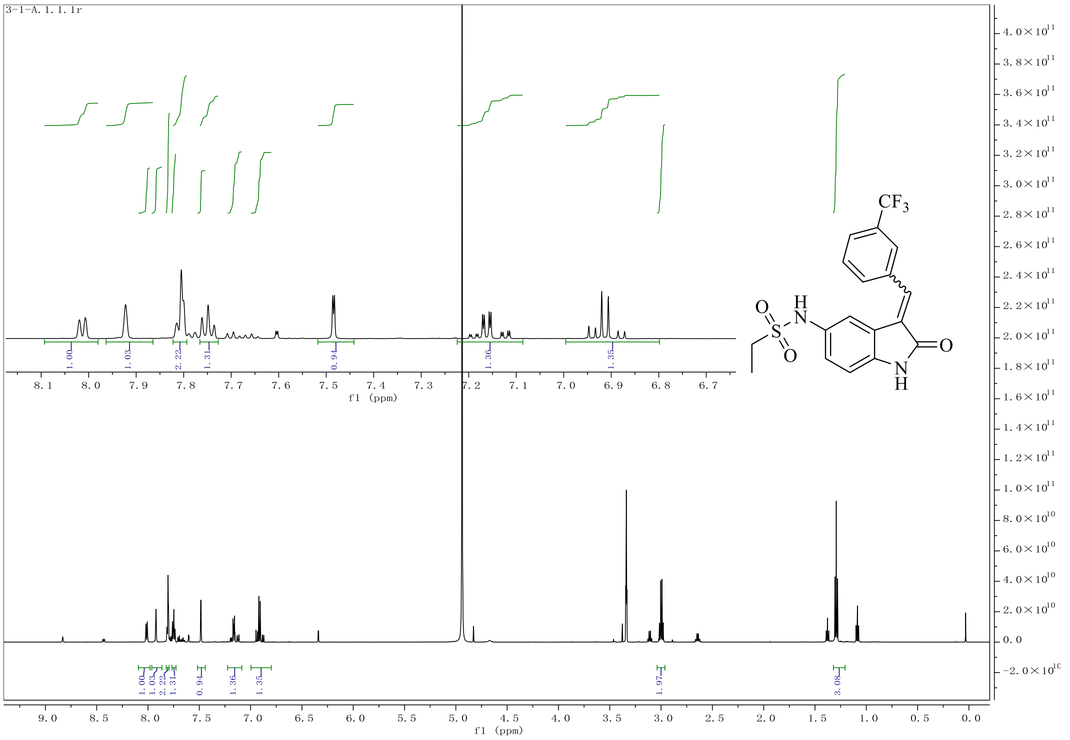

Figure S28-1. ^{1}H NMR spectrum (600 MHz, CD_3OCD_3) of compound **9c**

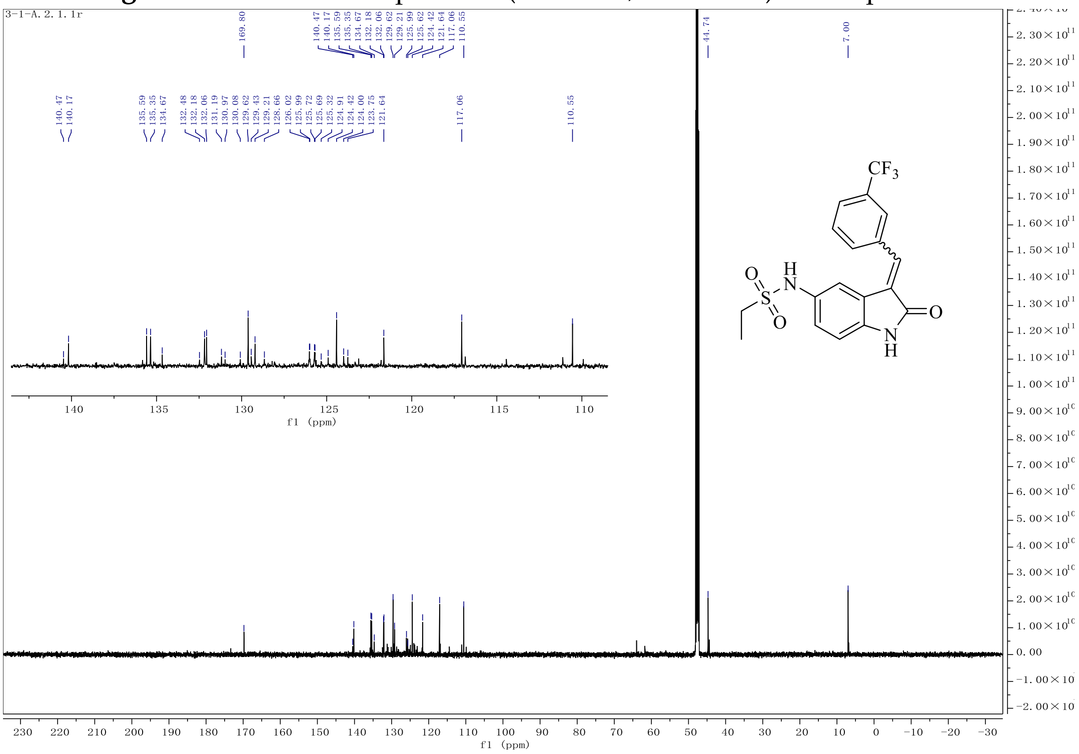

Figure S28-2. ^{13}C NMR spectrum (150 MHz, CD_3OCD_3) of compound **9c**

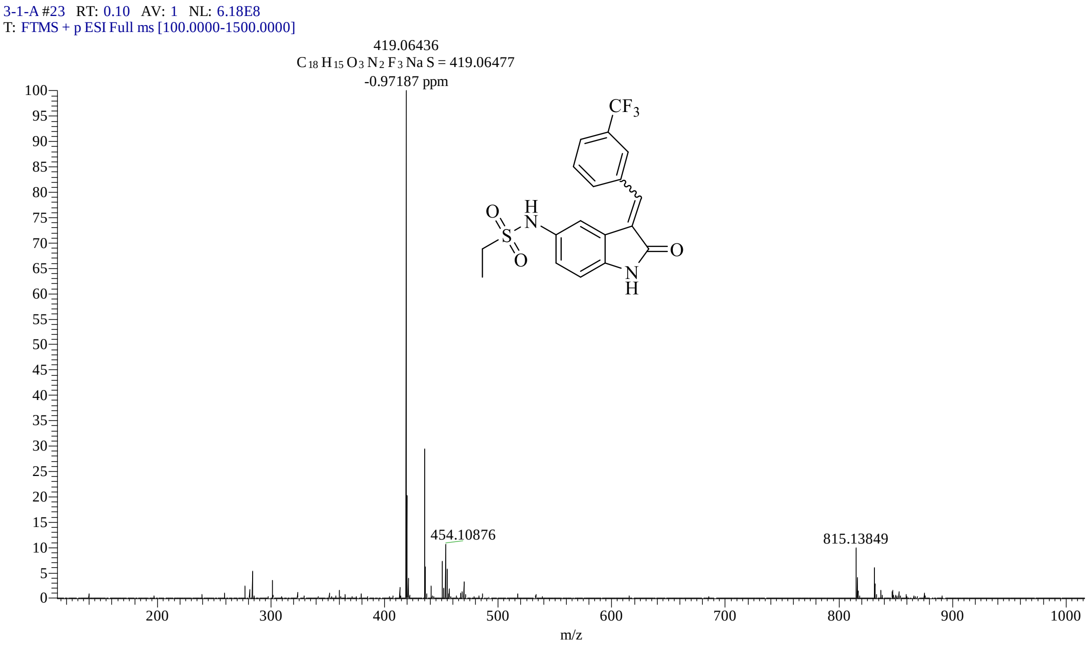

Figure S28-3. HRMS of compound **9c**

N-(2-oxo-3-(3-(trifluoromethyl)benzylidene)indolin-5-yl)-1-phenylmethanesulfonamide **(9e)**

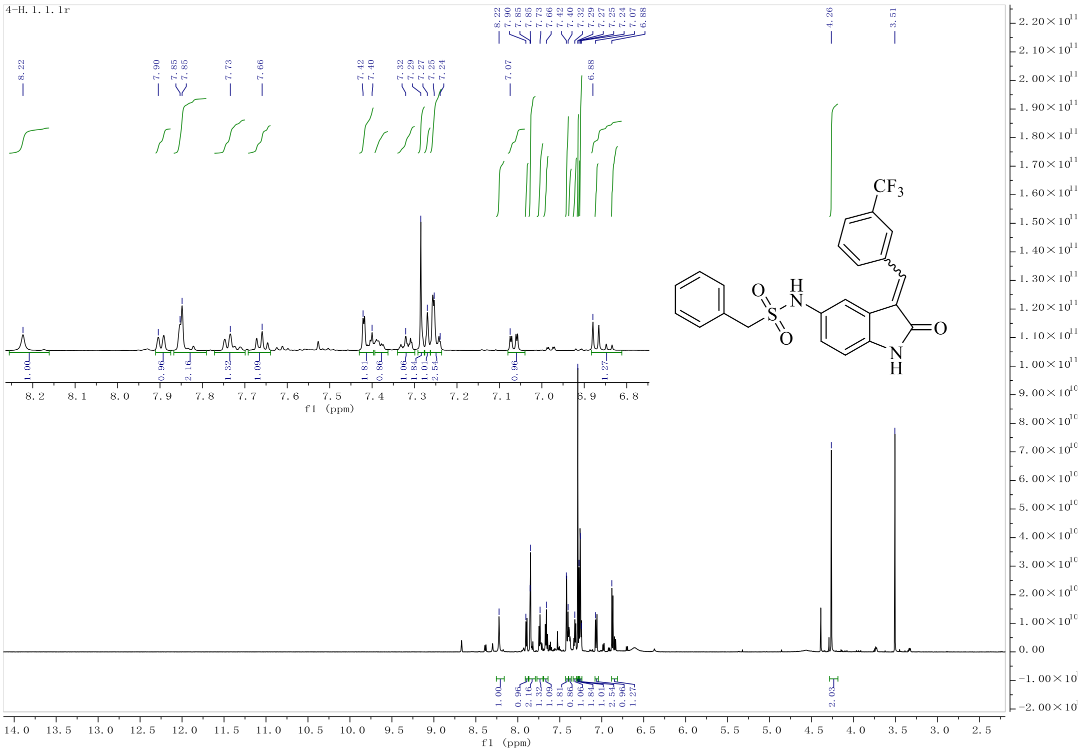

Figure S29-1. ^{1}H NMR spectrum (600 Hz, $CDCl_3$) of compound **9e**

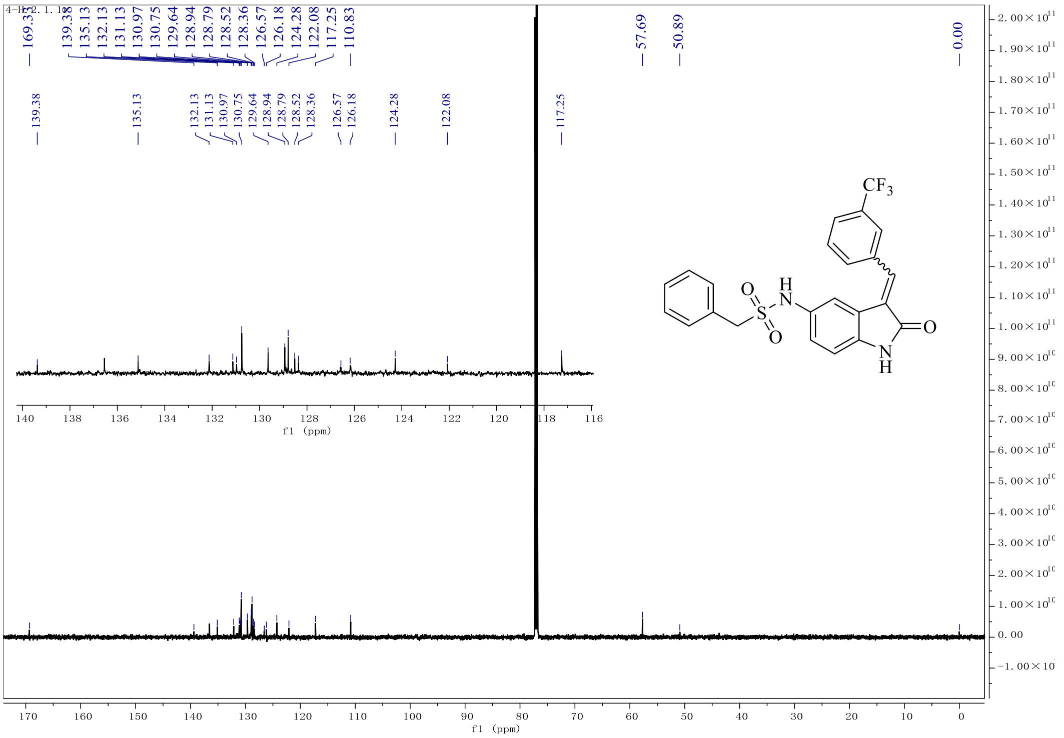

Figure S29-2. ^{13}C NMR spectrum (150 Hz, $CDCl_3$) of compound **9e**

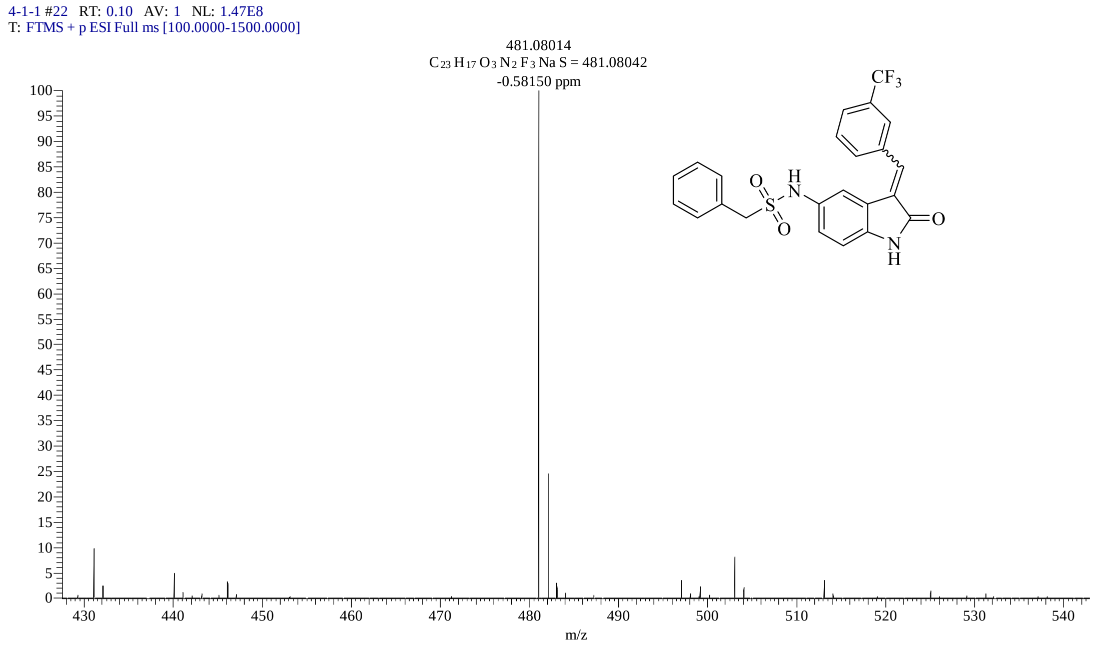

Figure S29-2. HRMS of compound **9e**

(Z)-4-methyl-N-(2-oxo-3-(3-(trifluoromethyl)benzylidene)indolin-5-yl)benzenesulfonamide (9f)

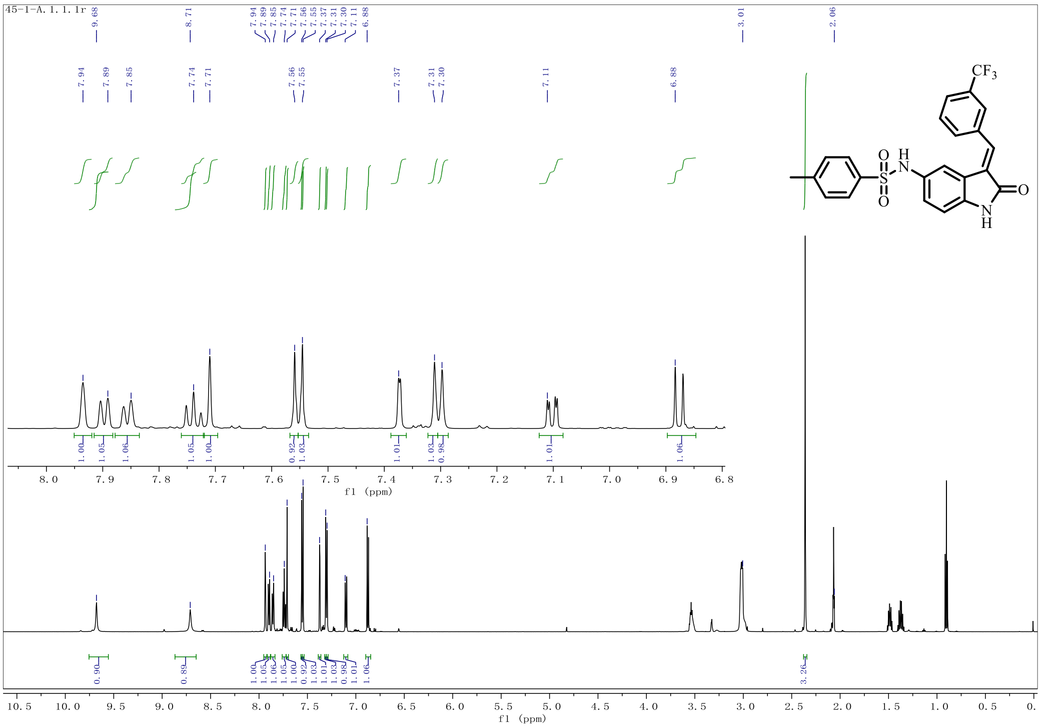

Figure S30-1. ^{1}H NMR spectrum (600 Hz, CD3OCD3) of compound **9f**

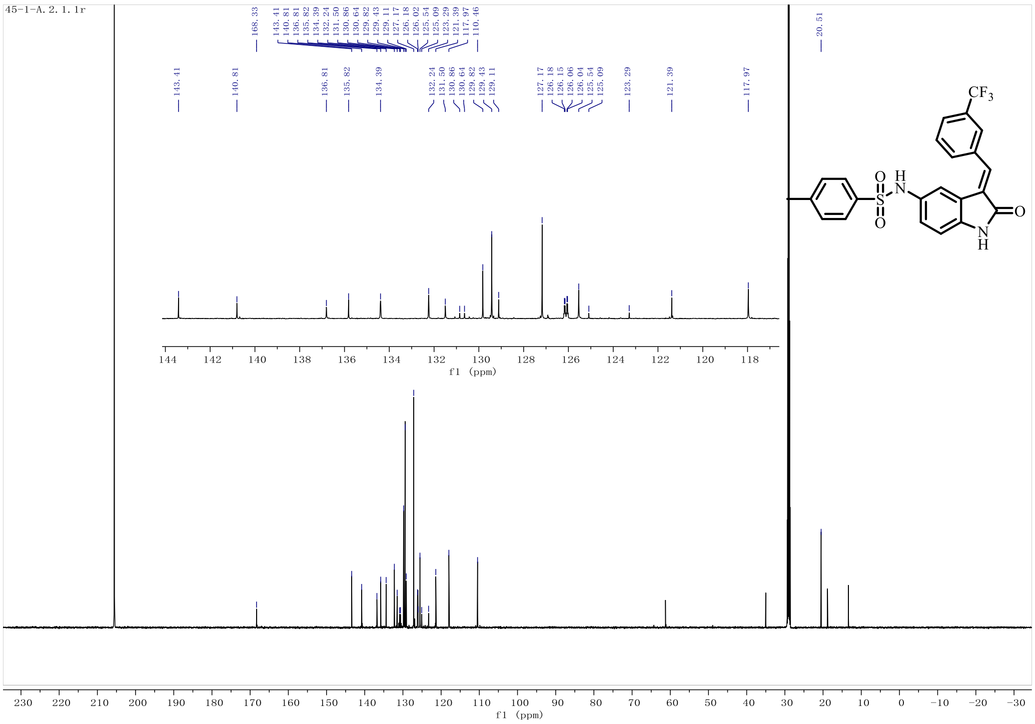

Figure S30-2. ^{13}C NMR spectrum (150 Hz, CD3OCD3) of compound **9f**

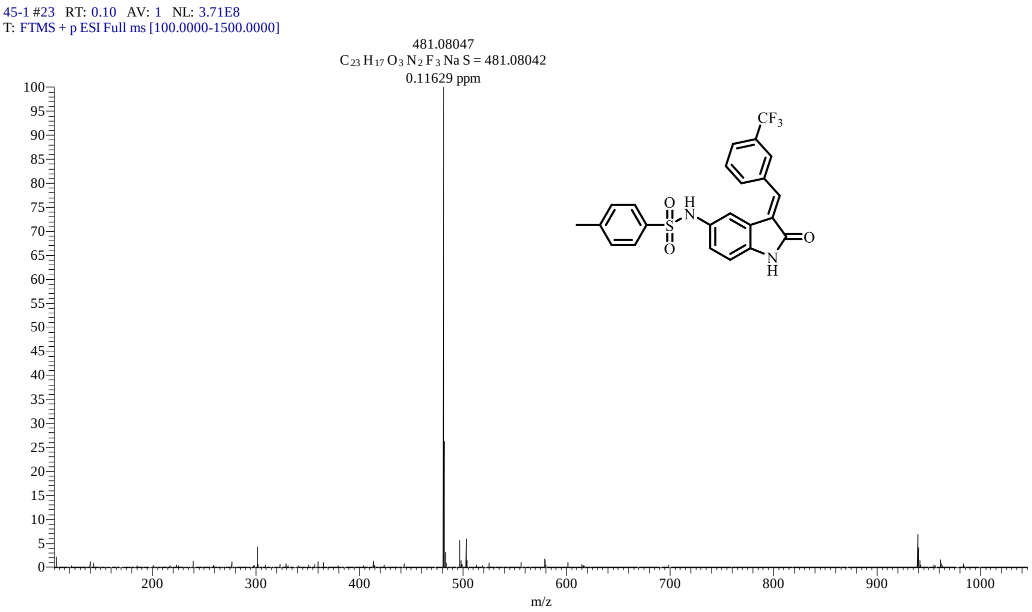

Figure S30-2. HRMS of compound **9f**

(*E*)-4-methyl-N-(2-oxo-3-(3-(trifluoromethyl)benzylidene)indolin-5-yl)benz-enesulfonamide (9g).

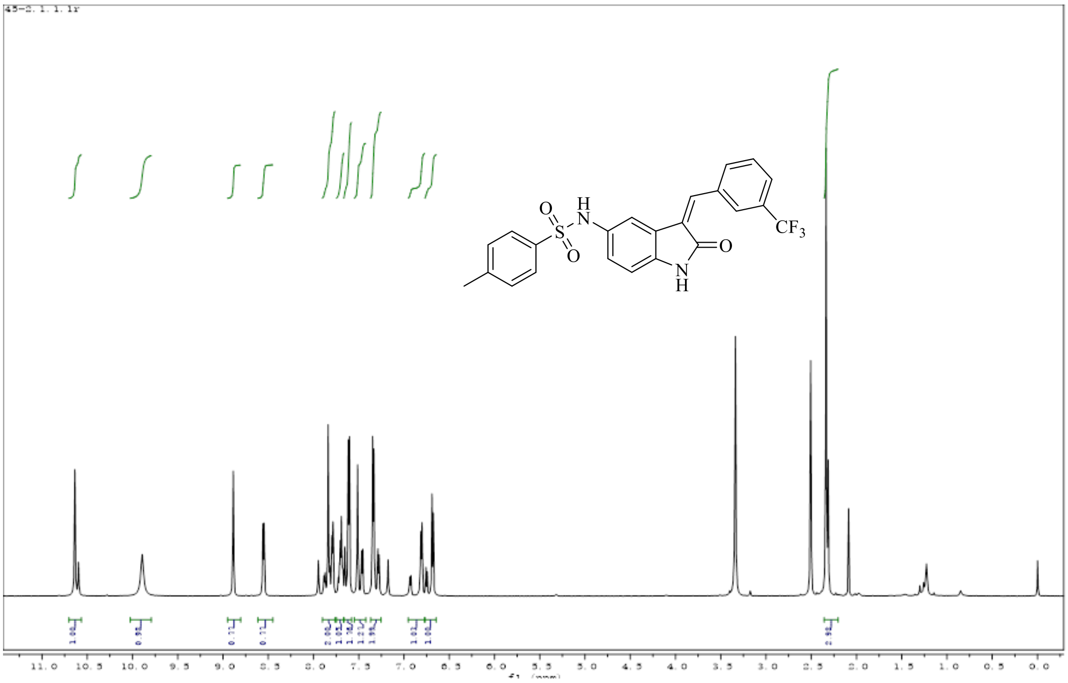

Figure S31-1. ^{1}H NMR spectrum (600 Hz, DMSO) of compound **9g**

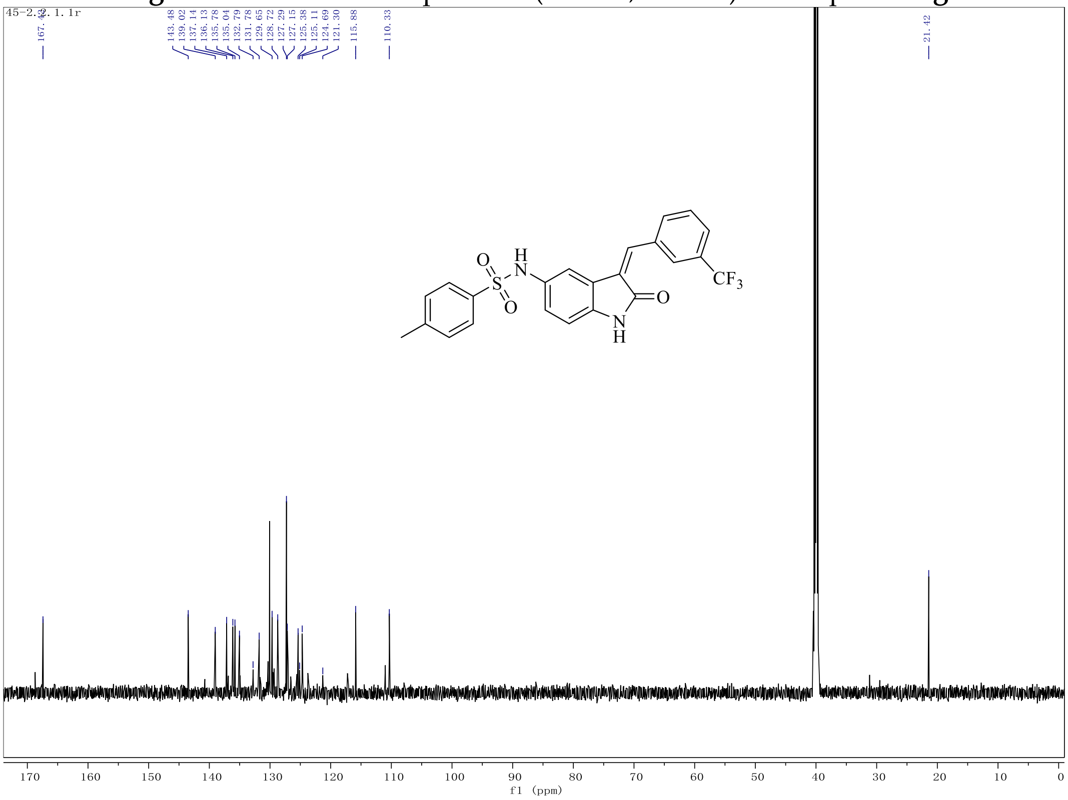

Figure S31-2. ^{13}C NMR spectrum (150 Hz, DMSO) of compound **9g**

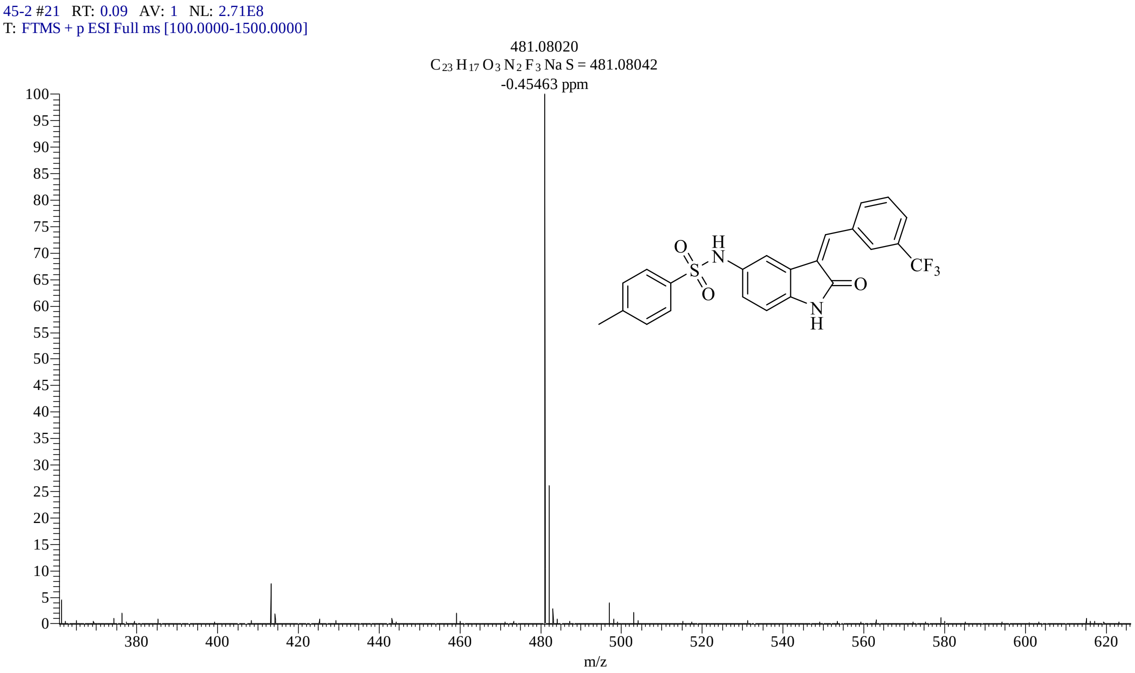

Figure S31-2. HRMS of compound **9g**

5-((3,5-bis(trifluoromethyl)benzylidene)amino)indolin-2-one (9i)

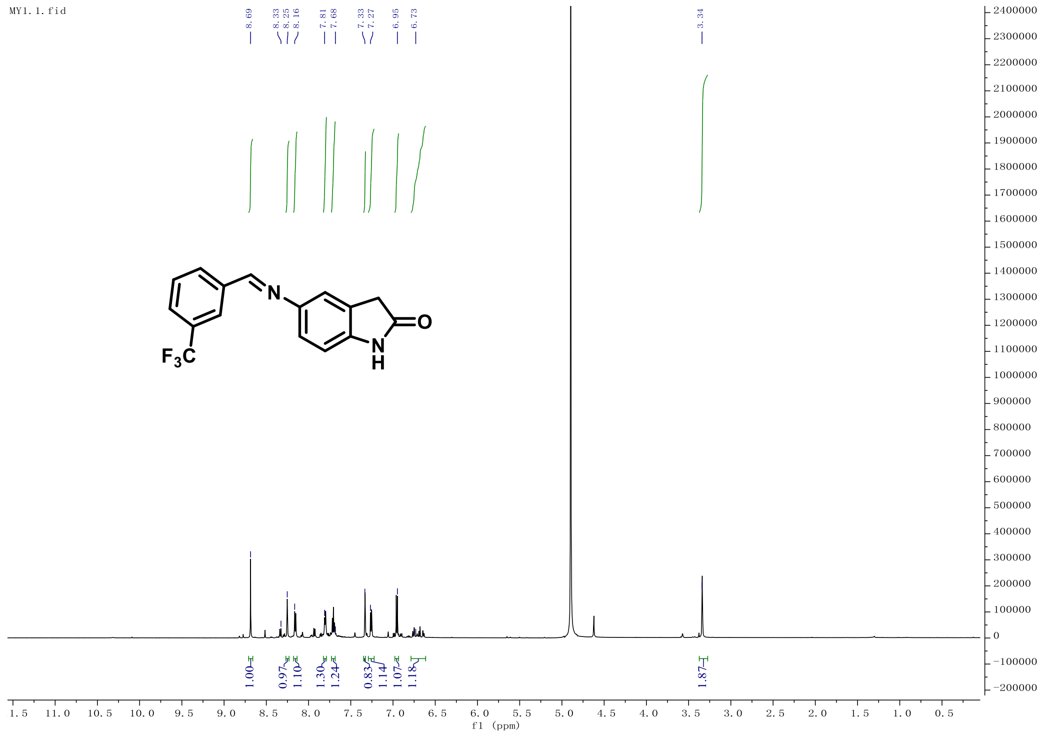

Figure S32-1. ^{1}H NMR spectrum (600 Hz, CD_3OD) of compound **9h**

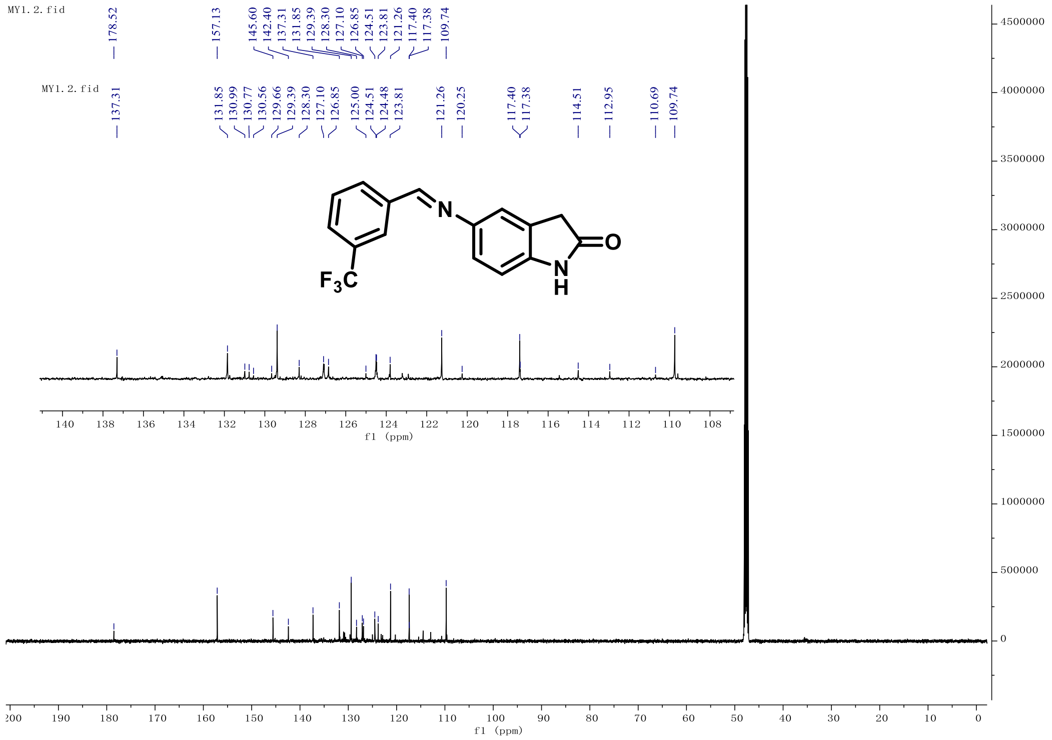

Figure S32-2. ^{13}C NMR spectrum (150 MHz, CD_3OD) of compound **9h**

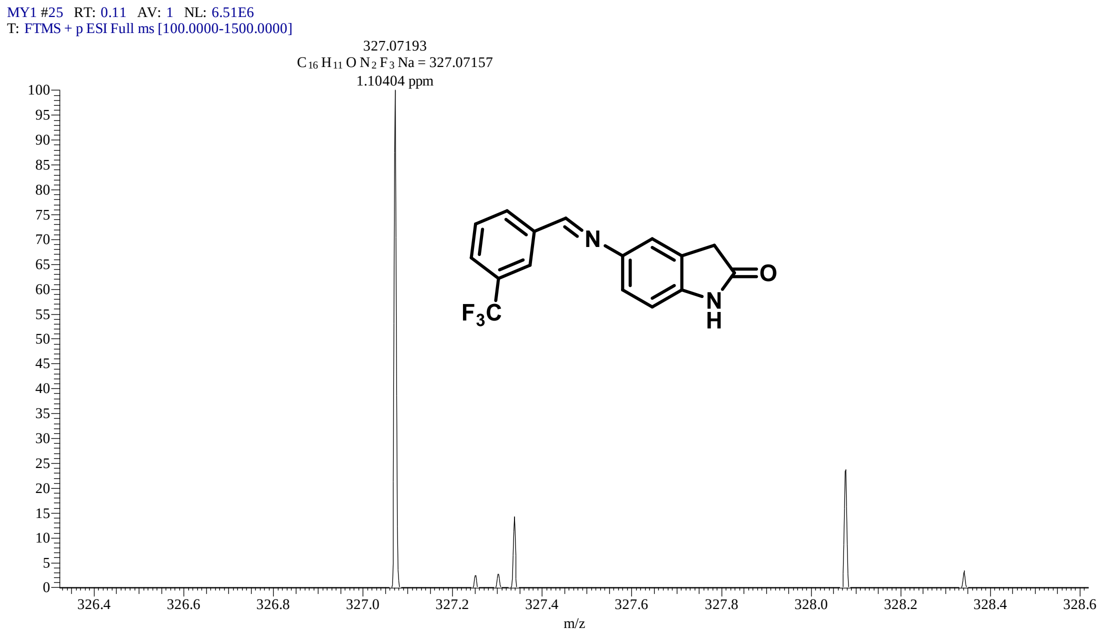

Figure S32-3. HRMS of compound **9h**

5-((3,5-bis(trifluoromethyl)benzylidene)amino)indolin-2-one (9i)

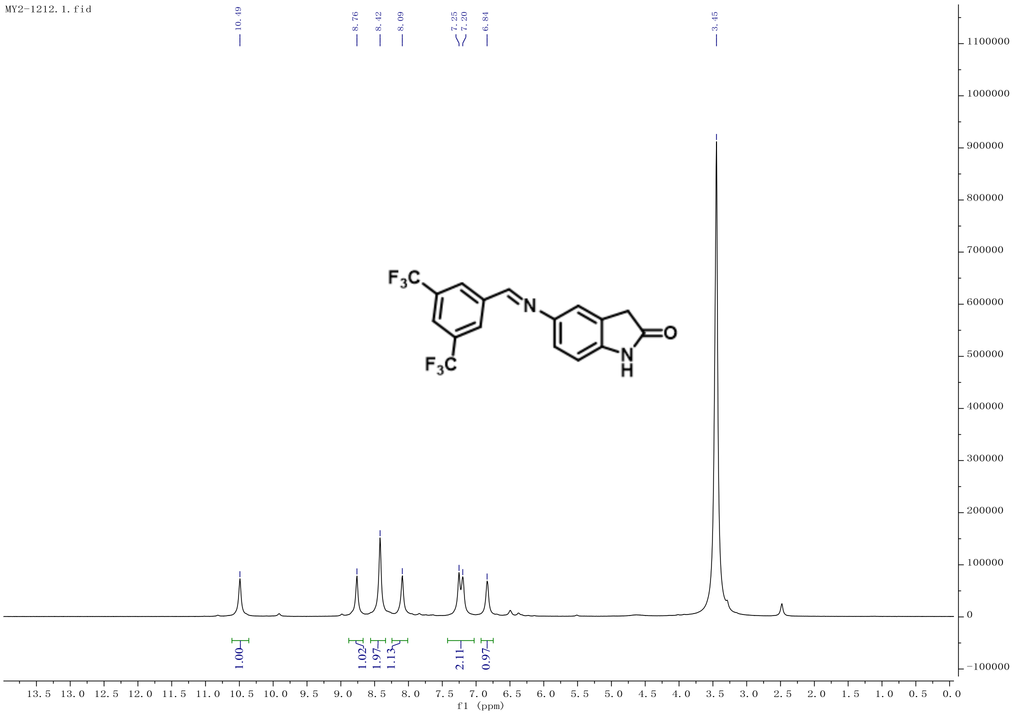

Figure S33-1. ^{1}H NMR spectrum (600 Hz, DMSO) of compound **9i**

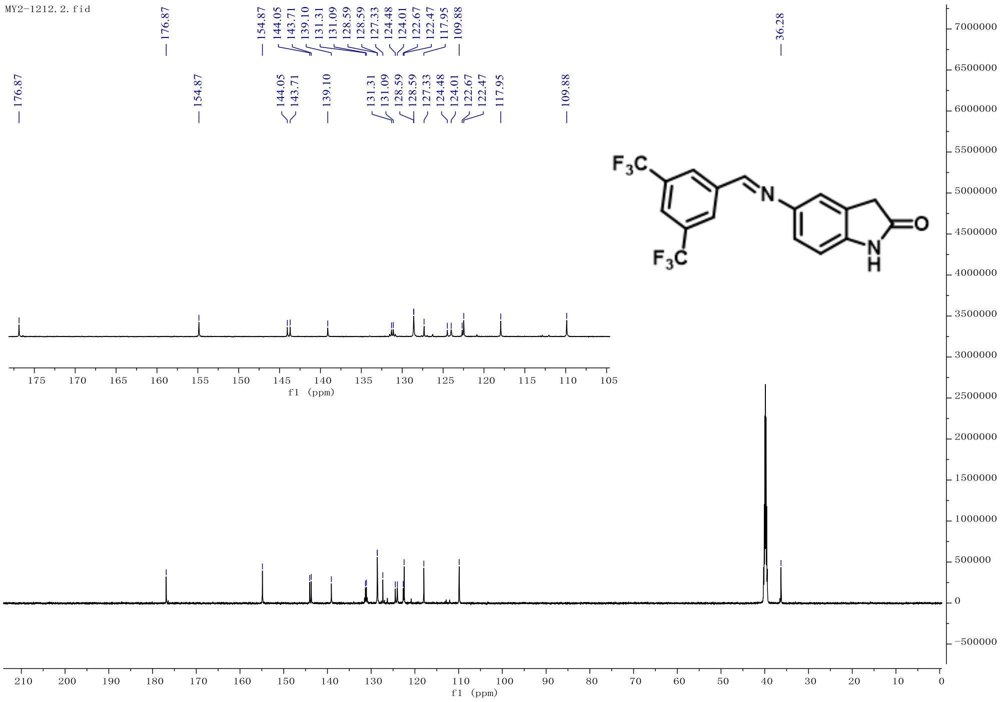

Figure S33-2. ^{13}C NMR spectrum (150 Hz, DMSO) of compound **9i**

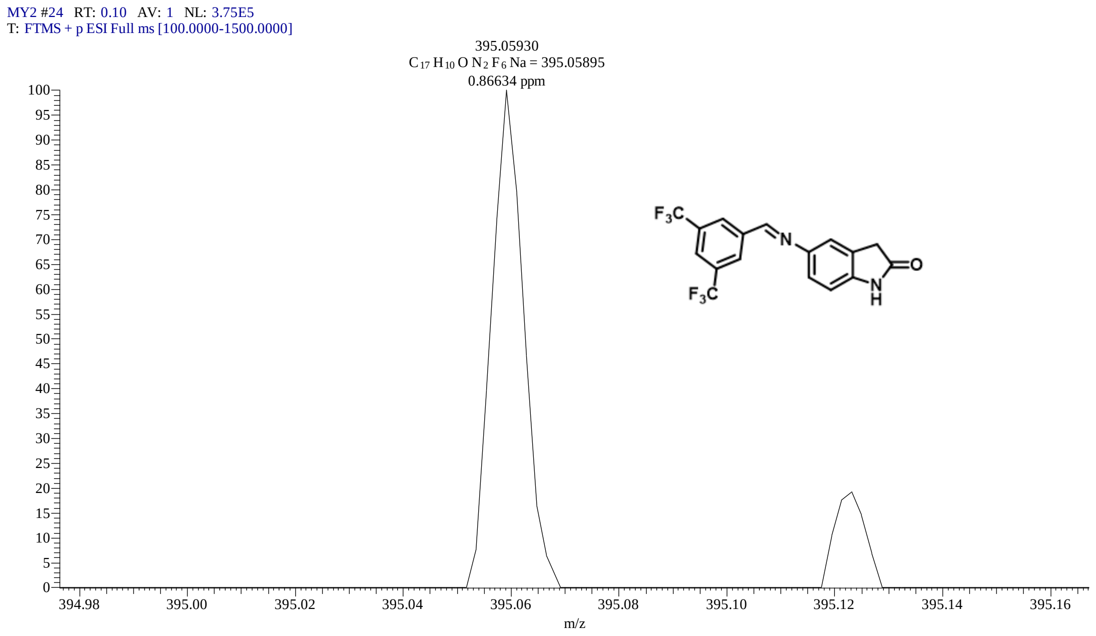

Figure S33-3. HRMS of compound **9i**
